# Effectiveness of digital interventions for eight mental disorders: A meta-analytic synthesis^☆^

**DOI:** 10.1016/j.invent.2025.100860

**Published:** 2025-07-11

**Authors:** Mathias Harrer, Clara Miguel, Lingyao Tong, Paula Kuper, Antonia A. Sprenger, Yuki Furukawa, Yingying Wang, Wouter van Ballegooijen, Marketa Ciharova, Olga M. Panagiotopoulou, Ioana Cristea, Jessica L. Hamblen, Paula P. Schnurr, Heleen Riper, Marit Sijbrandij, Eirini Karyotaki, Annemieke van Straten, Toshi A. Furukawa, Davide Papola, Stefan Leucht, Pim Cuijpers, on behalf of the Metapsy consortium

**Affiliations:** a Department of Clinical, Neuro and Developmental Psychology, World Health Organization Collaborating Center for Research and Dissemination of Psychological Interventions, Amsterdam Public Health Research Institute, Vrije Universiteit, Amsterdam, The Netherlands; b Section for Evidence-Based Medicine in Psychiatry and Psychotherapy, Department of Psychiatry and Psychotherapy, School of Medicine and Health, Technical University of Munich, Munich, Germany; c Department of Clinical, Neuro and Developmental Psychology, Amsterdam Public Health research institute, Vrije Universiteit Amsterdam, the Netherlands; d Institute of Social Medicine and Health Systems Research (ISMHSR), Medical Faculty, Otto von Guericke University Magdeburg, Magdeburg, Germany; e Department of Neuropsychiatry, University of Tokyo, Tokyo, Japan; f Department of General Psychology, University of Padova, Italy; g National Center for PTSD, White River Junction, VT, USA; h Department of Psychiatry, Geisel School of Medicine at Dartmouth, USA; i Department of Clinical, Neuro- and Developmental Psychology, WHO Collaborating Center for Research and Dissemination of Psychological Interventions, Amsterdam Public Health research institute, Vrije Universiteit Amsterdam, Amsterdam, The Netherlands; j Department of Clinical Psychology, University of Amsterdam, Amsterdam, The Netherlands; k Kyoto University Office of Institutional Advancement and Communications, Kyoto, Japan; l WHO Collaborating Centre for Research and Training in Mental Health and Service Evaluation, Department of Neurosciences, Biomedicine and Movement Sciences, Section of Psychiatry, University of Verona, Verona, Italy; m Department of Clinical, Neuro and Developmental Psychology, Amsterdam Public Health research institute, WHO Collaborating Center for Research and Dissemination of Psychological Interventions, Vrije Universiteit Amsterdam, the Netherlands; n Babeș-Bolyai University, International Institute for Psychotherapy, Cluj-Napoca, Romania

**Keywords:** Meta-analysis, Psychotherapy, Mental disorders, Systematic reviews, Meta-analytic research domain

## Abstract

**Objectives:**

In this unified series of meta-analyses, we integrate the effects of digital interventions in adults with mental disorders compared to inactive controls. We cover eight indications: depressive disorder, insomnia, specific phobias, generalized anxiety, panic, social anxiety, obsessive-compulsive, and posttraumatic stress disorder.

**Methods:**

Digital intervention trials in patients with a diagnosed mental disorder (confirmed by clinical interviews) were extracted from the Metapsy living databases for psychological treatments. Standardized meta-analyses were conducted to pool effects for each disorder, as well as separately for guided and unguided treatments. We also examined study dropout rates, conducted meta-regression analyses stratified by disorder, and identified treatments that have since become available as prescribable digital therapeutics in routine care.

**Results:**

In total, 168 studies (22,144 patients) were included. Moderate effect sizes were observed for PTSD (*g* = 0.57), depression (*g* = 0.62), and obsessive-compulsive disorder (*g* = 0.68). Large effects emerged for generalized anxiety (*g* = 0.80), social anxiety (*g* = 0.84), insomnia (*g* = 0.94), panic disorder (*g* = 1.05), and specific phobias (*g* = 1.18). Pooled study dropout rates were generally moderate (≤20 %), but higher in intervention arms (*RR* = 1.13–2.66). Trials with low risk of bias and care-as-usual comparisons were limited across indications. We found 16 trials evaluating a prescribable digital therapeutic (*g* = 0.33–1.60).

**Conclusions:**

Digital interventions can be effective across a wide range of diagnosed mental disorders. For some indications, more high-quality trials and comparisons against care-as-usual are needed to confirm the robustness of the effect, particularly for unguided treatments. Digital interventions are increasingly commercialized as prescribable digital therapeutics. Rising industry involvement may present both opportunities and new challenges for the field.

## Highlights

• Digital interventions are effective for eight common mental disorders.
• Largest effects were observed for anxiety disorders and insomnia.
• Guided interventions show the most robust evidence base.
• Study dropout rates were moderate, but higher in intervention arms

## 1 Introduction

Mental disorders are highly prevalent worldwide, affecting one in five individuals in any given year (Kessler et al., 2009; WHO, 2022). They are associated with substantial losses in quality of life and role functioning, increased medical comorbidity, early mortality, as well as enormous societal costs (Cuijpers et al., 2014; Fergusson and Woodward, 2002; Hare et al., 2014; Wang et al., 2020; Whiteford et al., 2013). Projections indicate that, by 2030, mental disorders will make up more than half of the economic burden attributable to non-communicable diseases, estimated at 6 trillion USD worldwide (Bloom et al., 2012; Knapp and Wong, 2020).

Evidence-based treatments are available for most mental disorders, including various psychological interventions (Carpenter et al., 2018; Cuijpers et al., 2023; Karyotaki et al., 2021; Mendes et al., 2008; Olatunji et al., 2013; Papola et al., 2023, Papola et al., 2024); but they remain underused. For most mental disorders, even in high-income countries, <50 % of patients with mental disorders receive minimally adequate treatment (Alonso et al., 2018; Evans-Lacko et al., 2018; Mack et al., 2014; Wang et al., 2007). Even if patients are treated eventually, the preceding duration of untreated mental illness typically ranges between 6 and 8 years for mood disorders, and between 9 and 23 years for anxiety disorders (Wang et al., 2005).

Many of these challenges could potentially be addressed by digital psychological interventions (i.e., web-based, mobile- or smartphone-delivered interventions; Andersson, 2016; Fuhrmann et al., 2023; Weisel et al., 2019). Digital interventions can be more easily disseminated (e.g. through the Internet) than face-to-face treatments, offer greater anonymity and flexibility for their users, and may be attractive for patients who would not otherwise seek help (Ebert et al., 2018). In low and middle-income countries (LMICs), the greater scalability of digital self-help could be crucial to increase treatment coverage among patients with very limited access to mental health care (Karyotaki et al., 2023).

Digital treatments have been intensively studied over the last two decades. There is now a large and ever-increasing body of evidence supporting the efficacy of such interventions for common mental health problems, including for depression (Moshe et al., 2021), anxiety disorders (Pauley et al., 2023), posttraumatic stress disorder (PTSD; Tng et al., 2024), obsessive-compulsive disorder (OCD; Hiranandani et al., 2023), and insomnia (van Straten et al., 2018). For several indications, there is also evidence that digital interventions are not inferior in their efficacy compared to face-to-face treatments, provided that patients are willing to partake in such a format (Carlbring et al., 2018; Chow et al., 2022; Hedman-Lagerlöf et al., 2023; Kambeitz-Ilankovic et al., 2022; Knutzen et al., 2024).

These positive findings have led to an increased adoption of digital interventions in routine care settings. In several countries, digital interventions can now be prescribed by health professionals as part of standard care (Ferrante et al., 2024; Phan et al., 2023; Schmidt et al., 2024). In regulatory contexts, such interventions are now referred to as “prescription digital therapeutics” (DTx; Brezing and Brixner, 2022). For depression, recent treatment guidelines also support the provision of digital interventions as part of a guided self-help concept, and particularly as a low-threshold treatment for patients with milder symptoms (Kendrick et al., 2022; Malhi et al., 2021; Bundesärztekammer (BÄK) et al., 2022).

Many meta-analytic reviews have summarized the efficacy of digital interventions in the past, focusing on specific disorders (Clarke et al., 2019; Moshe et al., 2021; Pauley et al., 2023; Tng et al., 2024) or target groups (Harrer et al., 2019a; Diel et al., 2024; Schouten et al., 2022). There are also umbrella and narrative reviews that have summarized meta-analyses on digital intervention effects across mental disorders (Andersson, 2016; Ebert et al., 2018). However, no study has so far examined these effects within a single, unified meta-analytic approach, and with a standardized methodology for study selection, data extraction, risk of bias assessment, and statistical aggregation. Such a “supersized” meta-analysis would allow to (i) provide an up-to-date, high-level quantitative synthesis of the field, (ii) increase the comparability of effect estimates across indications, (iii) examine common predictors of digital intervention effects across various disorders, and (iv) determine mental health problems for which existing evidence is comparatively weak or unconvincing.

Such large-scale syntheses have been logistically very challenging in the past. The Metapsy initiative (metapsy.org) has overcome many of these difficulties by creating standardized, living meta-analytic databases of psychological treatments for various mental health issues, known as “meta-analytic research domains” (MARDs; Cuijpers et al., 2022). In the present study, we leverage this infrastructure to synthesize the benefits of digital interventions in adults with diagnosed mental disorders. Specifically, we will (i) quantify the pooled treatment effects across eight mental health conditions (depression, primary insomnia, generalized anxiety disorder (GAD), social anxiety disorder (SAD), panic disorder, specific phobias, OCD, and PTSD) compared to inactive control groups; (ii) calculate subgroup-specific effects for interventions with and without human guidance, and test the robustness of the existing evidence; (iii) pool (differential) study dropout rates; (iv) examine the effect of study and intervention-related moderators across indications in a joint model; and (v) identify evaluations of digital interventions that have since become available as prescription DTx.

## 2 Materials and methods

### 2.1 Search strategy & selection criteria

A protocol and statistical analysis plan (SAP) for this study was preregistered using the Open Science Framework (OSF; osf.io/nf7dz). Open data and materials were made available on Github (github.com/mathiasharrer/meta-dtx). Where applicable, we report the results of this standardized series of meta-analyses according to the Preferred Reporting Items for Systematic reviews and Meta-Analyses (PRISMA) statement (Page et al., 2021).

All included studies were extracted from living meta-analytic databases compiled by the Metapsy initiative (see docs.metapsy.org/databases for an overview). As MARDs, these living databases cover a comprehensive set of intervention types and comparators for each indication, and are updated at least yearly (Cuijpers et al., 2022). The deadline for search updates included in the current meta-analysis was January 1st, 2024. All Metapsy MARDs are updated using systematic searches in PubMed, PsycINFO, and Embase, by combining terms indicative of each of the disorders and psychotherapies, and with filters for randomized trials (see supplement S1 for full search strings). Several other bibliographic databases are searched depending on the disorder (see documentation entries at docs.metapsy.org/databases for further details). Separate searches are conducted for MARDs covering depressive disorders, insomnia, PTSD, and OCD. A combined update search is performed for four anxiety disorders: panic disorder with or without agoraphobia, GAD, SAD, and specific phobia. In all living databases, each step of the study search (title and abstract screening, full-text selection, inclusion of studies), data extraction, and coding (including risk of bias assessments) is conducted by two independent researchers. Disagreements are resolved through discussion and, if needed, consultation with a third (senior) researcher.

For the present analysis, we considered all Metapsy databases covering the following indications: depressive disorders (unguided and guided interventions; Cuijpers et al., 2023; Tong et al., 2024), SAD (de Ponti et al., 2024), GAD (Papola, 2024), panic disorder (with and without agoraphobia; Papola, 2023; Papola et al., 2022), specific phobia, OCD (Wang et al., 2024a, Wang et al., 2024b), PTSD (National Center for PTSD, US Department of Veterans Affairs, 2023), and insomnia. For insomnia, we integrated a new living database based on a previous synthesis by Furukawa et al. (2024), and included it in the present study. A meta-analysis tool and online documentation entry for this database will be released on the Metapsy platform in 2025.

Trials in the living Metapsy databases were included in the current study if they compared (i) a digital intervention with (ii) a control group (waiting list, CAU, other inactive controls such as attention placebo). We defined digital interventions as treatments in either an offline or online setting (i.e., as computerized-, online-, virtual reality-, or mobile-based treatments), whereby digital contents constitute the main component of the intervention. Digital interventions (iii) could be both guided (i.e., providing therapeutic support and/or content-related feedback by trained personnel) or unguided. All trials (iv) had to be conducted in adults (≥18 years) with (v) a diagnosed mental disorder, (vi) as confirmed by clinical interviews. Criteria (v) and (vi) were employed to ensure consistency across all disorders, meaning that only cross-indication effects among individuals with a confirmed diagnosis are compared (as opposed to elevated symptom scores alone, which can be more or less predictive of diagnostic status).

We excluded trials if (i) patients were only included based on elevated scores on self-report instruments; (ii) digital treatment components were only provided as an adjunct to face-to-face psychotherapy (including “blended treatments”); (iii) conventional face-to-face therapies were provided via digital means (e.g., videoconference) with no other digital components; (iv) treatment focused exclusively on cognitive functions (e.g., memory, attention, cognitive bias), (v) the sample focused on children and/or adolescents (<18 years); and (vi) if caregivers of patients were the primary intervention target, not patients themselves. For each database, eligibility assessments were conducted by two independent researchers (AAS and PK, CM and MH, LT and MH, LT and CM), and disagreements were resolved through discussion.

### 2.2 Risk of bias & data extraction

In all included databases, risk of bias was assessed using version 2 of Cochrane's risk of bias tool for randomized trials (RoB-2; Sterne et al., 2019) with two independent raters. The RoB-2 tool comprises five domains: (i) bias arising from the randomization process; (ii) bias due to deviations from intended interventions; (iii) bias due to missing outcome data; (iv) bias in the measurement of outcome; and (v) bias in the selection of the reported result. Based on these domains, a study is judged as showing overall “low risk of bias”, “high risk of bias” or “some concerns”.

As part of the Metapsy “data standard” (docs.metapsy.org/data-preparation/format), a core set of study characteristics is routinely extracted in all living databases (e.g. mean age, proportion of women in the sample, intervention format, type of comparator, treatment modality, human support, etc.). Interventions are coded as “guided” if human content-related and/or therapeutic support was provided during treatment. For the present study, we additionally categorized the type of digital intervention (defined as online/web-based, mobile−/smartphone-based, computer-based, virtual reality-based, or other/mixed). Among online/web-based interventions, we also included treatments in which digital contents were enhanced by additional mobile features (e.g., SMS messages to boost adherence, tracking applications). Furthermore, we also screened all unguided intervention trials to identify a subset of “purely unguided” formats. Interventions were coded as purely unguided if no mechanism for (human or automatized) encouragement was reported (e.g., motivational messages, e-mail reminders, prescheduled telephone calls, visits to monitor adherence).

Lastly, we also determined if evaluated interventions have since become available as a prescribable DTx. To the best of our knowledge, there is currently no systematic cross-national register for prescribable DTx. Therefore, available therapeutics and their evidence base were extracted from previous reviews (Phan et al., 2023; Wang et al., 2023; Goeldner and Gehder, 2024; Nomura, 2024) as well as national registers (BfArM, 2024), and were subsequently matched with the eligible trials in this study. In the supplement (S2), we provide further details on our definition and identification of prescription DTx.

### 2.3 Outcomes

For each included comparison between a digital intervention and control, we calculated intervention efficacy using the post-treatment small sample bias-adjusted standardized mean difference (SMD; Hedges' *g*). All outcomes indicating the symptoms of the disorder were included. When means and standard deviations were not reported, we used change scores, converted binary outcomes to the SMD (Chinn, 2000) or used other statistics (e.g., *p*-value, *t*-value) to calculate the target outcome measure. As a last step, all SMD values were recoded so that effects with a positive sign always indicate results favoring the intervention. As secondary outcomes, we also calculated treatment acceptability, as the logit-transformed proportion of study drop-out for any reason in both arms, and the (log-)relative risk of dropping out in the digital intervention arm versus the control group. Dropout proportions were logit-transformed into log-odds to produce variance-stabilized estimates assumed to follow a normal distribution. This is a prerequisite for standard random-effects meta-analysis (Harrer et al., 2021, chap. 13; Stijnen et al., 2010). Both logit-transformed proportions and log-relative risks were re-transformed to their original scale after pooling.

### 2.4 2.3 Quantitative synthesis (meta-analysis)

In our main analysis, we pooled effect estimates indicating the benefits of digital interventions on symptom severity separately for each disorder (depression, GAD, panic disorder, specific phobia, SAD, OCD, PTSD, and insomnia). To accommodate the nested data structure (multiple effect estimates in studies), a random-effects three-level correlated and hierarchical effects (CHE) model was used throughout (Pustejovsky and Tipton, 2022). A constant within-cluster sampling correlation of *ρ* = 0.6 was assumed in all analyses, and we used cluster-robust variance estimation (CRVE; “CR2” estimator). Restricted maximum likelihood (REML) was used to maximize the models. Heterogeneity was examined by calculating 95 % prediction intervals for the pooled effect. We also calculated a three-level model equivalent of *I*^2^, indicating the amount of variability not attributable to sampling error (Cheung, 2014).

Several sensitivity analyses were conducted to assess the robustness of the effect. First, we calculated the subgroup-specific effect if only comparisons with care-as-usual (CAU) conditions were considered, and when only comparisons with a low risk of bias rating were included. Second, we recalculated the pooled effect when effect sizes were first pre-aggregated on a study level. Effects were then pooled using a conventional inverse-variance random-effects model, and the Knapp-Hartung method was used to obtain robust confidence intervals and significance tests of the overall effect (IntHout et al., 2014). This approach has been shown to be mathematically equivalent to the frequently employed “correlated effects” (CE) model if certain assumptions are met (Pustejovsky and Chen, 2023). Third, we pooled effect sizes while excluding influential cases, defined by the diagnostics proposed by Viechtbauer and Cheung (2010). Fourth, we conducted one meta-analysis in which we only included the smallest effect size from a study, and another meta-analysis in which only the largest effect size from a study was included. Lastly, we applied three methods adjusting for potential small-study effects and/or selective publication: Duval and Tweedie's (2000) trim and fill procedure, a “limit meta-analysis” (Rücker et al., 2011), and a three-parameter selection model (McShane et al., 2016; with the selection cut-point set at the conventional significance threshold).

For all disorders, we repeated the analyses listed above specifically for guided and unguided interventions; but only if at least three effects were available. We also conducted a subgroup-specific analysis in which we only considered “purely unguided” interventions. As secondary outcomes, we pooled the arm-specific dropout rates using a generalized linear mixed model (GLMM; Stijnen et al., 2010), and the log-risk ratios for differential dropout between arms using a log-normal-normal pooling model.

To examine predictors of differential treatment effects across indications, we extended the main CHE model to perform multivariable meta-regression. This model included all eligible trials, with stratification terms for disorder. The following predictors were included: world region (Europe, East/Southeast Asia, Australia, Middle East, North America); type of intervention (cognitive behavior therapy, behavioral activation, psychodynamic therapy, exposure, problem-solving, “third-wave” therapies, other/mixed); type of comparator (CAU, waitlist, psychoeducation, psychological placebo, other inactive control); risk of bias (“high” or “some concerns”, “low”); publication year; percentage of females in the sample, mean age, guidance (unguided, guided), intervention modality (web-/computer-based, mobile-/smartphone-based, virtual reality-based, other/mixed). Additionally, we also fitted the same meta-regression model, but only considered one predictor each time. For this analysis, we also considered recruitment (clinical, community, other) as a predictor. This variable was omitted in the multiple meta-regression model since it is currently systematically missing in the insomnia dataset.

All analyses were conducted in R version 4.2.0. We used the “metapsyTools” package (Harrer et al., 2022), which was specifically developed for the Metapsy databases. This package imports functionality of the “meta” (Balduzzi et al., 2019), “metafor” (Viechtbauer, 2010), and “dmetar” (Harrer et al., 2019b) packages. Certainty of the evidence provided by our meta-analyses was evaluated using the GRADE framework (Guyatt et al., 2008) with two independent raters (PK, MH).

## 3 Results

### 3.1 Study selection & inclusion

Searches across all disorders resulted in a total of 106,800 records (71,944 after removal of duplicates), 10,823 full-text papers retrieved, and *K* = 168 included studies (see Table 1). PRISMA-type search flow charts for each disorder are presented in S3 in the supplement. The number of included studies ranged from 49 (for depression) to 5 (for specific phobias). The total number of effect comparisons across disorders was *n*_eff_ = 388. In total, 22,144 patients were randomized in the included studies (12,079 into digital intervention arms, and 10,065 into control conditions). References of the included studies are provided in S4 in the supplement.

**Table 1:** Study characteristics.

	Depression	Panic	SAD	GAD	Phobia	PTSD	OCD	Insomnia	Total
***Study search***									
- Identified records	35,518	21,193	21,193	21,193	21,193	27,679	12,449	9961	106,800
- After removal duplicates	25,309	14,682	14,682	14,682	14,682	15,493	8766	7694	71,944
- Full-texts assessed	4439	1490	1490	1490	1490	2302	581	2011	10,823
***Included studies***									
- Total number of studies	49	15	26	12	5	15	9	37	168
- Total number of comparisons	150	18	92	43	7	21	14	43	388
***Included patients***									
- Total number of patients	6326	917	2098	933	188	1685	739	9258	22,144
- Number of patients in therapy	3461	491	1212	508	94	913	368	5032	12,079
- Number of patients in control	2865	426	886	425	94	772	371	4226	10,065
***Study characteristics***									
- Clinical recruitment; *n* (%)	11 (22.4)	1 (6.7)	1 (3.8)	0 (0.0)	0 (0.0)	3 (20.0)	3 (33.3)	-a	19 (14.5)
- Mean age (*M*, *SD*)	39.6 (8.2)	37.0 (2.7)	33.3 (6.1)	39.7 (11.7)	32.8 (11.2)	39.5 (11.9)	33.7 (4.9)	46.1 (7.9)	39.3 (8.1)
- Proportion women (*M*, *SD*)	0.75 (0.12)	0.71 (0.07)	0.63 (0.09)	0.78 (0.09)	0.69 (0.20)	0.70 (0.26)	0.58 (0.13)	0.70 (0.18)	0.70 (0.14)
- Treatment modality; *n* (%)									
- Web/Computer-based^b^	44 (89.8)	14 (93.4)	17 (65.4)	10 (76.9)	1 (20.0)	13 (86.7)	8 (88.9)	30 (81.1)	137 (81.5)
- Mobile-based	5 (10.2)	1 (6.7)	2 (7.7)	3 (23.1)	0 (0.0)	2 (13.3)	1 (11.1)	4 (10.8)	18 (10.7)
- Virtual Reality	0 (0.0)	0 (0.0)	7 (26.9)	0 (0.0)	4 (80.0)	0 (0.0)	0 (0.0)	0 (0.0)	11 (6.5)
Comparator; *n* (%)									
- Care As Usual	16 (32.7)	1 (6.7)	0 (0.0)	2 (15.4)	0 (0.0)	2 (13.3)	1 (11.1)	9 (24.3)	31 (18.5)
- Waitlist	25 (51.0)	13 (86.7)	25 (96.2)	9 (69.2)	4 (80.0)	10 (66.7)	5 (55.6)	25 (67.6)	116 (69.1)
- Country; *n* (%)^c^									
- Europe	29 (59.2)	9 (60.0)	14 (53.8)	4 (30.8)	3 (60.0)	5 (33.3)	2 (22.2)	22 (59.5)	88 (52.4)
- East/Southeast Asia	3 (6.1)	0 (0.0)	3 (11.5)	0 (0.0)	1 (20.0)	2 (13.3)	2 (22.2)	4 (10.8)	15 (8.93)
- Australia	9 (18.4)	6 (40.0)	3 (11.5)	4 (30.8)	1 (20.0)	2 (13.3)	3 (33.3)	1 (2.7)	29 (17.3)
- North America	8 (16.3)	0 (0.0)	6 (23.1)	4 (30.8)	0 (0.0)	6 (40.0)	2 (22.2)	9 (24.3)	35 (20.8)
- Other	0 (0.0)	0 (0.0)	0 (0.0)	1 (7.7)	0 (0.0)	2 (13.3)	0 (0.0)	1 (2.7)	4 (2.4)
***Risk of Bias***									
- Low risk of bias; *n* (%)	8 (16.3)	2 (13.3)	3 (11.5)	5 (38.5)	2 (40.0)	1 (6.7)	0 (0.0)	2 (5.4)	23 (13.7)

a Not individually extracted.

b Includes Internet- and mobile-based interventions (IMIs).

### 3.2 Study characteristics

Study characteristics are summarized in Table 1. The mean age across disorders ranged from 32.8 (*SD* 11.2; phobias) to 46.1 years (*SD* 7.9; insomnia). The majority of participants were women (58 % to 78 %). Web- and computer-based interventions were the most common format among all disorders except specific phobias, where virtual reality applications were more common (80 % of trials). Waitlists were the dominant comparator across all indications (51 % to 96.2 %). CAU comparisons were far less common, and for two disorders (SAD and specific phobias), no CAU-controlled trial was available. Across indications, most trials showed a high risk of bias or “some concerns”. For OCD, no low risk of bias trial could be included, and only one trial for PTSD.

**Fig. 1:**
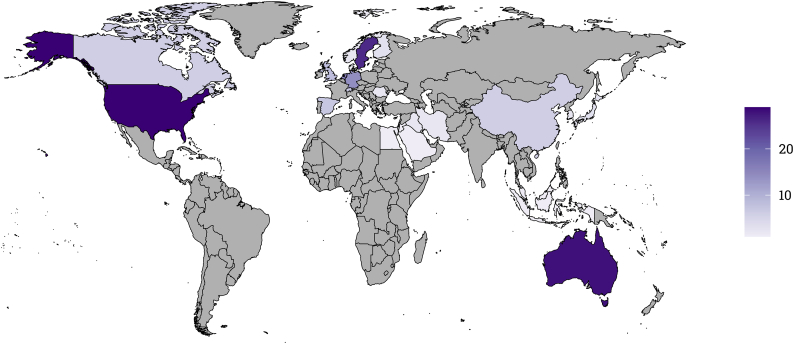
Number of digital intervention trials by country*.* *Note.* The shown numbers are based on studies selected for this meta-analysis from the Metapsy databases for depression, panic disorder, social anxiety disorder, generalized anxiety disorder, specific phobia, posttraumatic stress disorder, obsessive-compulsive disorder, and insomnia (*k* = 168).

Fig. 1 shows the geographical distribution of trials. Studies were predominantly conducted in high-income Western countries. Standardized by one million inhabitants, the most productive countries were Sweden (2.58 trials), Australia (1.14), and the Netherlands (0.93; see S11 in the supplement). Trials were included from all major world regions, except for Latin America, sub-Saharan Africa, and South Asia.

### 3.3 Treatment effects & moderators

**Fig. 2:**
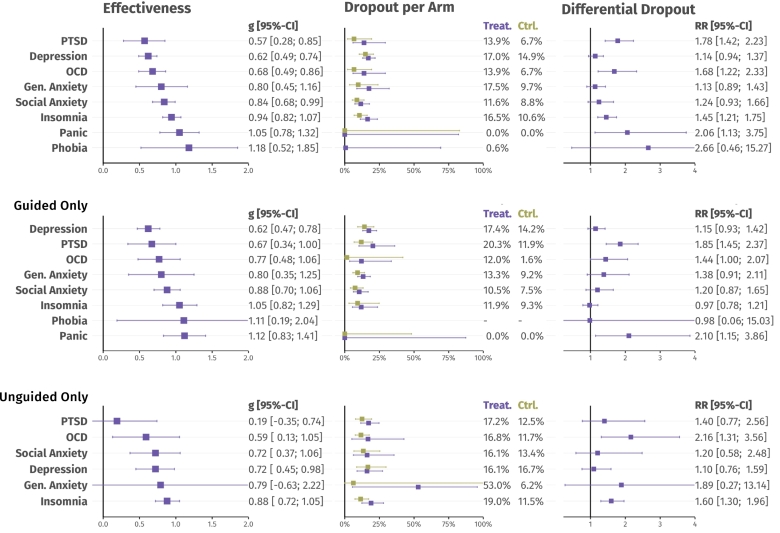
Results of moderator analyses across disorders (meta-regression).

Results of our main analysis are summarized in Table 2 and Fig. 2. We found a significant pooled effect of digital interventions for all eight disorders, but there was some variation in the size of the overall effect. Moderate-to-large effects were found for PTSD (*g* = 0.57), depression (*g* = 0.62) and OCD (*g* = 0.68). Large effects were found for GAD (*g* = 0.80), SAD (*g* = 0.84), insomnia (*g* = 0.94), panic disorder (*g* = 1.05), and specific phobias (*g* = 1.18). Similar significant effects were also found for guided interventions specifically (*g* = 0.62 to 1.12). For unguided interventions, we could only ascertain statistically significant effects for treatments of OCD (*g* = 0.59), SAD (*g* = 0.72), depression (*g* = 0.72), and insomnia (*g* = 0.88). No significant effect emerged for unguided GAD (*g* = 0.79; 95 % CI: −0.63 to 2.22; 5 comparisons) and PTSD interventions (*g* = 0.19; 95%CI: −0.35 to 0.74; 3 comparisons). For panic disorder and specific phobias, not enough studies investigating unguided treatments were included to perform a meta-analysis. Heterogeneity was moderate or high in most analyses (*I*^2^ = 6.9 % to 88.79 %). The prediction interval, indicating the range in which the effect size of future trials is expected to fall, did not include zero for insomnia (overall; guided and unguided interventions), SAD (overall; guided interventions), panic disorder (overall; guided interventions), OCD (overall; guided and unguided interventions) and specific phobias (overall).

**Table 2:** Meta-analytic effects of digital interventions across eight common mental disorders.

	*n*_eff_	*g*	95 %-CI	*I*^2^	95 %-PI	*NNT*
***Depression*** (*k* = 49)						
Overall	150	0.62	[0.49; 0.74]	80.8	[−0.18; 1.41]	4.74
*- CAU comparisons only*	43	0.41	[0.23; 0.59]	79.3	[−0.20; 1.01]	7.61
*- Low RoB studies only*	10	0.57	[0.33; 0.81]	71.0	[−0.13; 1.27]	5.21
Unguided interventions	68	0.72	[0.45; 0.98]	88.7	[−0.31; 1.75]	3.99
*- CAU comparisons only*	20	0.38	[0.13; 0.63]	73.0	[−0.35; 1.11]	8.12
*- Low RoB studies only*	2	0.56	[−2.79; 3.91]	76.5	–	5.34
Guided interventions	82	0.62	[0.47; 0.78]	79.1	[−0.21; 1.46]	4.67
*- CAU comparisons only*	23	0.37	[0.08; 0.66]	67.1	[−0.03; 0.78]	8.40
*- Low RoB studies only*	8	0.57	[0.28; 0.87]	73.8	[−0.24; 1.38]	5.14
***Insomnia*** (*k* = 37)						
Overall	43	0.94	[0.82; 1.07]	80.9	[0.26; 1.63]	2.93
*- CAU comparisons only*	13	0.88	[0.71; 1.04]	80.0	[0.22; 1.53]	3.19
*- Low RoB studies only*	2	0.99	[−1.21; 3.19]	73.1	–	2.79
Unguided interventions	25	0.88	[0.72; 1.05]	85.2	[0.17; 1.60]	3.16
*- CAU comparisons only*	6	0.90	[0.63; 1.17]	77.1	[0.33; 1.47]	3.09
*- Low RoB studies only*	2	0.99	[−1.21; 3.19]	73.1	–	2.79
Guided interventions	18	1.05	[0.82; 1.29]	66.2	[0.32; 1.79]	2.60
*- CAU comparisons only*	7	0.83	[0.52; 1.13]	67.4	[−0.12; 1.77]	3.40
***Social Anxiety Disorder*** (*k* = 26)						
Overall	92	0.84	[0.68; 0.99]	70.9	[0.05; 1.63]	3.35
- *Low RoB studies only*	3	1.22	[−0.80; 3.25]	88.5	[−10.19; 12.63]	2.23
Unguided interventions	27	0.72	[0.37; 1.06]	79.2	[−0.35; 1.78]	4.00
Guided interventions	65	0.88	[0.70; 1.06]	64.8	[0.19; 1.58]	3.15
- *Low RoB studies only*	3	1.22	[−0.80; 3.25]	88.5	[−10.19; 12.63]	2.23
***Posttraumatic Stress Disorder*** (*k* = 15)						
Overall	21	0.57	[0.28; 0.85]	78.1	[−0.39; 1.53]	5.20
- *Low RoB studies only*	2	1.14	[−2.27; 4.56]	74.4	–	2.4
Unguided interventions	3	0.19	[−0.35; 0.74]	0.0	[−0.28; 0.67]	17.0
Guided interventions	18	0.67	[0.34; 1.00]	78.7	[−0.33; 1.66]	4.33
- *Low RoB studies only*	2	1.14	[−2.27; 4.56]	74.4	–	2.4
***Panic Disorder*** (*k* = 15)						
Overall	18	1.05	[0.78; 1.32]	70.1	[0.07; 2.03]	2.61
- *Low RoB studies only*	2	0.65	[−2.74; 4.04]	48.9	–	4.49
Guided interventions	16	1.12	[0.83; 1.41]	68.8	[0.12; 2.13]	2.43
***Generalized Anxiety Disorder*** (*k* = 12)						
Overall	41	0.80	[0.45, 1.16]	79.6	[−0.34, 1.94]	3.52
*- CAU comparisons only*	5	1.68	[−0.93, 4.29]	34.7	[0.86, 2.50]	1.67
*- Low RoB studies only*	5	0.68	[0.06, 1.31]	74.8	[−0.86, 2.23]	4.21
Unguided interventions	5	0.79	[−0.63, 2.22]	81.8	[−0.94, 2.52]	3.56
Guided interventions	36	0.80	[0.35, 1.25]	81.2	[−0.43, 2.03]	3.52
- *Low RoB studies only*	4	0.77	[−0.08, 1.62]	75.1	[−1.50, 3.04]	3.68
***Obsessive-Compulsive Disorder*** (*k* = 9)						
Overall	14	0.68	[0.49; 0.86]	33.3	[0.24; 1.11]	4.27
Unguided interventions	6	0.59	[0.13; 1.05]	6.9	[0.20; 0.97]	5.02
Guided interventions	8	0.77	[0.48; 1.06]	52.5	[0.09; 1.45]	3.68
***Specific Phobia*** (*k* = 5)						
Overall	7	1.18	[0.52; 1.85]	56.6	[0.03; 2.33]	2.31
- *Low RoB studies only*	3	0.96	[0.06; 1.87]	14.4	[−2.44; 4.37]	2.87
Guided interventions	5	1.11	[0.19; 2.04]	64.4	[−0.40; 2.63]	2.46
- *Low RoB studies only*	2	0.90	[−3.33; 5.12]	48.5	–	3.10

*Note*. Separate analyses for guided or unguided interventions were only conducted when at least three effect sizes were available. Sensitivity analyses (low RoB; CAU comparisons only) were only conducted when more than one effect size was available. CAU = care as usual; RoB = risk of bias; *n*_eff_ = number of effect sizes.

When only low risk of bias evidence was considered (see Table 1), a significant effect could only be ascertained for depression (overall: *g* = 0.57; guided interventions: *g* = 0.57), GAD (overall: *g* = 0.68), and specific phobias (overall: *g* = 0.96). When analyses were restricted to comparisons with CAU, a significant effect emerged only for depression (overall: *g* = 0.41; guided: *g* = 0.37; unguided: *g* = 0.38) and insomnia (overall: *g* = 0.88; guided: *g* = 0.83; unguided: *g* = 0.90). Results of all other sensitivity analyses are presented in S5 in the supplement. These analyses mostly corroborated the findings of the main analysis. For panic disorder (overall, guided), GAD (overall, guided), OCD (guided), and specific phobias (overall, guided), limit meta-analyses indicated that effects are not significant anymore after correction for small-study effects.

Effect estimates for “purely unguided” interventions can be found in S6 in the supplement. Enough trials to perform a meta-analysis were only available for depression (8 studies), insomnia (6 studies), and PTSD (3 studies). Point estimates for purely unguided formats were lower than the overall treatment effect (depression: *g* = 0.47 vs. 0.62; insomnia: *g* = 0.74 vs. 0.94; PTSD: *g* = 0.19 vs. 0.57). For depression and insomnia, between-study heterogeneity remained high even when only purely unguided treatments were considered (*I*^2^ = 85.9 % to 87.6 %).

Results of the multiple meta-regression model are presented in Table 3. Several predictors emerged as significant across disorders (while stratifying by indication). First, we found some variations in treatment effects by intervention type. Using CBT-based formats as the reference category, slightly smaller effects were observed for all other formats, but these differences where mostly minor and not significant (Δ_*g*_ = −0.02 to −0.26). However, there was a significantly lower effect for “other/mixed” therapy formats (Δ_*g*_ = −0.51, *S.E.* = 0.209). In terms of modality, there were smaller effects for mobile−/smartphone-based, virtual reality-based, and other/mixed delivery types compared to web- and/or computer-based treatments (Δ_*g*_ = −0.23 to −0.56). However, this difference was only significant for virtual reality-based formats (*p* = 0.036). Results of analyses in which moderators were analyzed on a variable-by-variable basis largely mirrored these findings (see S7 in the supplement), suggesting that the examined variables were largely uncorrelated with each other.

**Table 3:** Results of moderator analyses across disorders (meta-regression).

Moderator	$\hat{\beta }$	S.E.	*t*	*p*
***World Region***				
- Europe	*Ref.*	–	–	–
- East/Southeast Asia	0.130	0.156	0.832	0.406
- Australia	0.157	0.107	1.469	0.143
- Middle East	0.231	0.253	0.912	0.362
- North America	0.009	0.113	0.083	0.934
***Intervention Format***				
- Cognitive Behavior Therapy	*Ref.*	–	–	–
- Behavioral Activation	−0.080	0.222	−0.361	0.718
- Psychodynamic Therapy	−0.020	0.135	−0.151	0.880
- Exposure Therapies	−0.052	0.219	−0.237	0.813
- Problem-Solving Therapy	−0.257	0.336	−0.767	0.443
- Third-Wave Therapies	−0.068	0.160	−0.426	0.670
- Other Therapies	−0.513	0.209	−2.454	0.015
***Comparator***				
- Care As Usual	*Ref.*	–	–	–
- Waitlist Controls	0.129	0.116	1.114	0.266
- Psychoeducation	0.126	0.208	0.605	0.545
- Psychological Placebo	0.106	0.249	0.427	0.670
- Other Inactive Comparators	0.080	0.142	0.566	0.572
***Risk of Bias***				
- “High” or “Some Concerns”	*Ref.*	–	–	–
- “Low”	0.036	0.113	0.318	0.751
***Publication Year***^a^	0.079	0.056	1.410	0.159
***Females*** (% in sample)^a^	0.061	0.035	1.741	0.083
***Age*** (sample mean)^a^	−0.038	0.041	−0.932	0.352
***Guidance***				
- Guided Interventions	*Ref.*	–	–	–
- Unguided Interventions	−0.053	0.064	−0.824	0.411
***Intervention Modality***				
- Internet/Computer-based^b^	*Ref.*	–	–	–
- Mobile/Smartphone-based	−0.226	0.116	−1.953	0.052
- Virtual Reality-based	−0.424	0.202	−2.104	0.036
- Other/Mixed	−0.562	0.316	−1.777	0.076

*Note*. Results based on a multiple meta-regression model stratified by disorder. *Ref.* = reference category.

a Centered and scaled.

b Includes Internet- and mobile-based interventions (IMIs).

Results of the GRADE assessment are provided in S10 in the supplement. Certainty of evidence was judged “very low” for depression, PTSD and panic disorder. A “low” rating was assigned to insomnia, GAD, and OCD. Effects on SAD and specific phobia were assessed to show a moderate certainty. Certainty of evidence was primarily downgraded due to risk of bias (“very serious” for OCD, “serious” otherwise), and effect inconsistency (“serious” for all outcomes except SAD, OCD, and specific phobias).

### 3.4 Study dropout rates

Results on study dropout rates are summarized in Fig. 2 (second and third column), and further details are provided in S7 and S8 in the supplement. Overall, pooled dropout rates in the intervention arms ranged from 0 % (95 %-CI: 0 % to 82.1 %; panic disorder) to 19.3 % (95 %-CI: 11.2 % to 31.2 %; PTSD), and from 0 % (95 %-CI: 0 % to 82.7 %; panic disorder) to 14.9 % (95 %-CI: 10.5 % to 20.5 %; depression) in the control groups. Heterogeneity was high throughout, and prediction intervals were wide. For all indications, pooled risk ratios indicated larger dropout risk in the intervention compared to control (*RR* = 1.13 to 2.66; overall analysis). Slightly lower values were found for guided interventions specifically (*RR* = 0.97 to 2.10) compared to self-guided interventions (*RR* = 1.10 to 2.16). Heterogeneity of the differential dropout rates was low to moderate ($\tau^{2}$=0 to 1.356).

### 3.5 Prescribable digital therapeutics

In total, we identified 16 trials in which a prescribable DTx was evaluated (Berger et al., 2011, Berger et al., 2017; Christensen et al., 2016; Hagatun et al., 2019; Krämer et al., 2022; Lorenz et al., 2019; Nazem et al., 2023; Ritterband et al., 2009, Ritterband et al., 2012; Espie et al., 2012; Felder et al., 2020; Kyle et al., 2020; Cheng et al., 2019; Espie et al., 2019; Watanabe et al., 2023; Schuffelen et al., 2023). For depression, we found one trial evaluating *deprexis* (GAIA AG, Germany; Berger et al., 2011). This trial evaluated a guided and unguided format of the intervention against a waitlist control, with a “some concerns” risk of bias judgement. The calculated effect size for the unguided arm in this trial was *g* = 0.65 (95 %-CI: 0.09 to 1.22), and *g* = 1.13 (95 %-CI: 0.53 to 1.72) for the guided arm. The second identified depression trial evaluated *Selfapy* (Selfapy GmbH; Krämer et al., 2022) as unguided and guided treatment against a waitlist (unguided version: *g* = 1.25, 95 %-CI: 1.06 to 1.43; guided version: *g* = 1.52, 95 %-CI: 1.26 to 1.77). The risk of bias of this trial was rated as high. For anxiety disorders (GAD and panic disorder), we identified one trial evaluating *velibra* (GAIA AG, Germany; Berger et al., 2017), a transdiagnostic unguided treatment for anxiety disorders. This trial was judged at a low risk of bias, with a calculated effect against waitlist control of *g* = 0.33 (95 %-CI: −0.19 to 0.84).

For insomnia, we identified six trials evaluating *Somryst* (*SHUTi*; Pear Therapeutics/Nox Health, USA; Hagatun et al., 2019; Lorenz et al., 2019; Ritterband et al., 2009, Ritterband et al., 2012; Christensen et al., 2016; Nazem et al., 2023). This intervention was evaluated as an unguided treatment, with three trials (50 %) employing a waitlist. Five trials (83.3 %) were judged to show “some concerns”, and one (16.7 %) received a low risk of bias rating (Christensen et al., 2016). The pooled effect of these trials was *g* = 1.14 (95 %-CI: 0.67 to 1.61; *I*^2^ = 76.1; 95 %-PI: *g* = 0.00 to 2.28). Another digital insomnia treatment, *SleepioRx* (Big Health Ltd., UK), was approved by the U.S. Food and Drug Administration in 2024 (FDA, 2024). We identified five trials in total, comparing this intervention as an unguided treatment to waitlists (Espie et al., 2012; Felder et al., 2020; Kyle et al., 2020) and psychoeducation groups (Cheng et al., 2019; Espie et al., 2019). Two of these trials received a low risk of bias rating (Cheng et al., 2019; Kyle et al., 2020); all others were judged to show “some concerns”. The pooled effect was *g* = 0.95 (95 %-CI: 0.77 to 1.13; *I*^2^ = 47.1; 95 %-PI: *g* = 0.60 to 1.30). One identified trial evaluated *Susmed* (Susmed Inc., Japan), a mobile-based treatment for insomnia, against a psychological placebo (sham application; Watanabe et al., 2023). The study received a low risk of bias rating, and the treatment effect was calculated at *g* = 0.79 (95 %-CI: 0.48 to 1.10). One last trial compared *somnio* (mementor DE GmbH; Germany), an unguided digital intervention for insomnia, to a waitlist control (Schuffelen et al., 2023). This trial received a “some concerns” risk of bias rating, with a calculated between-group effect of *g* = 1.60 (95 %-CI: 1.30 to 1.91).

## 4 Discussion

In this study, we used living meta-analytic databases maintained by the Metapsy initiative to synthesize the effects of digital interventions across eight mental disorders (depression, insomnia, SAD, GAD, panic disorder, specific phobias, OCD, and PTSD). A total of 168 trials with 22,144 patients could be included. Using a unified meta-analytic approach, we found moderate to large effects across indications, ranging from *g* = 0.57 (PTSD) to *g* = 1.18 (specific phobias). For most disorders, both guided and unguided interventions were found to be effective. However, these benefits were not robust across all sensitivity analyses. Overall, positive effects could only be ascertained for three disorders (depression, GAD, and phobias) when analyses were restricted to low risk of bias evidence. A significant superiority against CAU was only found for two conditions (depression and insomnia). Certainty of evidence was rated “low” or “very low” for all but two disorders (SAD and specific phobia). Estimated study dropout rates did not exceed 20 % in most cases. However, differential dropout was common, with higher dropout rates in the intervention arms.

### 4.1 Effectiveness of digital treatments

Our finding that digital interventions can be an effective treatment for common mental disorders is in line with previous meta-analytic evidence (Karyotaki et al., 2021; Kuester et al., 2016; Moshe et al., 2021; Pauley et al., 2023; Simon et al., 2023). A particular strength of our study is that all disorders were analyzed using uniform meta-analytic methods, and after applying the same set of eligibility criteria. This also increases the comparability of our estimates across indications. Overall, the largest effects were found for anxiety disorders (GAD, SAD, panic disorder, phobia; *g* = 0.80 to 1.18) and insomnia (*g* = 0.94). This aligns with meta-analytic evidence for psychotherapies in general (Papola et al., 2022; Papola et al., 2024; de Ponti et al., 2024; van Straten et al., 2018), showing that psychological treatment can be very effective for these conditions, including when provided via digital means. For depression, our effect estimate (*g* = 0.62) is comparable to a range of other meta-analytic studies in which face-to-face psychotherapies were included as well (Cuijpers et al., 2021; Cuijpers et al., 2023a). Previous meta-analyses suggest that digital interventions are often not inferior to face-to-face psychotherapy (Carlbring et al., 2018; Hedman-Lagerlöf et al., 2023; Knutzen et al., 2024; Papola et al., 2023), but our current study cannot directly confirm this, since we did not include head-to-head comparisons in our analyses. For PTSD (*g* = 0.57) and OCD (*g* = 0.68), our overall effect estimates were somewhat lower than those of other meta-analytic studies in which face-to-face treatments were examined as well, including analyses based on the Metapsy databases (Cuijpers et al., 2024a, Cuijpers et al., 2024b; Hoppen et al., 2024; Wang et al., 2024a, Wang et al., 2024b). However, for both conditions, larger point estimates emerged when only guided treatments were considered (PTSD: *g* = 0.67; OCD: *g* = 0.77).

Across indications, our multiple meta-regression model predicted lower benefits when no human guidance is provided (Δ_*g*_ = −0.05), but this difference was small and not significant. This is partly in contrast with previous meta-analytic evidence, which reported significantly lower effects for unguided treatment (Cuijpers et al., 2019; Karyotaki et al., 2021; Moshe et al., 2021; Papola et al., 2023). However, this finding has not emerged consistently in the literature (Furukawa et al., 2021; Pauley et al., 2023). There are several plausible explanations why unguided and guided interventions did not differ more strongly. First, although we adjusted for various study characteristics in our meta-regression, we cannot rule out some residual confounding (i.e., that unguided and guided trials differ systematically in their population, comparators, setting, etc.). Confounding is less likely in trials which directly compare guided and unguided versions of the same intervention. In the three-arm trials discussed in section 3.5, for example, differential effects between guided and unguided formats were considerably larger than predicted by our meta-regression (Δ_*g*_ = −0.27 to −0.48; Krämer et al., 2022; Berger et al., 2011). Another possible explanation is that, according to our definition, unguided interventions could also include (semi-)automatized reminders or encouragement aimed at facilitating adherence. Evidence from a component network meta-analysis of digital depression interventions indicates that human or automatized encouragement alone may already enhance treatment effects (Furukawa et al., 2021). This is in line with our finding that “purely” unguided interventions (i.e., without any reported human or automatized encouragement) showed considerably lower effects. A related explanation could be that we only focused on patients with a diagnosed mental disorder. By design, included trials may have therefore provided some structural support to patients (e.g., contact with a clinician to perform diagnostic interviews, contact with study personnel), even though the intervention itself was unguided.

We also note that, while therapeutic support did not predict significantly larger effects, guided treatments tended to show a more robust evidence base. A significant pooled effect could be ascertained for all disorders when only guided formats were considered, while this was not the case for unguided interventions. For most disorders, the number of available guided intervention trials was also larger. Lastly, we did not find a single disorder for which unguided treatment effects remained significant if only low risk of bias evidence was considered. These findings support various national treatment guidelines (e.g. for depression), in which guided formats are typically recommended as the method of choice (Kendrick et al., 2022; Bundesärztekammer (BÄK) et al., 2022; Malhi et al., 2021). Nevertheless, interventions without human support could still be appealing for future research and practice given their scalability, which may be a crucial asset in low-resource settings (Fu et al., 2020; Karyotaki et al., 2023).

### 4.2 Study dropout

Our analyses also synthesized study dropout rates in digital intervention studies. Overall, we found that dropout was modest, and typically did not exceed 20 % in both treatment and control arms. Dropout rates were also not substantially higher in unguided intervention arms compared to guided treatments. A frequently stated limitation of digital interventions are low adherence rates, whereby patients stop using the intervention after some time (Christensen et al., 2009; Eysenbach, 2005; Fuhr et al., 2018). It is important to stress that our analyses focused on study dropout, which is not identical to treatment discontinuation. However, from a methodological standpoint, study dropout is still highly relevant because it can lead to an upward bias in the “treatment policy estimand” (Kahan et al., 2024; Harrer et al., 2023) that trials typically aim to capture (i.e., the overall effect if the treatment were to be applied under close to real-world conditions). Our results indicate that the risk of such distortions may not be a priori larger in digital intervention trials compared to studies investigating face-to-face treatments, although it should be noted that the two were not directly compared in our analysis. A more concerning finding is that, for most disorders, more patients in the treatment arm dropped out of the study compared to the control group. Such differential dropout can, but does not have to, lead to distortions in the estimated treatment effect (Bell et al., 2013).

### 4.3 Evidence gaps & future research

Our study highlights a substantial increase in trials investigating digital treatments over the past two decades. It should be noted that the 168 studies included in our analysis likely represent only a portion of all digital intervention trials for these conditions. This is because we focused on RCTs enrolling patients with diagnosed mental disorders, and excluded studies that relied on cut-off criteria alone. Although our findings generally support the effectiveness of digital interventions for all studied conditions, some gaps in the evidence became apparent. The first problem is that only a fraction of trials received a low risk of bias rating. The percentage of low risk of bias trials did not exceed 40 % for any disorder, and for one (OCD), no low risk of bias evidence was available at all. While our meta-regression did not indicate a large effect of risk of bias on effect estimates after controlling for confounders, significant effects among low RoB studies could only be ascertained for depression, GAD, and specific phobias. In the future, trialists may put a greater emphasis on design and analysis-related aspects that increase the credibility of their findings. Practical guidance on how to conduct and evaluate a high-quality intervention trial has been developed by our group (Harrer et al., 2023). Another limitation we see in the current evidence is an over-reliance on waitlists, while CAU conditions remain underused. For example, we could only determine a significant effect of digital interventions against CAU conditions for two disorders (depression and insomnia). Waitlists have been frequently shown to overestimate the efficacy of psychological treatment (Cristea, 2019; Cuijpers et al., 2024b; Furukawa et al., 2014), and some have called them a “nocebo” (Furukawa et al., 2014). To better assess the benefits of digital interventions in routine care, treatments need to be compared against a more realistic baseline. In most cases, this will be CAU or some other established form of care.

In this study, we could identify 16 trials in which a prescribable digital therapeutic was evaluated. It should be noted that prescribable DTx have only become available very recently, and that most of these interventions were evaluated before being commercialized under this framework. We may have also missed some trials because interventions were evaluated under a different name, or because manufacturers did not use all available trials to support their evidence claims. However, our findings show that digital interventions now increasingly move from research into routine care, and that for-profit companies drive this development. It remains to be determined what implications this will have on the research landscape on digital interventions. In the future, a more widespread dissemination of regulatory pathways for prescription DTx could lead to a rapid increase in industry-sponsored trials. Such trials will often be designed to obtain regulatory approval, but not necessarily to address existing research gaps. Overall, we believe the commercialization of digital therapeutics presents both promises and challenges. On the one hand, this could lead to greater investment in the dissemination and real-world evaluation of digital interventions, which remains a common barrier (Apolinário-Hagen et al., 2018a; Apolinário-Hagen et al., 2018b; Bennett and Glasgow, 2009; Bührmann et al., 2020; Fleming et al., 2018). On the other hand, greater industry involvement could also lead to sponsorship biases that have previously been more strongly associated with pharmaceutical research (Lundh et al., 2017; Siena et al., 2023; Ebrahim et al., 2016).

### 4.4 Limitations

Our study has several limitations. First, while we provide a “high-level” overview of digital intervention effects, many more granular questions could not be addressed. For example, we did not assess the number of treatment sessions as a potential effect moderator. The number of sessions is more difficult to compare across digital interventions than across conventional face-to-face therapies, especially since we also included smartphone- and virtual reality-based interventions. We also did not conduct a specific analysis focusing on long-term effects of digital treatments, or on effects in LMICs. A last limitation pertains to our identification of prescribable DTx, which was not based on a systematic database search. While some national regulatory agencies (e.g. in Germany) maintain a centralized register of approved DTx, such databases do not exist for other countries with similar regulatory pathways. Available formats and their evidence base therefore had to be reconstructed from published reviews.

## 5 Conclusion

We conclude that digital interventions can be an effective treatment for a wide range of diagnosed mental disorders, yielding moderate to large effect sizes. For some indications, more high-quality evidence is needed to confirm the robustness of our estimated benefits, especially for unguided formats. Study dropout was mostly moderate, but often larger in the digital intervention groups. An increasing number of treatments evaluated in digital intervention RCTs has since become available in routine care as a prescribable digital therapeutic.

## Funding

DP was funded by the European Union’s Horizon-MSCA-2021-PF01 research programme under grant agreement no. 101061648. The funder had no role in the design, preparation, review or approval of the manuscript, or the decision to submit it for publication.

## Declaration of competing interest

TAF reports personal fees from Boehringer-Ingelheim, Daiichi Sankyo, DT Axis, Kyoto University Original, Shionogi, SONY and UpToDate, and a grant from DT Axis and Shionogi, outside the submitted work. In addition, TAF has a patent 7448125, and a pending patent 2022-082495, and intellectual properties for Kokoro-app licensed to Mitsubishi-Tanabe. In the last three years, SL has received honoraria for advising/consulting and/or for lectures and/or for educational material from Angelini, Apsen, Boehringer Ingelheim, Eisai, Ekademia, GedeonRichter, Janssen, Karuna, Kynexis, Lundbeck, Medichem, Medscape, Mitsubishi, Neurotorium, Otsuka, NovoNordisk, Recordati, Rovi, Teva. All the other authors have no conflict of interest to declare.

## Appendix A Supplementary data

Supplementary material Image 1
