# Supplementary material for: Effectiveness of digital interventions for eight mental disorders: A meta-analytic synthesis
Source: Internet Interv. 2025 Jul 11;41:100860. doi: 10.1016/j.invent.2025.100860 (PMC12284662; doi:10.1016/j.invent.2025.100860)
Supplement: Supplementary file 1 — Supplementary material [file mmc1.pdf]

Effectiveness of Digital Interventions for Eight Mental Disorders: A Meta-Analytic Synthesis

Supplement

Table of Contents

S1. *Search strings.* 2
- Depression 2
- Anxiety Disorders 5
- Posttraumatic Stress Disorder 9
- Obsessive-Compulsive Disorder 11
- Insomnia 12

S2. *Definition & identification of prescription digital therapeutics.* 13

S3. *PRISMA flowcharts.* 14
- Depression 14
- Anxiety Disorders 14
- Posttraumatic Stress Disorder 15
- Obsessive-Compulsive Disorder 15
- Insomnia 16

S4. *References of the included studies.* 17
- Depression 17
- Insomnia 19
- Social Anxiety Disorder 21
- Panic Disorder 23
- Posttraumatic Stress Disorder 24
- Generalized Anxiety Disorder 25
- Obsessive-Compulsive Disorder 26
- Specific Phobia 27

S5. *Effects of digital interventions for eight mental disorders (sensitivity analyses)* 28

S6. *Effects of purely unguided interventions* 32

S7. *Dropout rates per arm and disorder.* 33

S8. *Differential dropout rates per disorder.* 34

S9. *Results of moderator analyses across disorders (variable-by-variable).* 35

S10. *Results of GRADE assessment.* 36

S11. *Number of digital intervention trials per country.* 37

S1. *Search strings.*

Depression

PubMed:
Psychotherapy [MH] OR psychotherap*[All Fields] OR cbt[All Fields] OR "behavior therapies"[All Fields] OR "behavior therapy"[All Fields] OR "behavior therapeutic"[All Fields] OR "behavior therapeutical"[All Fields] OR "behavior therapeutics"[All Fields] OR "behavior therapeutist"[all Fields] OR "behavior therapeutists"[All Fields] OR "behavior treatment"[All Fields] OR "behavior treatments"[All Fields] OR "behaviors therapies"[All Fields] OR "behaviors therapy"[All Fields] OR "behaviors therapeutics"[All Fields] OR "behaviors therapeutic"[All Fields] OR "behaviors therapeutical"[All Fields] OR "behaviors therapeutist"[All Fields] OR "behaviors therapeutists"[All Fields] OR "behaviors treatment"[All Fields] OR "behaviors treatments"[All Fields] OR "behavioral therapies"[All Fields] OR "behavioral therapy"[All Fields] OR "behavioral therapeutics"[All Fields] OR "behavioral therapeutic"[All Fields] OR "behavioral therapeutical"[All Fields] OR "behavioral therapeutist"[All Fields] OR "behavioral therapeutists"[All Fields] OR "behavioral treatment"[All Fields] OR "behavioral treatments"[All Fields] OR "behaviour therapies"[All Fields] OR "behaviour therapy"[All Fields] OR "behaviour therapeutic"[All Fields] OR "behaviour therapeutical"[All Fields] OR "behaviour therapeutics"[All Fields] OR "behaviour therapeutist"[all Fields] OR "behaviour therapeutists"[All Fields] OR "behaviour treatment"[All Fields] OR "behaviour treatments"[All Fields] OR "behaviours therapies"[All Fields] OR "behaviours therapy"[All Fields] OR "behaviours therapeutics"[All Fields] OR "behaviours therapeutic"[All Fields] OR "behaviours therapeutical"[All Fields] OR "behaviours therapeutist"[All Fields] OR "behaviours therapeutists"[All Fields] OR "behaviours treatment"[All Fields] OR "behaviours treatments"[All Fields] OR "behavioural therapies"[All Fields] OR "behavioural therapy"[All Fields] OR "behavioural therapeutics"[All Fields] OR "behavioural therapeutic"[All Fields] OR "behavioural therapeutical"[All Fields] OR "behavioural therapeutist"[All Fields] OR "behavioural therapeutists"[All Fields] OR "behavioural treatment"[All Fields] OR "behavioural treatments"[All Fields] OR "cognition therapies"[All Fields] OR "cognition therapie"[All Fields] OR "cognition therapy"[All Fields] OR "cognition therapeutical"[All Fields] OR "cognition therapeutic"[All Fields] OR "cognition therapeutics"[All Fields] OR "cognition therapeutist"[All Fields] OR "cognition therapeutists"[All Fields] OR "cognition treatment"[All Fields] OR "cognition treatments"[All Fields] OR psychodynamic[All Fields] OR Psychoanalysis[MH] OR psychoanalysis[All Fields] OR psychoanalytic*[All Fields] OR counselling[All Fields] OR counseling[All Fields] OR Counseling[MH] OR "problem-solving"[All Fields] OR mindfulness[All Fields] OR (acceptance[All Fields] AND commitment[All Fields]) OR "assertiveness training"[All Fields] OR "behavior activation"[All Fields] OR "behaviors activation"[All Fields] OR "behavioral activation"[All Fields] OR "cognitive therapies"[All Fields] OR "cognitive therapy"[All Fields] OR "cognitive therapeutic"[All Fields] OR "cognitive therapeutics"[All Fields] OR "cognitive therapeutical"[All Fields] OR "cognitive therapeutist"[All Fields] OR "cognitive therapeutists"[All Fields] OR "cognitive treatment"[All Fields] OR "cognitive treatments"[All Fields] OR "cognitive restructuring"[All Fields] OR (("compassion-focused"[All Fields] OR "compassion-focussed"[All Fields]) AND (therapy[SH] OR therapies[All Fields] OR therapy[All Fields] OR therape*[All Fields] OR therapis*[All Fields]OR Therapeutics [OR treatment*[All Fields])) OR ((therapy[SH] OR therapies[All Fields] OR therapy [All Fields] OR therape*[All Fields] OR therapis*[All Fields] OR Therapeutics[MH] OR treatment*[All Fields]) AND constructivist*[All Fields]) OR "metacognitive therapies"[All Fields] OR "metacognitive therapy"[All Fields] OR "metacognitive therapeutic"[All Fields] OR "metacognitive therapeutics"[All Fields] OR "metacognitive therapeutical"[All Fields] OR "metacognitive therapeutist"[All Fields] OR "metacognitive therapeutists"[All Fields] OR "metacognitive treatment"[All Fields] OR "metacognitive treatments"[All Fields] OR "meta-cognitive therapies"[All Fields] OR "meta-cognitive therapy"[All Fields] OR "meta-cognitive therapeutic"[All Fields] OR "meta-cognitive therapeutics"[All Fields] OR "meta-cognitive therapeutical"[All Fields] OR "meta-cognitive therapeutist"[All Fields] OR "meta-cognitive therapeutists"[All Fields] OR "meta-cognitive treatment"[All Fields] OR "meta-cognitive treatments"[All Fields] OR "solution-focused therapies"[All Fields] OR "solution-focused therapy"[All Fields] OR "solution-focused therapeutic"[All Fields] OR "solution-focused therapeutics"[All Fields] OR "solution-focused therapeutical"[All Fields] OR "solution focused therapies"[All Fields] OR "solution focused therapy"[All Fields] OR "solution focused therapeutic"[All Fields] OR "solution focused therapeutics"[All Fields] OR "solution focused therapeutical"[All Fields]OR "solution-focussed therapies"[All Fields] OR "solution-focussed therapy"[All Fields] OR "solution-focussed therapeutic"[All Fields] OR "solution-focussed therapeutics"[All Fields] OR "solution-focussed therapeutical"[All Fields]OR "solution focussed therapies"[All Fields] OR "solution focussed therapy"[All Fields] OR "solution focussed therapeutic"[All Fields] OR "solution focussed therapeutics"[All Fields] OR "solution focussed therapeutical"[All Fields] OR "self-control therapies"[All Fields] OR "self-control therapy"[All Fields] OR "self-control therapeutics"[All Fields] OR "self-control therapeutical"[All Fields] OR "self-control therapeutic"[All Fields] OR "self- control training"[All Fields] OR "self-control trainings"[All Fields] OR "self control therapies"[All Fields] OR "self control therapy"[All Fields] OR "self control therapeutics"[All Fields] OR "self control therapeutical"[All Fields] OR "self control therapeutic"[All Fields] OR "self control training"[All Fields] OR "self control trainings"[All Fields]
AND
(Depressive Disorder[MH] OR Depression[MH]OR dysthymi*[All Fields] OR "affective disorder"[All Fields]OR "affective disorders"[All Fields] OR "mood disorder"[All Fields] OR "mood disorders"[All Fields] OR depression*[All Fields] OR depressive*[All Fields] OR "dysthymic disorder"[MeSH Terms])
Limits: RCTs

Embase:
#1 'psychotherapy'/exp OR 'psychotherapy' OR 'psychotherapies' OR 'psychotherapeutics' OR 'psychotherapeutical' OR 'cognitive therapy'/exp OR 'cognitive behavior therapy'/exp OR 'behavior therapy'/exp OR´cbt´ OR´cognitive behavioural therapy´ OR´cognitive behavioural therapies´ OR cognitive behavioral therapy´ OR 'cognitive behavioral therapies' OR 'behavior therapy' OR 'behavior therapies' OR 'behaviour therapy' OR 'behaviour therapies' OR 'cognition therapy' OR 'cognitive therapies' OR 'cognitive therapy' OR 'cognitive therapeutic' OR 'cognitive therapeutics' OR 'cognitive therapeutical' OR 'cognitive therapeutist' OR 'cognitive therapeutists' OR 'cognitive treatment' OR 'cognitive treatments' OR 'cognitive restructuring' OR 'cognition therapies' OR 'cognition therapie' OR 'cognition therapeutical' OR 'cognition therapeutic' OR 'cognition therapeutics' OR 'cognition therapeutist' OR 'cognition therapeutists' OR 'cognition treatment' OR 'cognition treatments' OR 'behavior therapeutic' OR 'behavior therapeutical' OR 'behavior therapeutics' OR 'behavior therapeutist' OR 'behavior therapeutists' OR 'behavior treatment' OR 'behavior treatments' OR 'behaviors therapies' OR 'behaviors therapy' OR 'behaviors therapeutics' OR 'behaviors therapeutic' OR 'behaviors therapeutical' OR 'behaviors therapeutist' OR 'behaviors therapeutists' OR 'behaviors treatment' OR 'behaviors treatments' OR 'behavioral therapies' OR 'behavioral therapy' OR 'behavioral therapeutics' OR 'behavioral therapeutic' OR 'behavioral therapeutical' OR 'behavioral therapeutist' OR 'behavioral therapeutists' OR 'behavioral treatment' OR 'behavioral treatments' OR 'behaviour therapeutic' OR 'behaviour therapeutical' OR 'behaviour therapeutics' OR 'behaviour therapeutist' OR 'behaviour therapeutists' OR 'behaviour treatment' OR 'behaviour treatments' OR 'behaviours therapies' OR 'behaviours therapy' OR 'behaviours therapeutics' OR 'behaviours therapeutic' OR 'behaviours

therapeutical' OR 'behaviours therapeutist' OR 'behaviours therapeutists' OR 'behaviours treatment' OR 'behaviours treatments' OR 'behavioural therapies' OR 'behavioural therapy' OR 'behavioural therapeutics' OR 'behavioural therapeutic' OR 'behavioural therapeutical' OR 'behavioural therapeutist' OR 'behavioural therapeutists' OR 'behavioural treatment' OR 'behavioural treatments' OR 'behavior activation' OR 'behaviors activation' OR 'behavioral activation' OR 'behaviour activation' OR 'behaviours activation' OR 'behavioural activation' OR 'psychoanalytic therapy'/exp OR 'psychodynamic' OR 'psychodynamical' OR 'psychoanalysis' OR 'psychoanalytical' OR 'counselling'/exp OR 'counseling'/exp OR 'counselling' OR 'counseling' OR 'problem-solving' OR 'problem solving' OR 'supportive therapy' OR 'metacognitive therapy' OR 'metacognitive therapies' OR 'metacognitive therapeutic' OR 'metacognitive therapeutics' OR 'metacognitive therapeutical' OR 'metacognitive therapeutist' OR 'metacognitive therapeutists' OR 'metacognitive treatment' OR 'metacognitive treatments' OR 'meta-cognitive therapy' OR 'meta-cognitive therapies' OR 'meta-cognitive therapeutic' OR 'meta-cognitive therapeutics' OR 'meta-cognitive therapeutical' OR 'meta-cognitive therapeutist' OR 'meta-cognitive therapeutists' OR 'meta-cognitive treatment' OR 'meta-cognitive treatments' OR 'solution-focused therapies' OR 'solution focused therapies' OR 'solution-focussed therapies' OR 'solution focused therapies' OR 'solution- focused therapy' OR 'solution focused therapy' OR 'solution-focussed therapy' OR 'solution focused therapy' OR 'solution-focused therapeutic' OR 'solution focused therapeutic' OR 'solution-focussed therapeutic' OR 'solution focussed therapeutic' OR 'solution-focused therapeutics' OR 'solution focused therapeutics' OR 'solution-focussed therapeutics' OR 'solution focused therapeutics' OR 'solution-focused therapeutical' OR 'solution focused therapeutical' OR 'solution-focussed therapeutical' OR 'solution focused therapeutical' OR 'self-control therapies' OR 'self control therapies' OR 'self-control therapy' OR 'self control therapy' OR 'self-control therapeutics' OR 'self control therapeutics' OR 'self-control therapeutical' OR 'self control therapeutical' OR 'self-control therapeutic' OR 'self control therapeutic' OR 'self-control training' OR 'self control training' OR 'self control trainings' OR 'self-control trainings' OR 'mindfulness' OR 'acceptance commitment' OR 'acceptance and commitment' OR 'assertiveness training'
#2 'compassion-focused' OR 'compassion-focussed' OR 'compassion focused' OR 'compassion focussed' OR 'constructivist' OR 'constructivists'
#3 'therapies' OR 'therapy' OR 'therapeutics' OR 'therapist' OR 'treatment' OR 'treatments'
#4 Combine: #2 AND #3
#5: #1 OR #4
#6 'depressive disorder'/exp OR 'depression'/exp OR 'depressive' OR 'major depression'/exp OR 'major depressive disorder'/exp OR 'depression' OR 'depressions' OR 'depressive' OR 'dysthymic disorder'/exp OR 'dysthymic disorder' OR 'dysthymia'/exp OR 'dysthymic' OR 'mood disorder'/exp OR 'affective disorder'/exp OR 'affective disorder' OR 'affective disorders' OR 'mood disorder' OR 'mood disorders'
Combine: #5 AND #6
Limits: RCTs

PsycINFO:
(DE "Psychotherapy" OR "Psychotherapy" OR "psychotherapies" OR "psychotherapeutic" OR "psychotherapeutical" OR "psychotherapeutics" OR DE "Behavior Therapy" OR DE "Cognitive Behavior Therapy" OR "CBT" OR "behavior therapies" OR "behavior therapy" OR "behavior therapeutic" OR "behavior therapeutical" OR "behavior therapeutics"
OR "behavior therapeutist" OR "behavior therapeutists" OR "behavior treatment" OR "behavior treatments" OR "behaviors therapies" OR "behaviors therapy" OR "behaviors therapeutics" OR "behaviors therapeutic" OR "behaviors therapeutical" OR "behaviors therapeutist" OR "behaviors therapeutists" OR "behaviors treatment" OR "behaviors treatments" OR "behavioral therapies" OR "behavioral therapy" OR "behavioral therapeutics" OR "behavioral therapeutic" OR "behavioral therapeutical" OR "behavioral therapeutist" OR "behavioral therapeutists" OR "behavioral treatment" OR "behavioral treatments" OR "behaviour therapies" OR "behaviour therapy" OR "behaviour therapeutic" OR "behaviour therapeutical" OR "behaviour therapeutics" OR "behaviour therapeutist" OR "behaviour therapeutists" OR "behaviour treatment" OR "behaviour treatments" OR "behaviours therapies" OR "behaviours therapy" OR "behaviours therapeutics" OR "behaviours therapeutic" OR "behaviours therapeutical" OR "behaviours therapeutist" OR "behaviours therapeutists" OR "behaviours treatment" OR "behaviours treatments" OR "behavioural therapies" OR "behavioural therapy" OR "behavioural therapeutics" OR "behavioural therapeutic" OR "behavioural therapeutical" OR "behavioural therapeutist" OR "behavioural therapeutists" OR "behavioural treatment" OR "behavioural treatments" OR "cognition therapies" OR "cognition therapie" OR "cognition therapy" OR "cognition therapeutical" OR "cognition therapeutic" OR "cognition therapeutics" OR "cognition therapeutist" OR "cognition therapeutists" OR "cognition treatment" OR "cognition treatments" OR "cognitive therapies" OR "cognitive therapy" OR "cognitive therapeutic" OR "cognitive therapeutics" OR "cognitive therapeutical" OR "cognitive therapeutist" OR "cognitive therapeutists" OR "cognitive treatment" OR "cognitive treatments" OR "cognitive restructuring" OR DE "Emotion Focused Therapy" OR DE "Psychoanalysis" OR "psychoanalysis" OR "psychoanalytic" OR "psychoanalytical "OR DE "Psychodynamic Psychotherapy" OR "psychodynamic" OR DE "Psychotherapeutic Counseling" OR "counselling" OR "counseling" OR "problem-solving" OR "problem solving" OR "mindfulness" OR ("acceptance" AND "commitment") OR "assertiveness training" OR "behavior activation" OR "behaviors activation" OR "behavioral activation" OR "behaviour activation" OR "behaviours activation" OR "behavioural activation" OR "metacognitive therapies" OR "metacognitive therapy" OR "metacognitive therapeutic" OR "metacognitive therapeutics" OR "metacognitive therapeutical" OR "metacognitive therapeutist" OR "metacognitive therapeutists" OR "metacognitive treatment" OR "metacognitive treatments" OR "meta-cognitive therapies" OR "meta-cognitive therapy" OR "meta-cognitive therapeutic" OR "meta-cognitive therapeutics" OR "meta-cognitive therapeutical" OR "meta-cognitive therapeutist" OR "meta-cognitive therapeutists" OR "meta-cognitive treatment" OR "meta-cognitive treatments" OR DE "Solution Focused Therapy" OR "solution- focused therapies" OR "solution-focused therapy" OR "solution-focused therapeutic" OR "solution-focused therapeutics" OR "solution-focused therapeutical" OR "solution-focussed therapies" OR "solution-focussed therapy" OR "solution-focussed therapeutic" OR "solution-focussed therapeutics" OR "solution-focussed therapeutical" OR "solution focused therapies" OR "solution focused therapy" OR "solution focused therapeutic" OR "solution focused therapeutics" OR "solution focused therapeutical" OR "solution focussed therapies" OR "solution focussed therapy" OR "solution focussed therapeutic" OR "solution focussed therapeutics" OR "solution focussed therapeutical" OR "self- control therapies" OR "self-control therapy" OR "self-control therapeutics" OR "self-control therapeutical" OR "self- control therapeutic" OR "self-control training" OR "self-control trainings" OR "self control therapies" OR "self control therapy" OR "self control therapeutics" OR "self control therapeutical" OR "self control therapeutic" OR "self control training" OR "self control trainings" OR (("compassion-focused" OR "compassion-focussed" OR "compassion focused" OR "compassion focussed") AND ("therapies" OR "therapy" OR "therapie" OR "therapist" OR "therapists" OR "therapeut" OR "treatment" OR "treatments")) OR ("constructivist" AND ("therapies" OR "therapy" OR "therapie" OR "therapist" OR "therapists" OR "therapeut" OR "treatment" OR "treatments")))
AND
(DE "Depression (Emotion)" "depressive disorder" OR "depression" OR "depressions" OR "depressive" OR DE "Major Depression" OR "major depression" OR "major depressive disorder" OR DE "Dysthymic Disorder" OR "Dysthymia" OR " dysthymic disorder" OR DE "Affective Disorders" OR "Affective Disorder" OR "affective disorders" OR "Mood Disorder" OR "Mood disorders")
Limits: Methodology is ME=(treatment outcome/clinical trial): papers

Cochrane:

#1 MeSH descriptor: [Depressive Disorder] explode all trees : 6, 777
#2 ''depress*'' (Word variations have been searched) : 51, 768
#3 #1or#2 :51,783
#4 ''major depressive disorder'' (Word variations have been searched) : 5, 435
#5 #3or#4 :51,783
#6 MeSH descriptor: [Dysthymic Disorder] explode all trees : 129
#7 ''dysthymi*'' (Word variations have been searched) : 649
#8 #6or#7 :649
#9 #5or#8 :51,800
#10 ''mood disorder'' (Word variations have been searched) :4, 034
#11 ''affective disorder'' (Word variations have been searched) : 2, 882
#12 #10or#11:6,055
#13 #9or#12:53,227
#14 MeSH descriptor: [Psychotherapy] explode all trees : 13, 568
#15 ''psychotherap*'' (Word variations have been searched) : 7, 758
#16 ''CBT'' (Word variations have been searched) : 2, 029
#17 ''Cognitive Behav* therap* (Word variations have been searched) : 8, 893
#18 #14or#15or#16or#17 :20,795
#19 'psychodynamic'' (Word variations have been searched) : 469
#20 MeSH descriptor: [Psychoanalysis] explode all trees : 13
#21 ''psychoanaly*'' (Word variations have been searched) : 345
#22 MeSH descriptor: [Counseling] explode all trees : 2, 783
#23 ''counseling*'' (Word variations have been searched) : 6, 913
#24 ''problem solving'' (Word variations have been searched) : 2, 867
#25 #18or#19or#20or#21or#22or#23or#24 :28,149
#26 ''acceptance commitment'' (Word variations have been searched) : 168
#27 ''assertiveness training'' (Word variations have been searched) :231
#28 ''behavior activation'' (Word variations have been searched) : 663
#29 ''mindfulness'' (Word variations have been searched) : 466
#30 ''metacognitive therap*'' (Word variations have been searched) :56
#31 ''solution focused therap*'' (Word variations have been searched) :858
#32 ''self control training'' (Word variations have been searched): 5850
#33 #25or#26or#27or#28or#29or#30or#31or#32 :32,748
#34 ''Randomized Controlled Trial'':ti,ab,kw (Word variations have been searched) : 120, 901
#35 #13 and #33 and #34 in Trials

Anxiety Disorders

A comprehensive anxiety literature database was used as the information source of the present review and has been registered at the open science framework (https://osf.io/9xe2g/). Development of the database began with a systematic search on 25 April 2019, and it is kept update on every January by two independent researchers. A systematic search was conducted using a full range of terms related to the applicable interventions, disorders and outcomes. Similar databases exist for depression[1], treatments of suicide[2], post-traumatic stress disorder[3] and mental health problems in children and adolescents[4]. Each of these includes several hundreds of randomized trials. We have recently named such living systematic reviews focusing on a specific research area as 'Meta-Analytic Research Domains' (MARDs)[5]. From our anxiety MARD we selected trials on generalized anxiety disorder, panic disorder, social anxiety disorder, and specific phobias.

[1]Cuijpers P. Four decades of outcome research on psychotherapies for adult depression: an overview of a series of meta-analyses. Can Psychol 2017;58:7–19; [2]Hu MX, Palantza C, Setkowski K, Gilissen R, Karyotaki E, Cuijpers P, Riper H, de Beurs D, Nuij C, Christensen H, Calear A, Werner-Seidler A, Hoogendoorn A, van Balkom A, Eikelenboom M, Smit J, van Ballegooijen W. Comprehensive database and individual patient data meta-analysis of randomised controlled trials on psychotherapies reducing suicidal thoughts and behaviour: study protocol. BMJ Open. 2020 Dec 4;10(12):e037566; [3]U.S. Department of Veterans Affairs. National center for PTSD, 2022. Available: www.ptsd.va.gov/ ptsdrepository/index.asp; [4]Weisz JR, Kuppens S, Ng MY, Eckshtain D, Ugueto AM, Vaughn-Coaxum R, Jensen-Doss A, Hawley KM, Krumholz Marchette LS, Chu BC, Weersing VR, Fordwood SR. What five decades of research tells us about the effects of youth psychological therapy: A multilevel meta-analysis and implications for science and practice. Am Psychol. 2017 Feb-Mar;72(2):79-117; [5]Cuijpers P, Miguel C, Papola D, Harrer M, Karyotaki E. From living systematic reviews to meta-analytical research domains. Evid Based Ment Health. 2022 Jul 19:ebmental-2022-300509.

PubMed:
Psychotherapy[MH] OR psychotherap*[All Fields] OR cbt[All Fields] OR "behavior therapies"[All Fields] OR "behavior therapy"[All Fields] OR "behavior therapeutic"[All Fields] OR "behavior therapeutical"[All Fields] OR "behavior therapeutics"[All Fields] OR "behavior therapeutist"[all Fields] OR "behavior therapeutists"[All Fields] OR "behavior treatment"[All Fields] OR "behavior treatments"[All Fields] OR "behaviors therapies"[All Fields] OR "behaviors therapy"[All Fields] OR "behaviors therapeutics"[All Fields] OR "behaviors therapeutic"[All Fields] OR "behaviors therapeutical"[All Fields] OR "behaviors therapeutist"[All Fields] OR "behaviors therapeutists"[All Fields] OR "behaviors treatment"[All Fields] OR "behaviors treatments"[All Fields] OR "behavioral therapies"[All Fields] OR "behavioral therapy"[All Fields] OR "behavioral therapeutics"[All Fields] OR "behavioral therapeutic"[All Fields] OR "behavioral therapeutical"[All Fields] OR "behavioral therapeutist"[All Fields] OR "behavioral therapeutists"[All Fields] OR "behavioral treatment"[All Fields] OR "behavioral treatments"[All Fields] OR "behaviour therapies"[All Fields] OR "behaviour therapy"[All Fields] OR "behaviour therapeutic"[All Fields] OR "behaviour therapeutical"[All Fields] OR "behaviour therapeutics"[All Fields] OR "behaviour therapeutist"[all Fields] OR "behaviour therapeutists"[All Fields] OR "behaviour treatment"[All Fields] OR "behaviour treatments"[All Fields] OR "behaviours therapies"[All Fields] OR "behaviours therapy"[All Fields] OR "behaviours therapeutics"[All Fields] OR "behaviours therapeutic"[All Fields] OR "behaviours therapeutical"[All Fields] OR "behaviours therapeutist"[All Fields] OR "behaviours therapeutists"[All Fields] OR "behaviours treatment"[All Fields] OR "behaviours treatments"[All Fields] OR "behavioural therapies"[All Fields] OR "behavioural therapy"[All Fields] OR "behavioural therapeutics"[All Fields] OR "behavioural therapeutic"[All Fields] OR "behavioural therapeutical"[All Fields] OR "behavioural therapeutist"[All Fields] OR "behavioural therapeutists"[All Fields] OR "behavioural treatment"[All Fields] OR "behavioural treatments"[All Fields] OR "cognition therapies"[All Fields] OR "cognition therapie"[All Fields] OR "cognition therapy"[All Fields] OR "cognition therapeutical"[All Fields] OR "cognition therapeutic"[All Fields] OR "cognition therapeutics"[All Fields] OR "cognition therapeutist"[All Fields] OR "cognition therapeutists"[All Fields] OR "cognition treatment"[All Fields] OR "cognition treatments"[All Fields] OR psychodynamic[All Fields] OR Psychoanalysis[MH] OR psychoanalysis[All Fields] OR psychoanalytic*[All Fields] OR counselling[All Fields] OR counseling[All Fields] OR Counseling[MH] OR "problem-solving"[All Fields] OR mindfulness[All Fields] OR (acceptance[All Fields] AND commitment[All Fields]) OR "assertiveness training"[All Fields] OR "behavior activation"[All Fields] OR "behaviors activation"[All Fields] OR "behavioral activation"[All Fields] OR "cognitive therapies"[All Fields] OR "cognitive therapy"[All Fields] OR "cognitive therapeutic"[All Fields] OR "cognitive therapeutics"[All Fields] OR "cognitive therapeutical"[All Fields] OR "cognitive therapeutist"[All Fields] OR "cognitive therapeutists"[All Fields] OR "cognitive treatment"[All Fields] OR "cognitive treatments"[All Fields] OR "cognitive restructuring"[All Fields] OR (("compassion-focused"[All Fields] OR "compassion-focussed"[All Fields]) AND (therapy[SH] OR therapies[All Fields] OR therapy[All Fields] OR therape*[All Fields] OR therapis*[All Fields]OR Therapeutics [OR treatment*[All Fields])) OR ((therapy[SH] OR therapies[All Fields] OR therapy [All Fields] OR therape*[All Fields] OR therapis*[All Fields] OR Therapeutics[MH] OR treatment*[All Fields]) AND constructivist*[All Fields]) OR "metacognitive therapies"[All Fields] OR "metacognitive therapy"[All Fields] OR "metacognitive therapeutic"[All Fields] OR "metacognitive therapeutics"[All Fields] OR "metacognitive therapeutical"[All Fields] OR "metacognitive therapeutist"[All Fields] OR "metacognitive therapeutists"[All Fields] OR "metacognitive treatment"[All Fields] OR "metacognitive treatments"[All Fields] OR "meta-cognitive therapies"[All Fields] OR "meta-cognitive therapy"[All Fields] OR "meta-cognitive therapeutic"[All Fields] OR "meta-cognitive therapeutics"[All Fields] OR "meta-cognitive therapeutical"[All Fields] OR "meta-cognitive therapeutist"[All Fields] OR "meta-cognitive therapeutists"[All Fields] OR "meta-cognitive treatment"[All Fields] OR "meta-cognitive treatments"[All Fields] OR "solution-focused therapies"[All Fields] OR "solution-focused therapy"[All Fields] OR "solution-focused therapeutic"[All Fields] OR "solution-focused therapeutics"[All Fields] OR "solution-focused therapeutical"[All Fields] OR "solution focused therapies"[All Fields] OR "solution focused therapy"[All Fields] OR "solution focused therapeutic"[All Fields] OR "solution focused therapeutics"[All Fields] OR "solution focused therapeutical"[All Fields]OR "solution-focussed therapies"[All Fields] OR "solution-focussed therapy"[All Fields] OR "solution-focussed therapeutic"[All Fields] OR "solution-focussed therapeutics"[All Fields] OR "solution-focussed therapeutical"[All Fields]OR "solution focussed therapies"[All Fields] OR "solution focussed therapy"[All Fields] OR "solution focussed therapeutic"[All Fields] OR "solution focussed therapeutics"[All Fields] OR "solution focussed therapeutical"[All Fields] OR "self-control therapies"[All Fields] OR "self-control therapy"[All Fields] OR "self-control therapeutics"[All Fields] OR "self-control therapeutical"[All Fields] OR "self-control therapeutic"[All Fields] OR "self-control training"[All Fields] OR "self-control trainings"[All Fields] OR "self control therapies"[All Fields] OR "self control therapy"[All Fields] OR "self control therapeutics"[All Fields] OR "self control

therapeutical"[All Fields] OR "self control therapeutic"[All Fields] OR "self control training"[All Fields] OR "self control trainings"[All Fields] OR exposure[All Fields] OR relaxation[All Fields] OR EMDR[All Fields] OR ("eye movement" and desensiti*[All Fields]) OR "panic management"[All Fields] OR "response prevention"[All Fields] OR ERP[All Fields]
AND
"social anxiety"[All Fields] OR shy[All Fields] OR ("shyness"[MeSH Terms] OR "shyness"[All Fields]) OR "test anxiety"[All Fields] OR gad[All Fields] OR "generalized anxiety"[All Fields] OR "generalised anxiety"[All Fields] OR worry[All Fields] OR ("panic"[MeSH Terms] OR "panic"[All Fields]) OR (agoraphobi[All Fields] OR agoraphobia[All Fields] OR agoraphobia'[All Fields] OR agoraphobia's[All Fields] OR agoraphobias[All Fields] OR agoraphobic[All Fields] OR agoraphobic's[All Fields] OR agoraphobics[All Fields] OR agoraphobics'[All Fields] OR agoraphobie[All Fields] OR agoraphobien[All Fields]) OR "anxiety disorder"[All Fields] OR "social phobia"[All Fields] OR "social anxiety disorder"[All Fields] OR Arachnophobia[All Fields] OR Ophidiophobia[All Fields] OR Acrophobia[All Fields] OR Agoraphobia[All Fields] OR Cynophobia[All Fields] OR Claustrophobia[All Fields] OR Mysophobia[All Fields] OR Aerophobia[All Fields] OR Trypophobia[All Fields] OR Carcinophobia[All Fields] OR Thanatophobia[All Fields] OR Glossophobia[All Fields] OR Monophobia[All Fields] OR Ornithophobia[All Fields] OR Alektorophobia[All Fields] OR Trypanophobia[All Fields] OR Anthropophobia[All Fields] OR Aquaphobia[All Fields] OR Autophobia[All Fields] OR Hemophobia[All Fields] OR Xenophobia[All Fields] OR Ailurophobia[All Fields] OR Nyctophobia[All Fields] OR Phobophobia[All Fields] OR Philophobia[All Fields] OR Triskaidekaphobia[All Fields] OR Emetophobia[All Fields] OR Entomophobia[All Fields] OR Zoophobia[All Fields] OR Scelerophobia[All Fields] OR Cibophobia[All Fields] OR Tokophobia[All Fields] OR Pseudodysphagia[All Fields] OR Gerascophobia[All Fields] OR Technophobia[All Fields] OR Ergophobia[All Fields] OR Coulrophobia [All Fields] OR Photophobia[All Fields] OR Numerophobia[All Fields] OR Taphophobia

PsycINFO:
DE "Psychotherapy" OR "Psychotherapy" OR "psychotherapies" OR "psychotherapeutic" OR "psychotherapeutical" OR "psychotherapeutics" OR DE "Behavior Therapy" OR DE "Cognitive Behavior Therapy" OR "CBT" OR "behavior therapies" OR "behavior therapy" OR "behavior therapeutic" OR "behavior therapeutical" OR "behavior therapeutics" OR "behavior therapeutist" OR "behavior therapeutists" OR "behavior treatment" OR "behavior treatments" OR "behaviors therapies" OR "behaviors therapy" OR "behaviors therapeutics" OR "behaviors therapeutic" OR "behaviors therapeutical" OR "behaviors therapeutist" OR "behaviors therapeutists" OR "behaviors treatment" OR "behaviors treatments" OR "behavioral therapies" OR "behavioral therapy" OR "behavioral therapeutics" OR "behavioral therapeutic" OR "behavioral therapeutical" OR "behavioral therapeutist" OR "behavioral therapeutists" OR "behavioral treatment" OR "behavioral treatments" OR "behaviour therapies" OR "behaviour therapy" OR "behaviour therapeutic" OR "behaviour therapeutical" OR "behaviour therapeutics" OR "behaviour therapeutist" OR "behaviour therapeutists" OR "behaviour treatment" OR "behaviour treatments" OR "behaviours therapies" OR "behaviours therapy" OR "behaviours therapeutics" OR "behaviours therapeutic" OR "behaviours therapeutical" OR "behaviours therapeutist" OR "behaviours therapeutists" OR "behaviours treatment" OR "behaviours treatments" OR "behavioural therapies" OR "behavioural therapy" OR "behavioural therapeutics" OR "behavioural therapeutic" OR "behavioural therapeutical" OR "behavioural therapeutist" OR "behavioural therapeutists" OR "behavioural treatment" OR "behavioural treatments" OR "cognition therapies" OR "cognition therapie" OR "cognition therapy" OR "cognition therapeutical" OR "cognition therapeutic" OR "cognition therapeutics" OR "cognition therapeutist" OR "cognition therapeutists" OR "cognition treatment" OR "cognition treatments" OR "cognitive therapies" OR "cognitive therapy" OR "cognitive therapeutic" OR "cognitive therapeutics" OR "cognitive therapeutical" OR "cognitive therapeutist" OR "cognitive therapeutists" OR "cognitive treatment" OR "cognitive treatments" OR "cognitive restructuring" OR DE "Emotion Focused Therapy" OR DE "Psychoanalysis" OR "psychoanalysis" OR "psychoanalytic" OR "psychoanalytical "OR DE "Psychodynamic Psychotherapy" OR "psychodynamic" OR DE "Psychotherapeutic Counseling" OR "counselling" OR "counseling" OR "problem-solving" OR "problem solving" OR "mindfulness" OR "acceptance and commitment" OR "assertiveness training" OR "behavior activation" OR "behaviors activation" OR "behavioral activation" OR "behaviour activation" OR "behaviours activation" OR "behavioural activation" OR "metacognitive therapies" OR "metacognitive therapy" OR "metacognitive therapeutic" OR "metacognitive therapeutics" OR "metacognitive therapeutical" OR "metacognitive therapeutist" OR "metacognitive therapeutists" OR "metacognitive treatment" OR "metacognitive treatments" OR "meta-cognitive therapies" OR "meta-cognitive therapy" OR "meta-cognitive therapeutic" OR "meta-cognitive therapeutics" OR "meta-cognitive therapeutical" OR "meta-cognitive therapeutist" OR "meta-cognitive therapeutists" OR "meta-cognitive treatment" OR "meta-cognitive treatments" OR DE "Solution Focused Therapy" OR "solution-focused therapies" OR "solution-focused therapy" OR "solution-focused therapeutic" OR "solution-focused therapeutics" OR "solution-focused therapeutical" OR "solution-focussed therapies" OR "solution-focussed therapy" OR "solution-focussed therapeutic" OR "solution-focussed therapeutics" OR "solution-focussed therapeutical" OR "solution focused therapies" OR "solution focused therapy" OR "solution focused therapeutic" OR "solution focused therapeutics" OR "solution focused therapeutical" OR "solution focussed therapies" OR "solution focussed therapy" OR "solution focussed therapeutic" OR "solution focussed therapeutics" OR "solution focussed therapeutical" OR "self-control therapies" OR "self-control therapy" OR "self-control therapeutics" OR "self-control therapeutical" OR "self-control therapeutic" OR "self-control training" OR "self-control trainings" OR "self control therapies" OR "self control therapy" OR "self control therapeutics" OR "self control therapeutical" OR "self control therapeutic" OR "self control training" OR "self control trainings" OR "compassion-focused" OR "compassion-focussed" OR "compassion focused" OR "compassion focussed" OR "therapies" OR "therapy" OR "therapie" OR "therapist" OR "therapists" OR "therapeut" OR "treatment" OR "treatments" OR "constructivist" OR "therapies" OR "therapy" OR "therapie" OR "therapist" OR "therapists" OR "therapeut" OR "treatment" OR "treatments" OR "exposure" OR "relaxation" OR "EMDR" OR "eye movement and reprocessing" OR "panic management" OR "response prevention" OR "ERP"
AND
"social anxiety" or "shy" or "shyness" or "test anxiety" or "gad" or "generalized anxiety" or "generalised anxiety" or "worry" or "panic" or "agoraphobi*" OR "anxiety disorder" or "anxiety disorders" or "SAD" or "social phobia" or "social anxiety disorder" or "arachnophobia" or "ophidiophobia" or "acrophobia" or "agoraphobia" or "cynophobia or "claustrophobia" or "mysophobia or "aerophobia" or "trypophobia or "carcinophobia" or "thanatophobia" or "glossophobia" or "monophobia" or "ornithophobia" or "alektorophobia" or "trypanophobia" or "anthropophobia" or "aquaphobia" or "autophobia" or "hemophobia" or "xenophobia" or "ailurophobia" or "nyctophobia" or "phobophobia" or "philophobia" or "triskaidekaphobia" or "emetophobia" or "entomophobia" or "zoophobia" or "scelerophobia" or "cibophobia" or "tokophobia" or "pseudodysphagia" or "gerascophobia" or "technophobia" or "ergophobia" or "coulrophobia" or "hotophobia" or "numerophobia" or "taphophobia"

Embase:
'psychotherapy'/exp OR psychotherap* OR 'psychotherapy' OR 'psychotherapy' OR 'psychotherapies' OR 'psychotherapeutic' OR 'psychotherapeutical' OR 'psychotherapeutics' OR 'Behavior Therapy'/exp OR 'Cognitive Behavioral Therapy'/exp OR 'CBT' OR 'behavior therapies' OR 'behavior therapy' OR 'behavior therapeutic' OR 'behavior therapeutical' OR 'behavior therapeutics' OR 'behavior therapeutist' OR 'behavior therapeutists' OR 'behavior treatment' OR 'behavior treatments' OR 'behaviors therapies' OR 'behaviors therapy' OR 'behaviors therapeutics' OR 'behaviors therapeutic' OR 'behaviors therapeutical' OR 'behaviors therapeutist' OR 'behaviors therapeutists' OR 'behaviors

treatment' OR 'behaviors treatments' OR 'behavioral therapies' OR 'behavioral therapy' OR 'behavioral therapeutics' OR 'behavioral therapeutic' OR 'behavioral therapeutical' OR 'behavioral therapeutist' OR 'behavioral therapeutists' OR 'behavioral treatment' OR 'behavioral treatments' OR 'behaviour therapies' OR 'behaviour therapy' OR 'behaviour therapeutic' OR 'behaviour therapeutical' OR 'behaviour therapeutics' OR 'behaviour therapeutist' OR 'behaviour therapeutists' OR 'behaviour treatment' OR 'behaviour treatments' OR 'behaviours therapies' OR 'behaviours therapy' OR 'behaviours therapeutics' OR 'behaviours therapeutic' OR 'behaviours therapeutical' OR 'behaviours therapeutist' OR 'behaviours therapeutists' OR 'behaviours treatment' OR 'behaviours treatments' OR 'behavioural therapies' OR 'behavioural therapy' OR 'behavioural therapeutics' OR 'behavioural therapeutic' OR 'behavioural therapeutical' OR 'behavioural therapeutist' OR 'behavioural therapeutists' OR 'behavioural treatment' OR 'behavioural treatments' OR 'cognition therapies' OR 'cognition therapie' OR 'cognition therapy' OR 'cognition therapeutical' OR 'cognition therapeutic' OR 'cognition therapeutics' OR 'cognition therapeutist' OR 'cognition therapeutists' OR 'cognition treatment' OR 'cognition treatments' OR 'cognitive therapies' OR 'cognitive therapy' OR 'cognitive therapeutic' OR 'cognitive therapeutics' OR 'cognitive therapeutical' OR 'cognitive therapeutist' OR 'cognitive therapeutists' OR 'cognitive treatment' OR 'cognitive treatments' OR 'cognitive restructuring' OR 'Emotion Focused Therapy'/exp OR 'Psychoanalysis'/exp OR 'psychoanalysis' OR 'psychoanalytic' OR 'psychoanalytical' OR 'Psychodynamic Psychotherapy'/exp OR 'psychodynamic' OR 'Psychotherapeutic Counseling'/exp OR 'counselling' OR 'counseling' OR 'problem-solving' OR 'problem solving' OR 'mindfulness' OR 'acceptance and commitment' OR 'assertiveness training' OR 'behavior activation' OR 'behaviors activation' OR 'behavioral activation' OR 'behaviour activation' OR 'behaviours activation' OR 'behavioural activation' OR 'metacognitive therapies' OR 'metacognitive therapy' OR 'metacognitive therapeutic' OR 'metacognitive therapeutics' OR 'metacognitive therapeutical' OR 'metacognitive therapeutist' OR 'metacognitive therapeutists' OR 'metacognitive treatment' OR 'metacognitive treatments' OR 'meta-cognitive therapies' OR 'meta-cognitive therapy' OR 'meta-cognitive therapeutic' OR 'meta-cognitive therapeutics' OR 'meta-cognitive therapeutical' OR 'meta-cognitive therapeutist' OR 'meta-cognitive therapeutists' OR 'meta-cognitive treatment' OR 'meta-cognitive treatments' OR 'Solution Focused Therapy'/exp OR 'solution-focused therapies' OR 'solution-focused therapy' OR 'solution-focused therapeutic' OR 'solution-focused therapeutics' OR 'solution-focused therapeutical' OR 'solution-focussed therapies' OR 'solution-focussed therapy' OR 'solution-focussed therapeutic' OR 'solution-focussed therapeutics' OR 'solution-focussed therapeutical' OR 'solution focused therapies' OR 'solution focused therapy' OR 'solution focused therapeutic' OR 'solution focused therapeutics' OR 'solution focused therapeutical' OR 'solution focussed therapies' OR 'solution focussed therapy' OR 'solution focussed therapeutic' OR 'solution focussed therapeutics' OR 'solution focussed therapeutical' OR 'self-control therapies' OR 'self-control therapy' OR 'self-control therapeutics' OR 'self-control therapeutical' OR 'self-control therapeutic' OR 'self-control training' OR 'self-control trainings' OR 'self control therapies' OR 'self control therapy' OR 'self control therapeutics' OR 'self control therapeutical' OR 'self control therapeutic' OR 'self control training' OR 'self control trainings' OR 'compassion-focused' OR 'compassion-focussed' OR 'compassion focused' OR 'compassion focussed' OR 'exposure' OR 'relaxation' OR 'EMDR' OR 'eye movement and reprocessing' OR 'panic management' OR 'response prevention' OR 'ERP'
AND
'anxiety disorder'/exp OR 'anxiety disorder' OR 'gad'/exp OR 'gad' OR 'generalized anxiety disorder'/exp OR 'generalized anxiety disorder' OR 'generalised anxiety disorder'/exp OR 'generalised anxiety disorder' OR 'generalized anxiety' OR 'generalised anxiety' OR 'worry' OR 'social phobia'/exp OR 'social phobia' OR 'social anxiety disorder'/exp OR 'social anxiety disorder' OR 'social anxiety' OR 'acute stress disorder'/exp OR 'acute stress disorder' OR 'acute stress' OR 'panic'/exp OR 'panic' OR 'agorophobia' OR 'Arachnophobia' OR 'Ophidiophobia' OR 'Acrophobia' OR 'Agoraphobia' OR 'Cynophobia' OR 'Claustrophobia' OR 'Mysophobia' OR 'Aerophobia' OR 'Trypophobia' OR 'Carcinophobia' OR 'Thanatophobia' OR 'Glossophobia' OR 'Monophobia' OR 'Ornithophobia' OR 'Alektorophobia' OR 'Trypanophobia' OR 'Anthropophobia' OR 'Aquaphobia' OR 'Autophobia' OR 'Hemophobia' OR 'Xenophobia' OR 'Ailurophobia' OR 'Nyctophobia' OR 'Phobophobia' OR 'Philophobia' OR 'Triskaidekaphobia' OR 'Emetophobia' OR 'Entomophobia' OR 'Zoophobia' OR 'Scelerophobia' OR 'Cibophobia' OR 'Tokophobia' OR 'Pseudodysphagia' OR 'Gerascophobia' OR 'Technophobia' OR 'Ergophobia' OR 'Coulrophobia' OR 'Photophobia' OR 'Numerophobia' OR 'Taphophobia'

Cochrane Library:

ID	Search
#1	"social anxiety disorder":ti,ab,kw (Word variations have been searched)
#2	"GAD":ti,ab,kw (Word variations have been searched)
#3	"social anxiety":ti,ab,kw (Word variations have been searched)
#4	"test anxiety":ti,ab,kw (Word variations have been searched)
#5	"generalised anxiety disorder":ti,ab,kw (Word variations have been searched)
#6	"generalised anxiety":ti,ab,kw (Word variations have been searched)
#7	"worry":ti,ab,kw (Word variations have been searched)
#8	"panic":ti,ab,kw (Word variations have been searched)
#9	"panic disorder":ti,ab,kw (Word variations have been searched)
#10	"agoraphobia":ti,ab,kw (Word variations have been searched)
#11	"phobia":ti,ab,kw (Word variations have been searched)
#12	#1 OR #2 OR #3 OR #4 OR #5 OR #6 OR #7 OR #8 OR #9 OR #10 OR #11
#13	"psychotherapy":kw (Word variations have been searched)
#14	"CBT":ti,ab,kw (Word variations have been searched)
#15	cognitive behavi* therap*:ti,ab,kw (Word variations have been searched)
#16	psychodynamic:ti,ab,kw (Word variations have been searched)
#17	"psychoanalysis":ti,ab,kw (Word variations have been searched)
#18	psychoanalys*:ti,ab,kw (Word variations have been searched)
#19	"counseling":ti,ab,kw (Word variations have been searched)
#20	"problem solving":ti,ab,kw (Word variations have been searched)
#21	"acceptance and commitment":ti,ab,kw (Word variations have been searched)
#22	"assertiveness training":ti,ab,kw (Word variations have been searched)
#23	"behavioral activation":ti,ab,kw (Word variations have been searched)
#24	"behavioural activation":ti,ab,kw (Word variations have been searched)
#25	"mindfulness":ti,ab,kw (Word variations have been searched)
#26	"metacognitive therap*":ti,ab,kw (Word variations have been searched)
#27	"solution focused therap*":ti,ab,kw (Word variations have been searched)
#28	"self-control therap*":ti,ab,kw (Word variations have been searched)
#29	"self-control training":ti,ab,kw (Word variations have been searched)
#30	"exposure":ti,ab,kw (Word variations have been searched)

#31 "relaxation":ti,ab,kw (Word variations have been searched)
#32 "EMDR":ti,ab,kw (Word variations have been searched)
#33 "eye movement desensitization":ti,ab,kw (Word variations have been searched)
#34 "panic management":ti,ab,kw (Word variations have been searched)
#35 "response prevention":ti,ab,kw (Word variations have been searched)
#36 #13 OR #14 OR #15 OR #16 OR #17 OR #18 OR #19 OR #20 OR #21 OR #22 OR #23 #24 OR #25 OR #26 OR #27 OR #28 OR #29 OR #30 OR #31 OR #32 OR #33 OR #34 OR #35
#37 #12 AND #36 in Trials

Posttraumatic Stress Disorder

Database: Ovid MEDLINE®, Ovid MEDLINE® In-Process & Other Non-Indexed Citations

Pharmacologic interventions

1. stress disorders, post-traumatic/
2. ("posttraumatic stress disorder" or "post traumatic stress disorder" or PTSD).ti,ab.
3. 1 or 2
4. exp Drug Therapy/ or dt.fs. or (medication* or pharmacologic* or pharmaco-therap* or pharmacotherap*).ti,ab.
5. (drug* adj2 (therap* or treatment*)).ti,ab. or exp Adrenergic alpha-Antagonists/ or Sympatholytics/ or Doxazosin/ or Prazosin/
6. ("adrenergic alpha antagonist*" or "adrenergic receptor block*" or "alpha adrenergic antagonist*" or "alpha block*" or antiadrenergic* or doxazosin or prazosin or sympatholytic* or terazosin).ti,ab. or exp Antipsychotic Agents/
7. exp Benzodiazepines/ or ("anti-psychotic*" or antipsychotic* or FGA* or SGA* or aripiprazole or asenapine or brexpiprazole or cariprazine or chlorpromazine or clozapine or fluphenazine or haloperidol or iloperidone or loxapine or lurasidone or olanzapine or paliperidone or perphenazine or pimozide or quetiapine or risperidone or thioridazine or thiothixene or trifluoperazine or ziprasidone).ti,ab.
8. (alprazolam or benzodiazepine* or benzodiazepinone* or chlordiazepoxide or clonazepam or clorazepate or diazepam or estazolam or flurazepam or lorazepam or midazolam or oxazepam or quazepam or temazepam or triazolam).ti,ab. or exp Monoamine Oxidase Inhibitors/
9. (("monoamine oxidase" adj2 inhibitor*) or MAOI or isocarboxazid or phenelzine or selegiline or tranylcypromine).ti,ab.
10. carbamazepine/ or clonidine/ or lithium/ or pregabalin/ or valproic acid/ or exp Anticonvulsants/ or exp Antimanic Agents/
11. exp Cyclohexanecarboxylic Acids/ or (anticonvuls* or carbamazepine or clonidine or divalproex or gabapentin or lamotrigine or lithium or oxcarbazepine or pregabalin or tiagabine or topiramate or valproate or "valproic acid").ti,ab.
12. exp "hypnotics and sedatives"/ or exp anti-anxiety agents/ or ("anti anxiety" or antianxiety or buspirone or diphenhydramine or eszopiclone or guanfacine or hydroxizine or hypnotic* or ramelteon or sedative* or suvorexant or tasimelteon or zaleplon or zolpidem or zopiclone).ti,ab.
13. (antidepressant* or "anti-depressant*" or "selective serotonin" or (serotonin adj3 reuptake) or SNRI* or SSRI* or tricyclic or amitriptyline or amoxapine or bupropion or citalopram or clomipramine or desipramine or desvenlafaxine or doxepin or duloxetine or escitalopram or fluoxetine or fluvoxamine or hydroxizine or imipramine or levomilnacipran or maprotiline or milnacipran or mirtazapine or nefazodone or nortriptyline or paroxetine or protriptyline or sertraline or trazadone or trimipramine or venlafaxine or vilazodone or vortioxetine).ti,ab. or exp Antidepressive Agents/
14. exp Amphetamines/ or (amphetamine or armodafanil or atomoxetine or dexmethylphenidate or dextroamphetamine or lisdexamphetamine or MDMA or methamphetamine or methylphenidate or modafanil).ti,ab.
15. exp Steroids/ or (DHEA or hydrocortisone or steroid*).ti,ab. or exp Cannabinoids/
16. Cannabis/ or Medical Marijuana/ or (cannabi* or marijuana or tetrahydrocannabinol or THC).ti,ab.
17. ketamine/ or ketamine.ti,ab. or Propranolol/ or propranolol.ti,ab.
18. 4 or 5 or 6 or 7 or 8 or 9 or 10 or 11 or 12 or 13 or 14 or 15 or 16 or 17
19. 3 and 18
20. exp Randomized Controlled Trials as Topic/ or exp Randomized Controlled Trial/
21. double-blind method/ or random allocation/ or single-blind method/ or Placebos/
22. (random* or control* or trial or sham or placebo* or blind* or dumm* or mask*).ti,ab,kw.
23. 20 or 21 or 22
24. 19 and 23
25. ("20220929" or 2022093* or 20221* or 2023*).ed,ez.
26. 24 and 25

Nonpharmacologic interventions

1. stress disorders, post-traumatic/ or ("posttraumatic stress disorder" or "post traumatic stress disorder" or PTSD).ti,ab.
2. exp Psychotherapy/ or exp Complementary Therapies/ or exp Convulsive Therapy/
3. Hyperbaric Oxygenation/ or Transcranial Magnetic Stimulation/ or exp Rehabilitation/
4. exp Dietary Supplements/
5. exp "Delivery of Health Care, Integrated"/ or exp Self-Help Groups/ or exp peer group/
6. exp social support/ or exp Telemedicine/ or telephone/ or exp cell phone/
7. (therap* or psychotherap* or counsel* or nonpharma* or non-pharma* or ("alternative medicine" or acupuncture or "animal assist*" or art or "cell phone" or "cognitive behavior*" or CBT or complementary or dance or drama or electroconvulsive or ECT or exercise or "eye movement desensitization and reprocessing" or EMDR or family or "hyperbaric oxygen*" or integrated or meditation or "mind body" or mindfulness or music or "prolonged exposure" or relaxation or "seeking safety" or "self help" or "tai chi" or "tai ji" or "text messag*" or "transcranial magnetic stimulation" or TMS or yoga)).ti,ab.
8. 2 or 3 or 4 or 5 or 6 or 7
9. 1 and 8
10. exp Randomized Controlled Trials as Topic/ or exp Randomized Controlled Trial/ or double-blind method/ or random allocation/ or single-blind method/ or Placebos/ or (random* or control* or trial or sham or placebo* or blind* or dumm* or mask*).ti,ab,kw.
11. 9 and 10
12. ("20220929" or 2022093* or 20221* or 2023*).ed,ez.
13. 11 and 12

Database: PTSDpubs (formerly PILOTS)

(MAINSUBJECT.EXACT("PTSD") OR MAINSUBJECT.EXACT("PTSD (DSM-III-R)") OR MAINSUBJECT.EXACT("PTSD (DSM-III)") OR MAINSUBJECT.EXACT("PTSD (DSM-IV)") OR MAINSUBJECT.EXACT("PTSD (DSM-5)") OR MAINSUBJECT.EXACT("Complex PTSD") OR MAINSUBJECT.EXACT("PTSD (ICD-11)") OR MAINSUBJECT.EXACT("PTSD (ICD-10)") OR

MAINSUBJECT.EXACT("PTSD (ICD-9)") OR (ptsd OR "posttraumatic stress disorder" OR "post-traumatic stress disorder")) AND (MAINSUBJECT.EXACT("Randomized Clinical Trial") OR ti(random* OR control* OR trial))

Database: APA *PsycInfo*®

1. exp posttraumatic stress disorder/ or ("post traumatic stress disorder" or "posttraumatic stress disorder" or PTSD).ti,ab.
2. exp treatment/ or exp stimulation/ or exp electroconvulsive shock/ or exp TELEMEDICINE/ or exp counseling/ or exp support groups/ or (therap* or psychotherap* or counsel* or nonpharma* or non-pharma*).ti,ab. or ("alternative medicine" or acupuncture or "animal assist*" or art or "cell phone" or "cognitive behavior*" or CBT or complementary or dance or drama or electroconvulsive or ECT or exercise or "eye movement desensitization and reprocessing" or EMDR or family or "hyperbaric oxygen*" or integrated or meditation or "mind body" or mindfulness or music or "prolonged exposure" or relaxation or "seeking safety" or "self help" or "tai chi" or "tai ji" or "text messag*" or "transcranial magnetic stimulation" or TMS or yoga).ti,ab.
3. treatment effectiveness evaluation/ or Treatment Outcomes/ or followup studies/ or (random* or control* or trial or sham or placebo* or blind* or dumm* or mask*).ti,ab.
4. 1 and 2 and 3
5. ("20220929" or 2022093* or 20221* or 2023*).up.
6. 4 and 5

Database: EBM Reviews - Cochrane Central Register of Controlled Trials

1. Stress Disorders, Post-Traumatic/ or ("posttraumatic stress disorder" or "post traumatic stress disorder" or "ptsd").ti,ab.
2. (dt or pc or rh or th).fs. or exp treatment outcome/ or exp therapeutics/ or (treatment or therap* or intervention*).ti,ab,kw.
3. 1 and 2
4. limit 3 to medline records
5. 3 not 4
6. ("20220929" or 2022093* or 20221* or 2023*).up.
7. 5 and 6

Database: Elsevier® Embase

('posttraumatic stress disorder'/exp/mj OR 'posttraumatic stress disorder'/exp OR 'posttraumatic stress disorder' OR 'posttraumatic stress disorder':ab,ti OR 'post traumatic stress disorder':ab,ti OR 'ptsd':ab,ti) AND [randomized controlled trial]/lim AND ('randomized controlled trial'/exp OR 'randomized controlled trial') AND [embase]/lim NOT ([embase]/lim AND [medline]/lim) AND [28-09-2022]/sd NOT [03-03-2023]/sd

Database: EBSCO® CINAHL

S2 ((MM "Stress Disorders, Post-Traumatic+") OR (AB "post traumatic stress disorder" OR AB "posttraumatic stress disorder" OR AB "ptsd") OR (TI "post traumatic stress disorder" OR TI "posttraumatic stress disorder" OR TI "ptsd")) AND ((TI random* or AB random* or PT clinical trial or PT randomized controlled trial))
S1 ((MM "Stress Disorders, Post-Traumatic+") OR (AB "post traumatic stress disorder" OR AB "posttraumatic stress disorder" OR AB "ptsd") OR (TI "post traumatic stress disorder" OR TI "posttraumatic stress disorder" OR TI "ptsd")) AND ((TI random* or AB random* or PT clinical trial or PT randomized controlled trial))

Database: Elsevier® Scopus

((TITLE ((random* OR control* OR trial* OR sham* OR placebo* OR blind*))) AND (TITLE-ABS-KEY (("post traumatic stress disorder" OR "posttraumatic stress disorder" OR "ptsd")))) AND LOAD-DATE > 20220928 AND (LIMIT-TO (PUBYEAR , 2023) OR LIMIT-TO (PUBYEAR , 2022))

Obsessive-Compulsive Disorder

International databases:
PubMed
("obsessive compulsive"[Title/Abstract] OR "OCD"[Title/Abstract]) AND (randomizedcontrolledtrial[Filter])

Embase
'obsessive compulsive':ti,ab,kw OR ocd:ti,ab,kw
AND 'randomized controlled trial'/de

PsycINFO
MA "obsessive compulsive" or OCD
Expanders - Apply equivalent subjects
Narrow by Methodology: - clinical trial
Search modes - Boolean/Phrase

WHO's Registry (international clinical trials registry platform of WHO)
"obsessive compulsive" or OCD

Chinese databases
CNKI (TKA=Title/Keyword/Abstract; FT=Full Text)
TKA = ('强迫症' + '强迫性神经官能症' + '强迫性障碍' + '强迫障碍' + '强迫性神经症') AND TKA = ('治疗' + '疗法' + '干预' + '医治' + '辅导') AND (FT= ('临床试验'+'随机对照试验') OR TKA= ('临床'+'试验'+'随机'+'交叉'+'组'+'安慰剂'+'双盲'+'单盲'+'三盲'+'平行组'+'对照研究'+'对照试验'+'对照设计'+'随机对照'+'随机对照研究'+'随机对照临床试验'+'随机对照临床研究'+'随机对照实验'+'随机化'+'RCT'))
同义词扩展

WanFang (题名或关键词=Title or Keyword; 摘要=Abstract)
((题名或关键词:(强迫症 or 强迫性神经官能症 or 强迫性障碍 or 强迫障碍 or 强迫性神经症) AND 题名或关键词:(治疗 or 疗法 or 干预 or 医治 or 辅导)) or (摘要:(强迫症 or 强迫性神经官能症 or 强迫性障碍 or 强迫障碍 or 强迫性神经症) AND 摘要:(治疗 or 疗法 or 干预 or 医治 or 辅导)))
AND (全部: (临床试验 OR 随机对照试验) OR 题名或关键词: (临床 OR 试验 OR 随机 OR交叉 OR 组 OR 安慰剂OR 双盲OR 单盲OR 三盲OR 平行组 OR 对照研究 OR 对照试验 OR 对照设计 OR 随机对照 OR 随机对照研究 OR 随机对照临床试验 OR 随机对照临床研究 OR 随机对照实验 OR 随机化 OR RCT) OR 摘要: (临床 OR 试验 OR 随机 OR交叉 OR 组 OR 安慰剂OR 双盲OR 单盲OR 三盲OR 平行组 OR 对照研究 OR 对照试验 OR 对照设计 OR 随机对照 OR 随机对照研究 OR 随机对照临床试验 OR 随机对照临床研究 OR 随机对照实验 OR 随机化 OR RCT))
主题词扩展

WeiPu (M=Title/Keyword; R=Abstract)
((M=(强迫症+强迫性神经官能症+强迫性障碍+强迫障碍+强迫性神经症) AND M=(治疗+疗法+干预+医治+辅导)) OR (R=(强迫症+强迫性神经官能症+强迫性障碍+强迫障碍+强迫性神经症) AND R=(治疗+疗法+干预+医治+辅导)))
AND (U=(临床试验 OR 随机对照试验) OR (M=(临床 OR 试验 OR 随机 OR交叉 OR 组 OR 安慰剂OR 双盲OR 单盲OR 三盲OR 平行组 OR 对照研究 OR 对照试验 OR 对照设计 OR 随机对照 OR 随机对照研究 OR 随机对照临床试验 OR 随机对照临床研究 OR 随机对照实验 OR 随机化 OR RCT)) OR (R=(临床 OR 试验 OR 随机 OR交叉 OR 组 OR 安慰剂OR 双盲OR 单盲OR 三盲OR 平行组 OR 对照研究 OR 对照试验 OR 对照设计 OR 随机对照 OR 随机对照研究 OR 随机对照临床试验 OR 随机对照临床研究 OR 随机对照实验 OR 随机化 OR RCT)))

ChiCTR(Chinese Clinical Trial Registry)
强迫

Insomnia

PubMed:

Main Search:
("psychotherapy"[Mesh] OR "psychotherap*"[All Fields] OR "cognitive behavioural therapy"[All Fields] OR "cognitive behavioral therapy"[All Fields] OR "CBT"[All Fields] OR "CBTI"[All Fields] OR "CBT-I"[All Fields] OR "cognitive therapy"[All Fields] OR "behavioural therapy"[All Fields] OR "behavioral therapy"[All Fields] OR "cognitive restructuring"[All Fields] OR "third wave"[All Fields] OR "mindfulness"[All Fields] OR "acceptance and commitment"[All Fields] OR "sleep restriction"[All Fields] OR "stimulus control"[All Fields])
AND
("hypnotic"[All Fields] OR "sleep medication"[All Fields] OR "benzodiazepines"[Mesh] OR "benzodiazepine"[All Fields] OR "brotizolam"[All Fields] OR "diazepam"[All Fields] OR "estazolam"[All Fields] OR "flunitrazepam"[All Fields] OR "flurazepam"[All Fields] OR "haloxazolam"[All Fields] OR "loprazolam"[All Fields] OR "lorazepam"[All Fields] OR "lormetazepam"[All Fields] OR "nimetazepam"[All Fields] OR "nitrazepam"[All Fields] OR "quazepam"[All Fields] OR "rilmazafone"[All Fields] OR "temazepam"[All Fields] OR "triazolam"[All Fields] OR "trazodone"[All Fields] OR "eszopiclone"[All Fields] OR "zopiclone"[All Fields] OR "doxylamine"[All Fields] OR "zolpidem"[All Fields] OR "seltorexant"[All Fields] OR "lemborexant"[All Fields] OR "suvorexant"[All Fields] OR "zaleplon"[All Fields])
AND
("sleep initiation and maintenance disorders"[Mesh] OR "insomnia"[All Fields])
AND
("randomized controlled trial"[pt] OR "controlled clinical trial"[pt] OR randomized[tiab] OR placebo[tiab] OR "clinical trials as topic"[Mesh:NoExp] OR randomly[tiab] OR trial[ti] NOT (animals[Mesh] NOT humans[Mesh]))

iCBT-specific Update Search:
(Psychotherapy [MH] OR psychotherap*[All Fields] OR "cognitive behavioural therapy"[All Fields] OR "cognitive behavioral therapy"[All Fields] OR "CBT"[All Fields] OR "CBTI"[All Fields] OR "CBT-I"[All Fields] OR "cognitive therapy"[All Fields] OR "behavioural therapy"[All Fields] OR "behavioral therapy"[All Fields] OR "cognitive restructuring"[All Fields] OR "mindfulness"[All Fields] OR "sleep restriction"[All Fields] OR "stimulus control"[All Fields]) AND (digital or internet or smartphone or web or sleepio or shuti)
AND
("sleep initiation and maintenance disorders"[Mesh] [MeSH Terms] OR insomnia[All Fields])
AND
(randomized controlled trial[pt] OR controlled clinical trial[pt] OR randomized[tiab] OR placebo[tiab] OR clinical trials as topic[mesh:noexp] OR randomly[tiab] OR trial[ti] NOT (animals[mh] NOT humans [mh]))

Cochrane Central Register of Controlled Trials:
([mh psychotherapy] OR psychotherap* OR "cognitive behavioural therapy" OR "cognitive behavioral therapy" OR CBT OR CBTI OR CBT-I OR "cognitive therapy" OR "behavioural therapy" OR "behavioral therapy" OR "cognitive restructuring" OR "third wave" OR mindfulness OR "acceptance and commitment" OR "sleep restriction" OR "stimulus control")
AND
(hypnotic OR "sleep medication" OR [mh benzodiazepines] OR benzodiazepine OR brotizolam OR diazepam OR estazolam OR flunitrazepam OR flurazepam OR haloxazolam OR loprazolam OR lorazepam OR lormetazepam OR nimetazepam OR nitrazepam OR quazepam OR rilmazafone OR temazepam OR triazolam OR trazodone OR eszopiclone OR zopiclone OR doxylamine OR zolpidem OR seltorexant OR lemborexant OR suvorexant OR zaleplon)
AND
([mh "sleep initiation and maintenance disorders"] OR insomnia)

PsycINFO:
((MH psychotherapy+) OR psychotherap* OR "cognitive behavioural therapy" OR "cognitive behavioral therapy" OR CBT OR CBTI OR CBT-I OR "cognitive therapy" OR "behavioural therapy" OR "behavioral therapy" OR "cognitive restructuring" OR "third wave" OR mindfulness OR "acceptance and commitment" OR "sleep restriction" OR "stimulus control")
AND
(hypnotic OR "sleep medication" OR (MH benzodiazepines+) OR benzodiazepine OR brotizolam OR diazepam OR estazolam OR flunitrazepam OR flurazepam OR haloxazolam OR loprazolam OR lorazepam OR lormetazepam OR nimetazepam OR nitrazepam OR quazepam OR rilmazafone OR temazepam OR triazolam OR trazodone OR eszopiclone OR zopiclone OR doxylamine OR zolpidem OR seltorexant OR lemborexant OR suvorexant OR zaleplon)
AND
((MH "sleep initiation and maintenance disorders+") OR insomnia)
AND
(MH randomized controlled trials OR MH double-blind studies OR MH single-blind studies OR MH random assignment OR MH pretest-posttest design OR MH cluster sample OR TI (randomised OR randomized) OR AB (random*) OR TI (trial) OR (MH (sample size) AND AB (assigned OR allocated OR control)) OR MH (placebos) OR PT (randomized controlled trial) OR AB (control W5 group) OR MH (crossover design) OR MH (comparative studies) OR AB (cluster W3 RCT)) NOT ((MH animals+ OR MH animal studies OR TI (animal model*)) NOT MH human)

WHO International Clinical Trials Registry Platform:
(psychotherap* OR "cognitive behavioural therapy" OR "cognitive behavioral therapy" OR "CBT" OR "CBTI" OR "CBT-I" OR "cognitive therapy" OR "behavioural therapy" OR "behavioral therapy" OR "cognitive restructuring" OR "third wave" OR mindfulness OR "acceptance and commitment" OR "sleep restriction" OR "stimulus control")
AND
(hypnotic OR "sleep medication" OR benzodiazepines OR benzodiazepine OR brotizolam OR diazepam OR estazolam OR flunitrazepam OR flurazepam OR haloxazolam OR loprazolam OR lorazepam OR lormetazepam OR nimetazepam OR nitrazepam OR quazepam OR rilmazafone OR temazepam OR triazolam OR trazodone OR eszopiclone OR zopiclone OR doxylamine OR zolpidem OR seltorexant OR lemborexant OR suvorexant OR zaleplon)
AND
("sleep initiation and maintenance disorders " OR insomnia)

S2. *Definition & identification of prescription digital therapeutics.*

For the current paper, prescription digital therapeutics (prescription DTx) were defined as "therapeutic interventions through a clinically evaluated, patient-directed software application intended to improve the process of treating mental disorders". This definition was derived from Fürstenau, Gersch & Schreiter (2023). As "prescription" DTx, these interventions had to be (1) available via prescription by a health professional (e.g., a GP), for patients (2) diagnosed with a ICD/DSM mental disorder, for which the DTx has received clearance by (3) a national regulatory agency via (4) a designated regulatory pathway.

Based on published reviews and overview articles, as well as current information provided by the "Digital Therapeutics Alliance" (DTA, 2024), we identified the following countries in which prescription DTx can receive designated regulatory approval:

- **United States:** For an overview on regulatory pathways for DTx provided by the U.S. Food and Drug Administration (FDA), see Watson et al. (2023). FDA-cleared prescription DTx were identified from published reviews (Phan et al., 2023; Wang, Lee & Shin, 2023). FDA clearance of "SleepioRx" was identified via a 510(k) Premarket Notification (FDA, 2024).

- **Germany**: An overview of regulatory pathways for DTx in Germany ("Digitale Gesundheitsanwendungen"/"DiGAs"), see Ludewig et al. (2021). The German national regulatory agency (BfArM) maintains a national registry for all approved prescription DTx (diga.bfarm.de). This registry was screened to identify DTx and the evidence submitted to obtain approval (deadline: November 27th, 2024).

- **Belgium**: Belgium has developed a regulatory pathway for DTx modeled after the German system. Medicinal products are listed in an online repository (mhealthbelgium.be/apps). Belgium maintains a tiered system, whereby applications on Level 3 are financed by the National Institute for Health and Disability Insurance (NIHDI). As of November 27th, 2024, we did not find any suitable applications listed as Level 3-DTx.

- **France**: In 2024, France has installed a regulatory pathway for DTx modeled after the German system, *Prise en charge anticipée numérique* (PECAN; Gnius, 2024). As of November 27th, 2024, we are not aware of any approved mental health DTx under this system.

- **Japan**: A review by Nomura et al. (2024) lists available prescription DTx in Japan as of September 2024. This publication was used to identify Japanese DTx trials in the current study.

A global overview of regulatory pathways for DTx is summarized by Fürstenau, Gersch & Schreiter (2023). In the United Kingdom, the National Health Service (NHS) is also funding the development of DTx, which are offered using universal reimbursement. This system was not screened for available DTx because it diverges from the primarily market-oriented approach for prescription DTx followed in other countries (ibid.).

References

DTA (2023). *DTx by country.* https://dtxalliance.org/understanding-dtx/dtx-by-country/. Archived at https://archive.fo/DdOIH.

FDA (2024). *Computerized Behavioral Therapy Device For Insomnia.* https://www.accessdata.fda.gov/scripts/cdrh/cfdocs/cfpmn/pmn.cfm?ID=K233577

Fürstenau, D., Gersch, M., & Schreiter, S. (2023). Digital therapeutics (DTx). *Business & Information Systems Engineering*, *65*(3), 349-360.

Gnius (2024). *Prise en charge anticipée numérique (PECAN).* https://gnius.esante.gouv.fr/fr/financements/fiches-remboursement/prise-en-charge-anticipee-numerique-pecan

Ludewig, G., Klose, C., Hunze, L., & Matenaar, S. (2021). Digital health applications: statutory introduction of patient-centred digital innovations into healthcare. *Bundesgesundheitsblatt-Gesundheitsforschung-Gesundheitsschutz*, 1-9.

Nomura, A. (2024). Digital therapeutics in Japan: Present and future directions. *Journal of Cardiology.*

Phan, P., Mitragotri, S., & Zhao, Z. (2023). Digital therapeutics in the clinic. *Bioengineering & Translational Medicine*, *8*(4), e10536. https://doi.org/10.1002/btm2.10536

Wang, C., Lee, C., & Shin, H. (2023). Digital therapeutics from bench to bedside. *Npj Digital Medicine*, *6*(1), 1. https://doi.org/10.1038/s41746-023-00777-z

Watson, A., Chapman, R., Shafai, G., & Maricich, Y. A. (2023). FDA regulations and prescription digital therapeutics: Evolving with the technologies they regulate. *Frontiers in Digital Health*, *5*, 1086219.

S3. *PRISMA flowcharts.*

Depression

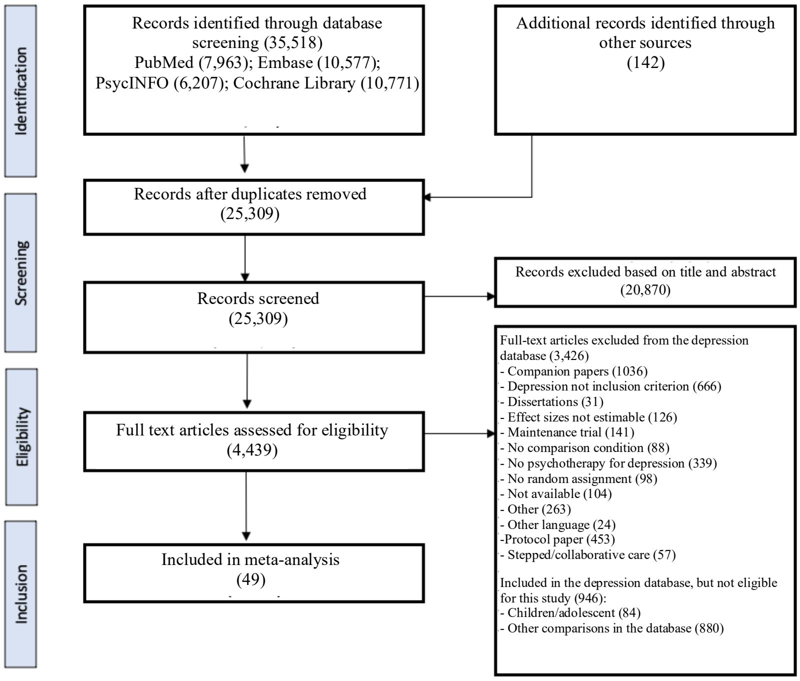

Anxiety Disorders

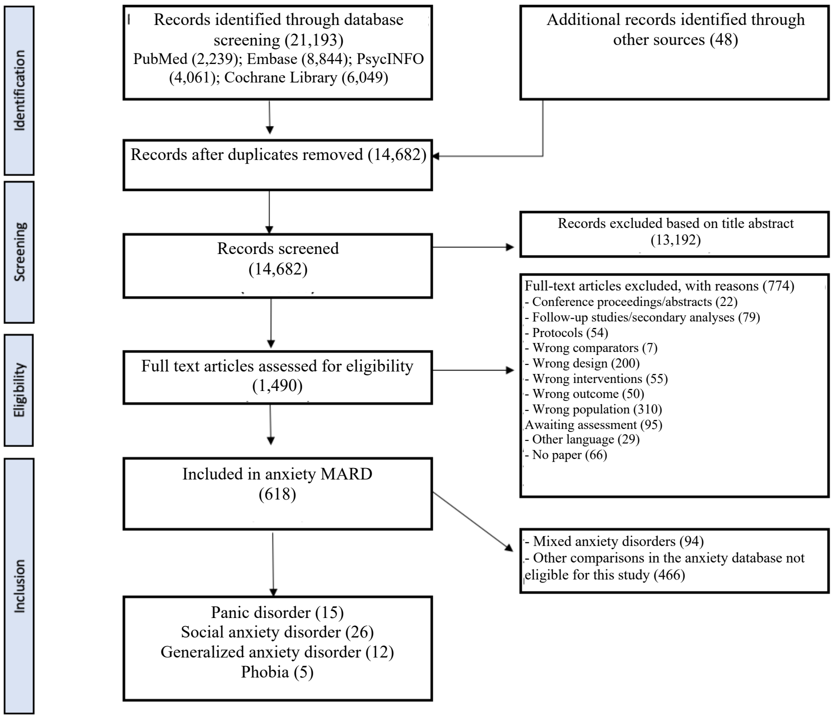

Posttraumatic Stress Disorder

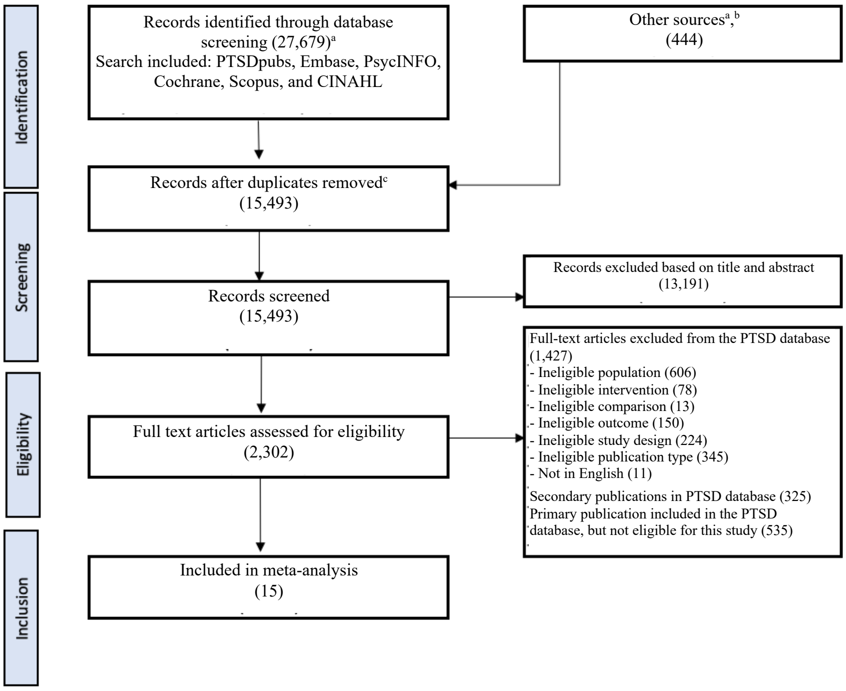

[a]Multiple update searches were performed with overlapping search dates; reported is number of unique records from each source across searches; [b]Other sources include prior reports, reference lists of relevant articles, systematic reviews, etc; [c]Number of unique records across sources.

Obsessive-Compulsive Disorder

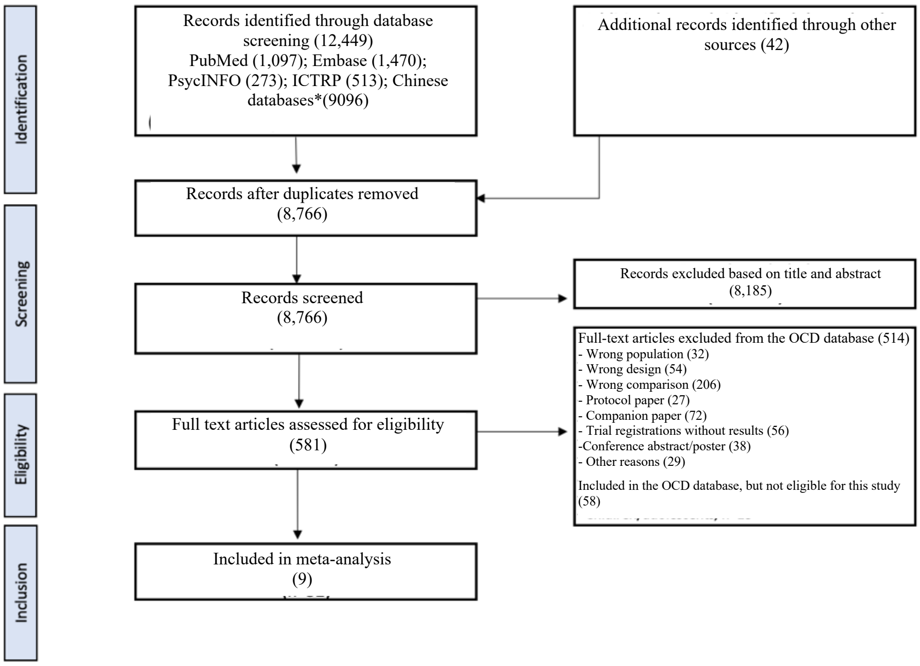

Insomnia

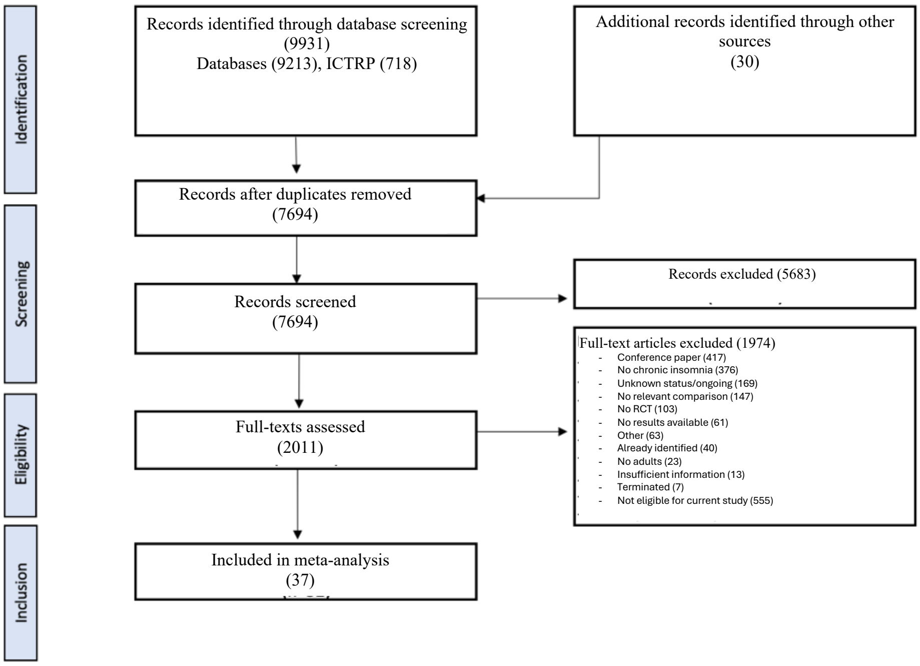
Identification
Screening
Eligibility
Inclusion
Records identified through database screening (9931) Databases (9213), ICTRP (718)
Additional records identified through other sources (30)
Records after duplicates removed (7694)
Records screened (7694)
Records excluded (5683)
Full-text articles excluded (1974)
- Conference paper (417)
- No chronic insomnia (376)
- Unknown status/ongoing (169)
- No relevant comparison (147)
- No RCT (103)
- No results available (61)
- Other (63)
- Already identified (40)
- No adults (23)
- Insufficient information (13)
- Terminated (7)
- Not eligible for current study (555)
Full-texts assessed (2011)
Included in meta-analysis (37)

S4. *References of the included studies.*

Depression

Arjadi R, Nauta MH, Scholte WF, et al. Internet-based behavioural activation with lay counsellor support versus online minimal psychoeducation without support for treatment of depression: a randomised controlled trial in Indonesia. The lancet psychiatry 2018; 5(9): 707-16.

Baumeister, H., Paganini, S., Sander, L. B., Lin, J., Schlicker, S., Terhorst, Y., Moshagen, M., Bengel, J., Lehr, D., & Ebert, D. D. (2021). Effectiveness of a Guided Internet- and Mobile-Based Intervention for Patients with Chronic Back Pain and Depression (WARD-BP): A Multicenter, Pragmatic Randomized Controlled Trial. Psychother Psychosom, 90(4), 255-268. https://doi.org/10.1159/000511881

Berger T, Hämmerli K, Gubser N, Andersson G, Caspar F. Internet-based treatment of depression: A randomized controlled trial comparing guided with unguided self-help. Cognitive Behaviour Therapy. 2011;40(4):251-66.

Carlbring P, Hagglund M, Luthstrom A, Dahlin M, Kadowaki A, Vernmark K, et al. Internet-based behavioral activation and acceptance-based treatment for depression: A randomized controlled trial. Journal of Affective Disorders. 2013;148(2-3):331-7.

Choi I, Zou J, Titov N, Dear BF, Li S, Johnston L, et al. Culturally attuned Internet treatment for depression amongst Chinese Australians: A randomised controlled trial. Journal of Affective Disorders. 2012;136(3):459-68.

Forsell E, Bendix M, Holländare F, et al. Internet delivered cognitive behavior therapy for antenatal depression: A randomised controlled trial. Journal of Affective Disorders 2017; 221: 56-64.

Garcia, A., Yáñez, A. M., Bennasar-Veny, M., Navarro, C., Salva, J., Ibarra, O., . . . Garcia-Toro, M. (2023). Efficacy of an adjuvant non-face-to-face multimodal lifestyle modification program for patients with treatment-resistant major depression: A randomized controlled trial. Psychiatry Res, 319, 114975. doi:10.1016/j.psychres.2022.114975

Johansson R, Ekbladh S, Hebert A, Lindström M, Möller S, Petitt E, et al. Psychodynamic guided self-help for adult depression through the internet: A randomised controlled trial. PloS One. 2012;7(5):e38021.

Johansson R, Sjöberg E, Sjögren M, Johnsson E, Carlbring P, Andersson T, et al. Tailored vs. standardized internet-based cognitive behavior therapy for depression and comorbid symptoms: A randomized controlled trial. PloS One. 2012;7(5):e36905.

Johansson, O., Bjärehed, J., Andersson, G., Carlbring, P., & Lundh, L. G. (2019). Effectiveness of guided internet-delivered cognitive behavior therapy for depression in routine psychiatry: A randomized controlled trial. Internet Interventions, 17. doi:10.1016/j.invent.2019.100247

Kenter, R. M. F., Cuijpers, P., Beekman, A., & van Straten, A. (2016). Effectiveness of a Web-based guided self-help intervention for outpatients with a depressive disorder: Short-term results from a randomized controlled trial. Journal of medical Internet research, 18(3).

Kessler D, Lewis G, Kaur S, Wiles N, King M, Weich S, et al. Therapist-delivered Internet psychotherapy for depression in primary care: A randomised controlled trial. Lancet. 2009;374(9690):628-34.

Kivi M, Eriksson MCM, Hange D, Petersson E-L, Vernmark K, Johansson B, et al. Internet-based therapy for mild to moderate depression in Swedish primary care: Short term results from the PRIM-NET randomized controlled trial. Cognitive Behaviour Therapy. 2014;43(4):289-98.

Lappalainen P, Langrial S, Oinas-Kukkonen H, Tolvanen A, Lappalainen R. Web-based acceptance and commitment therapy for depressive symptoms with minimal support: A randomized controlled trial. Behavior modification. 2015;39(6):805-34.

Milgrom J, Danaher BG, Gemmill AW, Holt C, Holt CJ, Seeley JR, et al. Internet cognitive behavioral therapy for women with postnatal depression: A randomized controlled trial of MumMoodBooster. Journal of Medical Internet Research. 2016;18(3):e54.

Milgrom, J., Danaher, B. G., Seeley, J. R., Holt, C. J., Holt, C., Ericksen, J., Tyler, M. S., Gau, J. M., & Gemmill, A. W. (2021). Internet and Face-to-face Cognitive Behavioral Therapy for Postnatal Depression Compared With Treatment as Usual: Randomized Controlled Trial of MumMoodBooster. J Med Internet Res, 23(12), e17185. https://doi.org/10.2196/17185

Montero-Marín, J., Araya, R., Pérez-Yus, M. C., Mayoral, F., Gili, M., Botella, C., . . . López-Del-Hoyo, Y. (2016). An internet-based intervention for depression in primary Care in Spain: a randomized controlled trial. Journal of medical Internet research, 18(8), e231.

Mueller-Weinitschke C, Bengel J, Baumeister H, Krämer LV. Effects of a Web-Based Behavioral Activation Intervention on Depressive Symptoms, Activation, Motivation, and Volition: Results of a Randomized Controlled Trial. Psychotherapy and Psychosomatics. 2023.

Newby, J., Robins, L., Wilhelm, K., Smith, J., Fletcher, T., Gillis, I., . . . Andrews, G. (2017). Web-Based Cognitive Behavior Therapy for Depression in People With Diabetes Mellitus: a Randomized Controlled Trial. Journal of medical Internet research, 19(5), e157.

Nyström, M., Stenling, A., Sjöström, E., Neely, G., Lindner, P., HassmÈn, P., . . . Carlbring, P. (2017). Behavioral activation versus physical activity via the internet: a randomized controlled trial. Journal of Affective Disorders, 215, 85-93.

Oehler C, Görges F, Rogalla M, Rummel-Kluge C, Hegerl U. Efficacy of a Guided Web-Based Self-Management Intervention for Depression or Dysthymia: Randomized Controlled Trial With a 12-Month Follow-Up Using an Active Control Condition. J Med Internet Res. 2020;22(7):e15361.

Perini S, Titov N, Andrews G. Clinician-assisted Internet-based treatment is effective for depression: Randomized controlled trial. Australian and New Zealand Journal of Psychiatry. 2009;43(6):571-8.

Pihlaja S, Lahti J, Lipsanen JO, et al. Scheduled Telephone Support for Internet Cognitive Behavioral Therapy for Depression in Patients at Risk for Dropout: Pragmatic Randomized Controlled Trial. J Med Internet Res. 2020;22(7):e15732.

Raevuori, A., Vahlberg, T., Korhonen, T., Hilgert, O., Aittakumpu-Hyden, R., & Forman-Hoffman, V. (2021). A therapist-guided smartphone app for major depression in young adults: A randomized clinical trial. J Affect Disord, 286, 228-238. doi:10.1016/j.jad.2021.02.007

Reins, J. A., Boß, L., Lehr, D., Berking, M., & Ebert, D. D. (2019). The more I got, the less I need? Efficacy of Internet-based guided self-help compared to online psychoeducation for major depressive disorder. Journal of, 246, 695-705. doi:10.1016/j.jad.2018.12.065

Ritvo, P., et al. (2021). "Online Mindfulness-Based Cognitive Behavioral Therapy Intervention for Youth With Major Depressive Disorders: Randomized Controlled Trial." J Med Internet Res 23(3): e24380.

Schlicker S, Baumeister H, Buntrock C, et al. A web- And mobile-based intervention for comorbid, recurrent depression in patients with chronic back pain on sick leave (get.back): Pilot randomized controlled trial on feasibility, user satisfaction, and effectiveness. JMIR Mental Health. 2020;7(4).

Selmi PM, Klein MH, Greist JH, Sorrell SP, Erdman HP. Computer-administered cognitive-behavioral therapy for depression. American Journal of Psychiatry. 1990;147(1):51-6.

Titov N, Andrews G, Davies M, McIntyre K, Robinson E, Solley K. Internet treatment for depression: A randomized controlled trial comparing clinician vs. technician assistance. PLoS One. 2010;5(6):e10939.

Tønning, M. L., et al. (2021). "The effect of smartphone-based monitoring and treatment on the rate and duration of psychiatric readmission in patients with unipolar depressive disorder: The RADMIS randomized controlled trial." J Affect Disord 282: 354-363.

Tulbure BT, Andersson G, Salagean N, Pearce M, Koenig HG. Religious versus Conventional Internet-based Cognitive Behavioral Therapy for Depression. J Relig Health 2018; 57(5): 1634-48.

Vernmark K, Lenndin J, Bjärehed J, Carlsson M, Karlsson J, Oberg J, et al. Internet administered guided self-help versus individualized e-mail therapy: A randomized trial of two versions of CBT for major depression. Behaviour Research and Therapy. 2010;48(5):368-76.

Wright JH, Wright AS, Albano AM, Basco MR, Goldsmith LJ, Raffield T, et al. Computer-assisted cognitive therapy for depression: Maintaining efficacy while reducing therapist time. American Journal of Psychiatry. 2005;162(6):1158-64.

Gaudiano BA, Davis CH, Miller IW, Uebelacker L. Pilot randomized controlled trial of a video self-help intervention for depression based on acceptance and commitment therapy: Feasibility and acceptability. Clinical psychology & psychotherapy. 2020;27(3):396-407.

Gili M, Castro A, García-Palacios A, et al. Efficacy of Three Low-Intensity, Internet-Based Psychological Interventions for the Treatment of Depression in Primary Care: Randomized Controlled Trial. J Med Internet Res. 2020;22(6):e15845.

Hur, J. W., Kim, B., Park, D., & Choi, S. W. A Scenario-Based Cognitive Behavioral Therapy Mobile App to Reduce Dysfunctional Beliefs in Individuals with Depression: A Randomized Controlled Trial. Telemed J E Health, 24(9), 710-716.

Krämer, R., Köhne-Voll, , L., Schumacher, A., & Köhler, S. Efficacy of an online intervention for treatment of depressive disorders: a three-arm randomized controlled trial comparing guided and unguided self-help with waitlist control. JMIR Form Res.

Levin W, Campbell DR, McGovern KB, Gau JM, Kosty DB, Seeley JR, et al. A computer-assisted depression intervention in primary care. Psychological Medicine. 2011;41(7):1373-83.

Lin, Z., et al. (2023). "The Effect of Internet-Based Cognitive Behavioral Therapy on Major Depressive Disorder: Randomized Controlled Trial." Journal of Medical Internet Research 25(1).

Lobner M, Pabst A, Stein J, et al. Computerized cognitive behavior therapy for patients with mild to moderately severe depression in primary care: A pragmatic cluster randomized controlled trial (@ktiv). J Affect Disord 2018-238: 317-26.

Meyer, B., Weiss, M., Holtkamp, M., Arnold, S., Brückner, K., Schröder, J., . . . Nestoriuc, Y. (2019). Effects of an epilepsy-specific Internet intervention (Emyna) on depression: Results of the ENCODE randomized controlled trial. Epilepsia, 60(4), 656-668. doi:10.1111/epi.14673

Mohr DC, Duffecy J, Ho J, Kwasny M, Cai X, Burns MN, et al. A randomized controlled trial evaluating a manualized TeleCoaching protocol for improving adherence to a web-based intervention for the treatment of depression. PLoS One. 2013;8(8):e70086.

O'Moore K A, Newby JM, Andrews G, et al. Internet Cognitive-Behavioral Therapy for Depression in Older Adults With Knee Osteoarthritis: A Randomized Controlled Trial. Arthritis Care Res (Hoboken) 2018-70(1): 61-70.

Rosso, I. M., Killgore, W. D., Olson, E. A., Webb, C. A., Fukunaga, R., Auerbach, R. P., Gogel, H., Buchholz, J. L., & Rauch, S. L. Internet-based cognitive behavior therapy for major depressive disorder: A randomized controlled trial. Depress Anxiety, 34(3), 236-245. https://www.ncbi.nlm.nih.gov/pmc/articles/PMC5540163/pdf/nihms884093.pdf

Sandoval, L., Buckey, J., Ainslie, R., Tombari, M., Stone, W., & Hegel, M. (2017). Randomized Controlled Trial of a Computerized Interactive Media-Based Problem Solving Treatment for Depression. Behavior Therapy, 48(3), 413-425.

Smith, J., Newby, J. M., Burston, N., Murphy, M. J., Michael, S., Mackenzie, A., . . . Andrews, G. (2017). Help from home for depression: A randomised controlled trial comparing internet-delivered cognitive behaviour therapy with bibliotherapy for depression. Internet Interventions, 9, 25-37.

Williams AD, Blackwell SE, Mackenzie A, Holmes EA, Andrews G. Combining imagination and reason in the treatment of depression: A randomized controlled trial of internet-based cognitive-bias modification and internet-CBT for depression. Journal of Consulting and Clinical Psychology. 2013;81(5):793-9.

O'Mahen, H. A., Richards, D. A., Woodford, J., Wilkinson, E., McGinley, J., Taylor, R. S., & Warren, F. C. Netmums: a phase II randomized controlled trial of a guided Internet behavioural activation treatment for postpartum depression. Psychol Med, 44(8), 1675-1689. https://www.cambridge.org/core/services/aop-cambridge-core/content/view/8B6CB69C8AAC810B47184FC5FC69ADD9/S0033291713002092a.pdf/div-class-title-netmums-a-phase-ii-randomized-controlled-trial-of-a-guided-internet-behavioural-activation-treatment-for-postpartum-depression-div.pdf

Weise, C., Kaiser, G., a, C., Kues, J. N., Andersson, G., Strahler, J., & Kleinstäuber, M. (2019). Internet-Based Cognitive-Behavioural Intervention for Women with Premenstrual Dysphoric Disorder: A Randomized Controlled Trial. Psychother Psychosom, 88(1), 16-29. https://www.karger.com/Article/Abstract/496237

Insomnia

Bernstein AM, Allexandre D, Bena J, et al. "Go! to Sleep": A Web-Based Therapy for Insomnia. Telemed J E Health. 2017;23(7):590-599

Chan CS, Wong CYF, Yu BYM, Hui VKY, Ho FYY, Cuijpers P. Treating depression with a smartphonedelivered self-help cognitive behavioral therapy for insomnia: a parallel-group randomized controlled trial. Psychological Medicine. 2021:1-15

Cheng P, Kalmbach DA, Tallent G, Joseph CL, Espie CA, Drake CL. Depression prevention via digital cognitive behavioral therapy for insomnia: a randomized controlled trial. Sleep. 2019;42(10):zsz150.

Christensen, H., Batterham, P. J., Gosling, J. A., Ritterband, L. M., Griffiths, K. M., Thorndike, F. P., ... & Mackinnon, A. J. (2016). Effectiveness of an online insomnia program (SHUTi) for prevention of depressive episodes (the GoodNight Study): a randomised controlled trial. The Lancet Psychiatry, 3(4), 333-341.

Dekker K, Benjamins JS, Maksimovic T, et al. Combined Internet-Based Cognitive-Behavioral and Chronobiological Intervention for Insomnia: A Randomized Controlled Trial. Psychother Psychosom. 2020;89(2):117-118.

Espie CA, Kyle SD, Williams C, et al. A randomized, placebo-controlled trial of online cognitive behavioral therapy for chronic insomnia disorder delivered via an automated media-rich web application. Sleep. 2012;35(6):769-781.

Espie CA, Emsley R, Kyle SD, et al. Effect of Digital Cognitive Behavioral Therapy for Insomnia on Health, Psychological Well-being, and Sleep-Related Quality of Life: A Randomized Clinical Trial. JAMA Psychiatry. 2019;76(1):21-30.

Felder JN, Epel ES, Neuhaus J, Krystal AD, Prather AA. Efficacy of Digital Cognitive Behavioral Therapy for the Treatment of Insomnia Symptoms Among Pregnant Women: A Randomized Clinical Trial [published correction appears in JAMA Psychiatry. 2020 Jul 1;77(7):768]. JAMA Psychiatry. 2020;77(5):484-492.

Gieselmann A, Pietrowsky R. The effects of brief chat-based and face-to-face psychotherapy for insomnia: a randomized waiting list controlled trial. Sleep Med. 2019;61:63-72.

Hagatun S, Vedaa Ø, Nordgreen T, et al. The Short-Term Efficacy of an Unguided Internet-Based Cognitive-Behavioral Therapy for Insomnia: A Randomized Controlled Trial With a Six-Month Nonrandomized Follow-Up. Behav Sleep Med. 2019;17(2):137-155.

Hall DL, Arditte Hall KA, Gorman MJ, et al. The Survivorship Sleep Program (SSP): A synchronous, virtual cognitive behavioral therapy for insomnia pilot program among cancer survivors. Cancer. 2022;128(7):1532-1544.

Horsch CH, Lancee J, Griffioen-Both F, et al. Mobile Phone-Delivered Cognitive Behavioral Therapy for Insomnia: A Randomized Waitlist Controlled Trial. J Med Internet Res. 2017;19(4):e70.

Krieger T, Urech A, Duss SB, et al. A randomized controlled trial comparing guided internet-based multicomponent treatment and internet-based guided sleep restriction treatment to care as usual in insomnia.Sleep Med. 2019;62:43-52.

Kyle SD, Hurry MED, Emsley R, et al. The effects of digital cognitive behavioral therapy for insomnia on cognitive function: a randomized controlled trial. Sleep. 2020;43(9):zsaa034.

Lancee J, van den Bout J, van Straten A, Spoormaker VI. Internet-delivered or mailed self-help treatment for insomnia?: a randomized waiting-list controlled trial. Behav Res Ther. 2012;50(1):22-29.

Lancee J, Eisma MC, van Straten A, Kamphuis JH. Sleep-Related Safety Behaviors and Dysfunctional Beliefs Mediate the Efficacy of Online CBT for Insomnia: A Randomized Controlled Trial. Cogn Behav Ther. 2015;44(5):406-422.

Lancee J, van Straten A, Morina N, Kaldo V, Kamphuis JH. Guided Online or Face-to-Face Cognitive Behavioral Treatment for Insomnia: A Randomized Wait-List Controlled Trial. Sleep. 2016;39(1):183-191.

Leerssen J, Lakbila-Kamal O, Dekkers LMS, et al. Treating Insomnia with High Risk of Depression Using Therapist-Guided Digital Cognitive, Behavioral, and Circadian Rhythm Support Interventions to Prevent Worsening of Depressive Symptoms: A Randomized Controlled Trial. Psychother Psychosom. 2022;91(3):168-179.

Lorenz N, Heim E, Roetger A, Birrer E, Maercker A. Randomized Controlled Trial to Test the Efficacy of an Unguided Online Intervention with Automated Feedback for the Treatment of Insomnia. Behav Cogn Psychother. 2019;47(3):287-302.

Nazem, S., Barnes, S. M., Forster, J. E., Hostetter, T. A., Monteith, L. L., Kramer, E. B., ... & Brenner, L. A. (2023). Efficacy of an internet-delivered intervention for improving insomnia severity and functioning in veterans: randomized controlled trial. JMIR Mental Health, 10, e50516.

Rajabi Majd N, Broström A, Ulander M, et al. Efficacy of a Theory-Based Cognitive Behavioral Technique App-Based Intervention for Patients With Insomnia: Randomized Controlled Trial. J Med Internet Res. 2020;22(4):e15841.

Pillai V, Anderson JR, Cheng P, et al. The Anxiolytic Effects of Cognitive Behavior Therapy for Insomnia: Preliminary Results from a Web-delivered Protocol. J Sleep Med Disord. 2015;2(2):1017.

Ritterband LM, Thorndike FP, Gonder-Frederick LA, et al. Efficacy of an Internet-based behavioral intervention for adults with insomnia. Arch Gen Psychiatry. 2009;66(7):692-698.

Ritterband LM, Bailey ET, Thorndike FP, Lord HR, Farrell-Carnahan L, Baum LD. Initial evaluation of an Internet intervention to improve the sleep of cancer survivors with insomnia. Psychooncology.

Sato D, Yoshinaga N, Nagai E, Nagai K, Shimizu E. Effectiveness of Internet-Delivered Computerized Cognitive Behavioral Therapy for Patients With Insomnia Who Remain Symptomatic Following Pharmacotherapy: Randomized Controlled Exploratory Trial. J Med Internet Res. 2019;21(4):e12686.

Schuffelen J, Maurer LF, Lorenz N, Rötger A, Pietrowsky R, Gieselmann A. The clinical effects of digital cognitive behavioral therapy for insomnia in a heterogenous study sample: Results from a randomized controlled trial [published online ahead of print, 2023 Jul 10]. Sleep. 2023;zsad184. doi:10.1093/sleep/zsad184

Siebmanns S, Johansson P, Ulander M, Johansson L, Andersson G, Broström A. The effect of nurse-led Internet-based cognitive behavioural therapy for insomnia on patients with cardiovascular disease: A randomized controlled trial with 6-month follow-up. Nurs Open. 2021;8(4):1755-1768.

Støre SJ, Tillfors M, Wästlund E, Angelhoff C, Andersson G, Norell-Clarke A. The effects of a sleep robot intervention on sleep, depression and anxiety in adults with insomnia-A randomized waitlist-controlled trial. J Sleep Res. 2023;32(3):e13758. doi:10.1111/jsr.13758

Ström L, Pettersson R, Andersson G. Internet-based treatment for insomnia: a controlled evaluation. J Consult Clin Psychol. 2004;72(1):113-120.

Sunnhed R, Hesser H, Andersson G, et al. Comparing internet-delivered cognitive therapy and behavior therapy with telephone support for insomnia disorder: a randomized controlled trial. Sleep. 2020;43(2):zsz245.

van der Zweerde T, van Straten A, Effting M, Kyle SD, Lancee J. Does online insomnia treatment reduce depressive symptoms? A randomized controlled trial in individuals with both insomnia and depressive symptoms. Psychol Med. 2019;49(3):501-509.

Van der Zweerde T, Lancee J, Slottje P, Bosmans JE, Van Someren EJW, van Straten A. Nurse-Guided Internet-Delivered Cognitive Behavioral Therapy for Insomnia in General Practice: Results from a Pragmatic Randomized Clinical Trial. Psychother Psychosom. 2020;89(3):174-184.

van Straten A, Emmelkamp J, de Wit J, et al. Guided Internet-delivered cognitive behavioural treatment for insomnia: a randomized trial. Psychol Med. 2014;44(7):1521-1532.

Vincent N, Lewycky S. Logging on for better sleep: RCT of the effectiveness of online treatment for insomnia. Sleep. 2009;32(6):807-815.

Watanabe Y, Kuroki T, Ichikawa D, Ozone M, Uchimura N, Ueno T. Effect of smartphone-based cognitive behavioral therapy app on insomnia: a randomized, double-blind study. Sleep. 2023;46(3):zsac270. doi:10.1093/sleep/zsac270

Wiklund T, Molander P, Lindner P, Andersson G, Gerdle B, Dragioti E. Internet-Delivered Cognitive Behavioral Therapy for Insomnia Comorbid With Chronic Pain: Randomized Controlled Trial. J Med Internet Res. 2022;24(4):e29258.

Zhou K, Kong J, Wan Y, Zhang X, Liu X, Qu B, Wang B, Xue R. Positive impacts of e-aid cognitive behavioural therapy on the sleep quality and mood of nurses on site during the COVID-19 pandemic. Sleep Breath. 2022 Jan 8:1–5.

Social Anxiety Disorder

Anderson, P. L., Price, M., Edwards, S. M., Obasaju, M. A., Schmertz, S. K., Zimand, E., & Calamaras, M. R. (2013). Virtual reality exposure therapy for social anxiety disorder: a randomized controlled trial. J Consult Clin Psychol, 81(5), 751-760. https://doi.org/10.1037/a0033559

Andersson, G., Carlbring, P., Holmstrom, A., Sparthan, E., Furmark, T., Nilsson-Ihrfelt, E., Buhrman, M., & Ekselius, L. (2006). Internet-based self-help with therapist feedback and in vivo group exposure for social phobia: a

randomized controlled trial. J Consult Clin Psychol, 74(4), 677-686. https://doi.org/10.1037/0022-006x.74.4.677

Andersson, G., Carlbring, P., & Furmark, T. (2012). Therapist experience and knowledge acquisition in internet-delivered CBT for social anxiety disorder: a randomized controlled trial. PLoS One, 7(5), e37411. https://doi.org/10.1371/journal.pone.0037411

Berger, T., Hohl, E., & Caspar, F. (2009). Internet-based treatment for social phobia: a randomized controlled trial. J Clin Psychol, 65(10), 1021-1035. https://doi.org/10.1002/jclp.20603

Botella, C., Gallego, M. J., Garcia-Palacios, A., Guillen, V., Baños, R. M., Quero, S., & Alcañiz, M. (2010). An Internet-based self-help treatment for fear of public speaking: a controlled trial. Cyberpsychol Behav Soc Netw, 13(4), 407-421. https://doi.org/10.1089/cyber.2009.0224

Bouchard, S., Dumoulin, S., Robillard, G., Guitard, T., Klinger, E., Forget, H., Loranger, C., & Roucaut, F. X. (2017). Virtual reality compared with in vivo exposure in the treatment of social anxiety disorder: a three-arm randomised controlled trial. Br J Psychiatry, 210(4), 276-283. https://doi.org/10.1192/bjp.bp.116.184234

Carlbring, P., Gunnarsdottir, M., Hedensjo, L., Andersson, G., Ekselius, L., & Furmark, T. (2007). Treatment of social phobia: randomised trial of internet-delivered cognitive-behavioural therapy with telephone support. Br J Psychiatry, 190, 123-128. https://doi.org/10.1192/bjp.bp.105.020107

Clark, D., Wild, J., Warnock-Parkes, E., Stott, R., Grey, N., Thew, G., & Ehlers, A. (2023). More than doubling the clinical benefit of each hour of therapist time: A randomised controlled trial of internet cognitive therapy for social anxiety disorder. Psychol Med, 53(11), 5022-5032. doi:10.1017/S0033291722002008

Furmark, T., Carlbring, P., Hedman, E., Sonnenstein, A., Clevberger, P., Bohman, B., Eriksson, A., Hallen, A., Frykman, M., Holmstrom, A., Sparthan, E., Tillfors, M., Ihrfelt, E. N., Spak, M., Eriksson, A., Ekselius, L., & Andersson, G. (2009). Guided and unguided self-help for social anxiety disorder: randomised controlled trial. Br J Psychiatry, 195(5), 440-447. https://doi.org/10.1192/bjp.bp.108.060996

Gallego, M. J., Gerardus Emmelkamp, P. M., van der Kooij, M., & Mees, H. (2011). The effects of a Dutch version of an Internet-based treatment program for fear of public speaking: A controlled study. Int J Clin Health Psychol, 11(3), 459-472. https://www.redalyc.org/pdf/337/33719289003.pdf

Ivanova, E., Lindner, P., Ly, K. H., Dahlin, M., Vernmark, K., Andersson, G., & Carlbring, P. (2016). Guided and unguided Acceptance and Commitment Therapy for social anxiety disorder and/or panic disorder provided via the Internet and a smartphone application: A randomized controlled trial. J Anxiety Disord, 44, 27-35. https://doi.org/10.1016/j.janxdis.2016.09.012

Johansson, R., Hesslow, T., Ljotsson, B., Jansson, A., Jonsson, L., Fardig, S., Karlsson, J., Hesser, H., Frederick, R. J., Lilliengren, P., Carlbring, P., & Andersson, G. (2017). Internet-based affect-focused psychodynamic therapy for social anxiety disorder: A randomized controlled trial with 2-year follow-up. Psychotherapy (Chic), 54(4), 351-360. https://doi.org/10.1037/pst0000147

Kählke, F., Berger, T., Schulz, A., Baumeister, H., Berking, M., Auerbach, R. P., Bruffaerts, R., Cuijpers, P., Kessler, R. C., & Ebert, D. D. (2019). Efficacy of an unguided internet-based self-help intervention for social anxiety disorder in university students: A randomized controlled trial. Int J Methods Psychiatr Res, 28(2), e1766. https://doi.org/10.1002/mpr.1766

Kampmann, I. L., Emmelkamp, P. M., Hartanto, D., Brinkman, W. P., Zijlstra, B. J., & Morina, N. (2016). Exposure to virtual social interactions in the treatment of social anxiety disorder: A randomized controlled trial. Behav Res Ther, 77, 147-156. https://doi.org/10.1016/j.brat.2015.12.016

Kim, H., Kim, B. H., Kim, M. K., Eom, H., & Kim, J. J. (2022). Alteration of resting-state functional connectivity network properties in patients with social anxiety disorder after virtual reality-based self-training [Article]. Frontiers in Psychiatry, 13. https://doi.org/10.3389/fpsyt.2022.959696

Price, M., & Anderson, P. L. (2011). The impact of cognitive behavioral therapy on post event processing among those with social anxiety disorder. Behav Res Ther, 49(2), 132-137. https://doi.org/10.1016/j.brat.2010.11.006

Robillard, G., Bouchard, S., Dumoulin, S., Guitard, T., & Klinger, E. (2010). Using virtual humans to alleviate social anxiety: preliminary report from a comparative outcome study. Stud Health Technol Inform, 154, 57-60. https://doi.org/10.3233/978-1-60750-561-7-57

Schulz, A., Stolz, T., Vincent, A., Krieger, T., Andersson, G., & Berger, T. (2016). A sorrow shared is a sorrow halved? A three-arm randomized controlled trial comparing internet-based clinician-guided individual versus

group treatment for social anxiety disorder. Behav Res Ther, 84, 14-26. https://doi.org/10.1016/j.brat.2016.07.001

Schwob, J. T., & Newman, M. G. (2023). Brief imaginal exposure exercises for social anxiety disorder: A randomized controlled trial of a self-help momentary intervention app. J Anxiety Disord, 98, 102749. https://doi.org/10.1016/j.janxdis.2023.102749

Stolz, T., Schulz, A., Krieger, T., Vincent, A., Urech, A., Moser, C., Westermann, S., & Berger, T. (2018). A mobile app for social anxiety disorder: A three-arm randomized controlled trial comparing mobile and PC-based guided self-help interventions. J Consult Clin Psychol, 86(6), 493-504. https://doi.org/10.1037/ccp0000301

Thew, G. R., Kwok, A. P. L., Lissillour Chan, M. H., Powell, C., Wild, J., Leung, P. W. L., & Clark, D. M. (2022). Internet-delivered cognitive therapy for social anxiety disorder in Hong Kong: A randomized controlled trial. Internet Interv, 28, 100539. https://doi.org/10.1016/j.invent.2022.100539

Titov, N., Andrews, G., Schwencke, G., Drobny, J., & Einstein, D. (2008a). Shyness 1: distance treatment of social phobia over the Internet. Aust N Z J Psychiatry, 42(7), 585-594. https://doi.org/10.1080/00048670802119762

Titov, N., Andrews, G., & Schwencke, G. (2008b). Shyness 2: treating social phobia online: replication and extension. Aust N Z J Psychiatry, 42(7), 595-605. https://doi.org/10.1080/00048670802119820

Titov, N., Andrews, G., Choi, I., Schwencke, G., & Mahoney, A. (2008c). Shyness 3: randomized controlled trial of guided versus unguided Internet-based CBT for social phobia. Aust N Z J Psychiatry, 42(12), 1030-1040. https://doi.org/10.1080/00048670802512107

Wang, H., Zhao, Q., Mu, W., Rodriguez, M., Qian, M., & Berger, T. (2020). The Effect of Shame on Patients With Social Anxiety Disorder in Internet-Based Cognitive Behavioral Therapy: Randomized Controlled Trial. JMIR Ment Health, 7(7), e15797. https://doi.org/10.2196/15797

Zainal, N. H., Chan, W. W., Saxena, A. P., Taylor, C. B., & Newman, M. G. (2021). Pilot randomized trial of self-guided virtual reality exposure therapy for social anxiety disorder. Behav Res Ther, 147, 103984. https://doi.org/10.1016/j.brat.2021.103984

Panic Disorder

Allen AR, Newby JM, Mackenzie A, Smith J, Boulton M, Loughnan SA, Andrews G. Internet cognitive-behavioural treatment for panic disorder: randomised controlled trial and evidence of effectiveness in primary care. BJPsych Open. 2016 Mar 24;2(2):154-162.

Berger, T., et al. (2017). "Effects of a transdiagnostic unguided Internet intervention ('velibra') for anxiety disorders in primary care: results of a randomized controlled trial." Psychol Med 47(1): 67-80.

Carlbring P, Bohman S, Brunt S, et al. Remote treatment of panic disorder: a randomized trial of internet-based cognitive behavior therapy supplemented with telephone calls. The American journal of psychiatry 2006;163(12):2119-25.

Carlbring P, Westling BE, Ljungstrand P, et al. Treatment of panic disorder via the Internet: A randomized trial of a self-help program. Behavior Therapy 2001;32(4):751-64.

Ciuca AM, Berger T, Crișan LG, et al. Internet-based treatment for panic disorder: A three-arm randomized controlled trial comparing guided (via real-time video sessions) with unguided self-help treatment and a waitlist control. PAXPD study results. Journal of Anxiety Disorders 2018;56:43-55.

Kenardy JA, Dow MG, Johnston DW, et al. A comparison of delivery methods of cognitive-behavioral therapy for panic disorder: an international multicenter trial. Journal of consulting and clinical psychology 2003;71(6):1068-75.

Klein B, Richards JC, Austin DW. Efficacy of internet therapy for panic disorder. Journal of behavior therapy and experimental psychiatry 2006;37(3):213-38.

Meyerbroeker K, Morina N, Kerkhof GA, et al. Virtual reality exposure therapy does not provide any additional value in agoraphobic patients: a randomized controlled trial. Psychotherapy and psychosomatics 2013;82(3):170-6.

Oromendia P, Orrego J, Bonillo A, et al. Internet-based self-help treatment for panic disorder: a randomized controlled trial comparing mandatory versus optional complementary psychological support. Cognitive behaviour therapy 2016;45(4):270-86.

Richards JC, Klein B, Austin DW. Internet cognitive behavioural therapy for panic disorder: does the inclusion of stress management information improve end-state functioning? Clinical psychologist 2006;10(1):2-15.

Ruwaard J, Broeksteeg J, Schrieken B, et al. Web-based therapist-assisted cognitive behavioral treatment of panic symptoms: a randomized controlled trial with a three-year follow-up. Journal of anxiety disorders 2010;24(4):387-96.

Silfvernagel K, Carlbring P, Kabo J, et al. Individually tailored internet-based treatment for young adults and adults with panic attacks: randomized controlled trial. Journal of medical Internet research 2012;14(3):e65.

Titov, N., et al. (2010). "Transdiagnostic Internet treatment for anxiety disorders: A randomized controlled trial." Behav Res Ther 48(9): 890-899.

van Ballegooijen W, Riper H, Klein B, et al. An Internet-based guided self-help intervention for panic symptoms: randomized controlled trial. Journal of medical internet research 2013;15(7):e154.

Wims E, Titov N, Andrews G, et al. Clinician-assisted Internet-based treatment is effective for panic: A randomized controlled trial. The Australian and New Zealand journal of psychiatry 2010;44(7):599-607.

Posttraumatic Stress Disorder

Allen AR, Smith J, Hobbs MJ, et al. Internet-delivered cognitive behaviour therapy for post-traumatic stress disorder: a randomised controlled trial and outcomes in routine care. Behav Cogn Psychother. 2022 Nov;50(6):649-55. doi: 10.1017/S1352465822000285. PMID: 35924312.

Ehlers A, Wild J, Warnock-Parkes E, et al. Therapist-assisted online psychological therapies differing in trauma focus for post-traumatic stress disorder (STOP-PTSD): a UK-based, single-blind, randomised controlled trial. Lancet Psychiatry. 2023 Aug;10(8):608-22. doi: 10.1016/S2215-0366(23)00181-5. PMID: 37479341.

Ehlers A, Wild J, Warnock-Parkes E, et al. Therapist-assisted online psychological therapies differing in trauma focus for post-traumatic stress disorder (STOP-PTSD): a UK-based, single-blind, randomised controlled trial. Lancet Psychiatry. 2023 Aug;10(8):608-22. doi: 10.1016/S2215-0366(23)00181-5. PMID: 37479341.

Ehlers A, Wild J, Warnock-Parkes E, et al. Therapist-assisted online psychological therapies differing in trauma focus for post-traumatic stress disorder (STOP-PTSD): a UK-based, single-blind, randomised controlled trial. Lancet Psychiatry. 2023 Aug;10(8):608-22. doi: 10.1016/S2215-0366(23)00181-5. PMID: 37479341.

Ehlers A, Wild J, Warnock-Parkes E, et al. Therapist-assisted online psychological therapies differing in trauma focus for post-traumatic stress disorder (STOP-PTSD): a UK-based, single-blind, randomised controlled trial. Lancet Psychiatry. 2023 Aug;10(8):608-22. doi: 10.1016/S2215-0366(23)00181-5. PMID: 37479341.

Ehlers A, Wild J, Warnock-Parkes E, et al. Therapist-assisted online psychological therapies differing in trauma focus for post-traumatic stress disorder (STOP-PTSD): a UK-based, single-blind, randomised controlled trial. Lancet Psychiatry. 2023 Aug;10(8):608-22. doi: 10.1016/S2215-0366(23)00181-5. PMID: 37479341.

Ehlers A, Wild J, Warnock-Parkes E, et al. Therapist-assisted online psychological therapies differing in trauma focus for post-traumatic stress disorder (STOP-PTSD): a UK-based, single-blind, randomised controlled trial. Lancet Psychiatry. 2023 Aug;10(8):608-22. doi: 10.1016/S2215-0366(23)00181-5. PMID: 37479341.

Engel CC, Litz B, Magruder KM, et al. Delivery of self training and education for stressful situations (DESTRESS-PC): a randomized trial of nurse assisted online self-management for PTSD in primary care. Gen Hosp Psychiatry. 2015 Jul-Aug;37(4):323-8. doi: 10.1016/j.genhosppsych.2015.04.007. PMID: 25929985.

Ivarsson D, Blom M, Hesser H, et al. Guided internet-delivered cognitive behavior therapy for post-traumatic stress disorder: a randomized controlled trial. Internet Interv. 2014 March;1(1):33-40. doi: 10.1016/j.invent.2014.03.002.

Knaevelsrud C, Böttche M, Pietrzak RH, et al. Efficacy and feasibility of a therapist-guided internet-based intervention for older persons with childhood traumatization: a randomized controlled trial. Am J Geriatr Psychiatry. 2017 Aug;25(8):878-88. doi: 10.1016/j.jagp.2017.02.024. PMID: 28365000.

Knaevelsrud C, Brand J, Lange A, et al. Web-based psychotherapy for posttraumatic stress disorder in war-traumatized Arab patients: randomized controlled trial. J Med Internet Res. 2015 Mar 20;17(3):e71. doi: 10.2196/jmir.3582. PMID: 25799024.

Krupnick JL, Green BL, Amdur RL, et al. An internet-based writing intervention for PTSD in veterans: a feasibility and pilot effectiveness trial. Psychol Trauma. 2017 Jul;9(4):461-70. doi: 10.1037/tra0000176. PMID: 27607767.

Kuhn ER, Kanuri N, Hoffman JE, et al. A randomized controlled trial of a smartphone app for posttraumatic stress disorder symptoms. J Consult Clin Psychol. 2017 Mar;85(3):267-73. doi: 10.1037/ccp0000163. PMID: 28221061.

Lehavot K, Millard SP, Thomas RM, Yantsides K, Upham M, Beckman K, Hamilton AB, Sadler A, Litz B, Simpson T. A randomized trial of an online, coach-assisted self-management PTSD intervention tailored for women veterans. J Consult Clin Psychol. 2021 Feb;89(2):134-142. doi: 10.1037/ccp0000556. PMID: 33705169; PMCID: PMC8238393.

Lewis CE, Farewell D, Groves V, et al. Internet-based guided self-help for posttraumatic stress disorder (PTSD): randomized controlled trial. Depress Anxiety. 2017 Jun;34(6):555-65. doi: 10.1002/da.22645. PMID: 28557299.

Littleton HL, Grills AE, Kline KD, et al. The From Survivor to Thriver program: RCT of an online therapist-facilitated program for rape-related PTSD. J Anxiety Disord. 2016 Oct;43:41-51. doi: 10.1016/j.janxdis.2016.07.010. PMID: 27513363.

Miller-Graff L, Ellis K, Hosny N. PTSD Coach Online–Arabic: a randomized controlled pilot trial to examine feasibility, acceptability, and preliminary effectiveness. J Trauma Stress. 2021;34(1):23-34. doi: 10.1002/jts.22621. PMID: 33159373.

Miner A, Kuhn E, Hoffman JE, et al. Feasibility, acceptability, and potential efficacy of the PTSD Coach app: a pilot randomized controlled trial with community trauma survivors. Psychol Traum. 2016 May;8(3):384-92. doi: 10.1037/tra0000092. PMID: 27046668.

Spence J, Titov N, Dear BF, et al. Randomized controlled trial of internet-delivered cognitive behavioral therapy for posttraumatic stress disorder. Depress Anxiety. 2011 Jul;28(7):541-50. doi: 10.1002/da.20835. PMID: 21721073.

Stein J, Vohringer M, Wagner B, et al. Exposure versus cognitive restructuring techniques in brief internet-based cognitive behavioral treatment for Arabic-speaking people with posttraumatic stress disorder: randomized clinical trial. JMIR Ment Health. 2023 Dec 13;10:e48689. doi: 10.2196/48689. PMID: 38090792.

Generalized Anxiety Disorder

Alavi, N., & Hirji, A. (2020). The Efficacy of PowerPoint-based CBT Delivered Through Email: Breaking the Barriers to Treatment for Generalized Anxiety Disorder. Journal of Psychiatric Practice, 26(2), 89-100. doi:10.1097/PRA.0000000000000455

Andersson, G., Paxling, B., Roch-Norlund, P., Ostman, G., Norgren, A., Almlov, J., . . . Silverberg, F. (2012). Internet-based psychodynamic versus cognitive behavioral guided self-help for generalized anxiety disorder: a randomized controlled trial. Psychother Psychosom, 81(6), 344-355. doi:10.1159/000339371

Berger, T., Urech, A., Krieger, T., Stolz, T., Schulz, A., Vincent, A., ... & Meyer, B. (2017). Effects of a transdiagnostic unguided Internet intervention ('velibra') for anxiety disorders in primary care: results of a randomized controlled trial. Psychological medicine, 47(1), 67-80.

Dahlin, M., Andersson, G., Magnusson, K., Johansson, T., Sjogren, J., Hakansson, A., . . . Carlbring, P. (2016). Internet-delivered acceptance-based behaviour therapy for generalized anxiety disorder: A randomized controlled trial. Behav Res Ther, 77, 86-95. doi:10.1016/j.brat.2015.12.007

Jones, S. L., Hadjistavropoulos, H. D., & Soucy, J. N. (2016). A randomized controlled trial of guided internet-delivered cognitive behaviour therapy for older adults with generalized anxiety. J Anxiety Disord, 37, 1-9. doi:10.1016/j.janxdis.2015.10.006

Lorian, C. N., Titov, N., & Grisham, J. R. (2012). Changes in risk-taking over the course of an internet-delivered cognitive behavioral therapy treatment for generalized anxiety disorder. J Anxiety Disord, 26(1), 140-149. doi:10.1016/j.janxdis.2011.10.003

Newman, M. G., Jacobson, N. C., Rackoff, G. N., Bell, M. J., & Taylor, C. B. (2020). A randomized controlled trial of a smartphone-based application for the treatment of anxiety. Psychother Res, 1-12. doi:10.1080/10503307.2020.1790688

Paxling, B., Almlov, J., Dahlin, M., Carlbring, P., Breitholtz, E., Eriksson, T., & Andersson, G. (2011). Guided internet-delivered cognitive behavior therapy for generalized anxiety disorder: a randomized controlled trial. Cogn Behav Ther, 40(3), 159-173. doi:10.1080/16506073.2011.576699

Robinson, E., Titov, N., Andrews, G., McIntyre, K., Schwencke, G., & Solley, K. (2010). Internet treatment for generalized anxiety disorder: a randomized controlled trial comparing clinician vs. technician assistance. PLoS One, 5(6), e10942. doi:10.1371/journal.pone.0010942

Roy, A., Hoge, E. A., Abrante, P., Druker, S., Liu, T., & Brewer, J. A. (2021). Clinical Efficacy and Psychological Mechanisms of an App-Based Digital Therapeutic for Generalized Anxiety Disorder: Randomized Controlled Trial. Journal of Medical Internet Research, 23(12). doi:10.2196/26987

Titov, N., Andrews, G., Robinson, E., Schwencke, G., Johnston, L., Solley, K., & Choi, I. (2009). Clinician-assisted Internet-based treatment is effective for generalized anxiety disorder: Randomized controlled trial. Australian and New Zealand Journal of Psychiatry, 43(10), 905-912. doi:10.1080/00048670903179269

Titov, N., Andrews, G., Johnston, L., Robinson, E., & Spence, J. (2010). Transdiagnostic Internet treatment for anxiety disorders: A randomized controlled trial. Behaviour research and therapy, 48(9), 890-899.

Zainal, N. H., & Newman, M. G. (2023). A randomized controlled trial of a 14-day mindfulness ecological momentary intervention (MEMI) for generalized anxiety disorder. Eur Psychiatry, 66(1), e12.

Obsessive-Compulsive Disorder

Andersson, E., Enander, J., Andrén, P., Hedman, E., Ljótsson, B., Hursti, T., . . . Rück, C. (2012). Internet-based cognitive behaviour therapy for obsessive-compulsive disorder: a randomized controlled trial. Psychol Med, 42(10), 2193-2203.

Greist, J. H., Marks, I. M., Baer, L., Kobak, K. A., Wenzel, K. W., Hirsch, M. J., . . . Clary, C. M. (2002). Behavior therapy for obses- sive-compulsive disorder guided by a computer or by a clinician compared with relaxation as a control. J Clin Psychiatry, 63(2), 138-145.

Hawley, L. L., Rector, N. A., & Richter, M. A. (2021). Technology supported mindfulness for obsessive compulsive disorder: the role of obsessive beliefs. *Journal of Anxiety Disorders*, *81*, 102405.

Kyrios, M., Ahern, C., Fassnacht, D. B., Nedeljkovic, M., Moulding, R., & Meyer, D. (2018). Therapist-Assisted Internet-Based Cogni- tive Behavioral Therapy Versus Progressive Relaxation in Obsessive-Compulsive Disorder: Randomized Controlled Trial. J Med Internet Res, 20(8), e242.

Mahoney, A. E., Mackenzie, A., Williams, A. D., Smith, J., & Andrews, G. (2014). Internet cognitive behavioural treatment for obsessive compulsive disorder: a randomised controlled trial. *Behaviour research and therapy*, *63*, 99-106.

Matsumoto, K., Hamatani, S., Makino, T., Takahashi, J., Suzuki, F., Ida, T., . . . Omori, I. M. (2022). Guided internet-based cognitive behavioral therapy for obsessive-compulsive disorder: A multicenter randomized controlled trial in Japan. Internet interventions, 28, 100515.

Wootton, B. M., Dear, B. F., Johnston, L., Terides, M. D., & Titov, N. (2013). Remote treatment of obsessive-compulsive disorder: A randomized controlled trial. Journal of Obsessive-Compulsive and Related Disorders, 2(4), 375-384.

Wu, Y., Li, X., Zhou, Y., Gao, R., Wang, K., Ye, H., ... & Fan, Q. (2023). Efficacy and cost-effectiveness analysis of internet-based cognitive behavioral therapy for obsessive-compulsive disorder: randomized controlled trial. *Journal of medical Internet research*, *25*, e41283.

Specific Phobia

Campos, D., Bretón-López, J., Botella, C., Mira, A., Castilla, D., Mor, S., . . . Quero, S. (2019). Efficacy of an internet-based exposure treatment for flying phobia (NO-FEAR Airlines) with and without therapist guidance: a randomized controlled trial. BMC Psychiatry, 19(1), 86. doi:10.1186/s12888-019-2060-4

Garcia-Palacios, A., Hoffman, H., Carlin, A., Furness, T. A., 3rd, & Botella, C. (2002). Virtual reality in the treatment of spider phobia: a controlled study. Behav Res Ther, 40(9), 983-993. doi:10.1016/s0005-7967(01)00068-7

Gujjar, K. R., van Wijk, A., Kumar, R., & de Jongh, A. (2019). Efficacy of virtual reality exposure therapy for the treatment of dental phobia in adults: A randomized controlled trial. J Anxiety Disord, 62, 100-108. doi:10.1016/j.janxdis.2018.12.001

Jiang, M. Y. W., Upton, E., & Newby, J. M. (2020). A randomised wait-list controlled pilot trial of one-session virtual reality exposure therapy for blood-injection-injury phobias. J Affect Disord, 276, 636-645. doi:10.1016/j.jad.2020.07.076

Krijn, M., Emmelkamp, P. M., Biemond, R., de Wilde de Ligny, C., Schuemie, M. J., & van der Mast, C. A. (2004). Treatment of acrophobia in virtual reality: the role of immersion and presence. Behav Res Ther, 42(2), 229-239. doi:10.1016/s0005-7967(03)00139-6

S5. *Effects of digital interventions for eight mental disorders (sensitivity analyses)*

	k	*g*	95%-CI	I^2	95%-CI	95%-PI	NNT
Depression							
Overall							
Combined	60	0.62	[0.52; 0.72]	82.76	[78.42; 86.23]	[-0.08; 1.32]	4.72
Influence analysis	59	0.6	[0.5; 0.7]	78.14	[72.15; 82.84]	[-0.05; 1.24]	4.91
Limit meta-analysis	60	0.44	[0.24; 0.64]	80.05	-	[-0.28; 1.16]	6.91
One ES/study (highest)	49	0.7	[0.55; 0.84]	74.79	[66.76; 80.88]	[-0.08; 1.48]	4.12
One ES/study (lowest)	49	0.47	[0.37; 0.58]	70.73	[60.97; 78.06]	[-0.14; 1.09]	6.38
Low risk of bias only	10	0.57	[0.33; 0.81]	71.01	[44.7; 84.81]	[-0.13; 1.27]	5.21
Selection model	60	0.61	[0.5; 0.72]	79.91	[70.58; 87.41]	[-0.1; 1.31]	4.82
Three-Level Model (CHE)	150	0.62	[0.49; 0.74]	80.8	-	[-0.18; 1.41]	4.74
Trim-and-fill method	81	0.39	[0.27; 0.51]	89.66	[87.79; 91.25]	[-0.61; 1.38]	8.03
Guided interventions							
Combined	41	0.62	[0.49; 0.75]	82.35	[76.78; 86.58]	[-0.11; 1.35]	4.7
Influence analysis	40	0.59	[0.47; 0.71]	73.63	[64.05; 80.66]	[-0.07; 1.25]	4.99
Limit meta-analysis	41	0.41	[0.15; 0.67]	87.27	-	[-0.36; 1.18]	7.52
One ES/study (highest)	36	0.74	[0.55; 0.93]	87.91	[84.28; 90.71]	[-0.34; 1.81]	3.87
One ES/study (lowest)	36	0.5	[0.37; 0.64]	74.8	[65.19; 81.76]	[-0.18; 1.19]	5.96
Low risk of bias only	8	0.57	[0.28; 0.87]	73.82	[46.83; 87.11]	[-0.24; 1.38]	5.14
Selection model	41	0.61	[0.47; 0.74]	79.01	[67.56; 88.06]	[-0.12; 1.34]	4.82
Three-Level Model (CHE)	82	0.62	[0.47; 0.78]	79.1	-	[-0.21; 1.46]	4.67
Trim-and-fill method	55	0.4	[0.25; 0.54]	88.98	[86.45; 91.03]	[-0.62; 1.41]	7.83
Unguided interventions							
Combined	19	0.62	[0.43; 0.81]	84.42	[76.93; 89.48]	[-0.1; 1.33]	4.74
Influence analysis	19	0.62	[0.43; 0.81]	84.42	[76.93; 89.48]	[-0.1; 1.33]	4.74
Limit meta-analysis	19	0.49	[0.18; 0.81]	70.44	-	[-0.27; 1.26]	6.09
One ES/study (highest)	18	0.82	[0.5; 1.14]	69.88	[51.23; 81.4]	[-0.17; 1.81]	3.43
One ES/study (lowest)	18	0.51	[0.3; 0.71]	76.21	[62.57; 84.88]	[-0.21; 1.23]	5.89
Low risk of bias only	2	0.56	[-2.79; 3.91]	76.48	[0; 94.65]	[-; -]	5.34
Selection model	19	0.61	[0.43; 0.79]	79.61	[60.19; 92.24]	[-0.05; 1.27]	4.82
Three-Level Model (CHE)	68	0.72	[0.45; 0.98]	88.7	-	[-0.31; 1.75]	3.99
Trim-and-fill method	27	0.35	[0.12; 0.57]	91.24	[88.44; 93.36]	[-0.73; 1.42]	9.08
Insomnia							
Overall							
Combined	40	-0.94	[-1.07; -0.81]	72.68	[62.64; 80.03]	[-1.61; -0.27]	2.93
Influence analysis	40	-0.94	[-1.07; -0.81]	72.68	[62.64; 80.03]	[-1.61; -0.27]	2.93
Limit meta-analysis	40	-0.87	[-1.06; -0.68]	20.98	-	[-1.56; -0.18]	3.22
One ES/study (highest)	37	-0.91	[-1.04; -0.77]	72.62	[62.07; 80.23]	[-1.58; -0.24]	3.07
One ES/study (lowest)	37	-0.96	[-1.1; -0.82]	73.6	[63.54; 80.88]	[-1.66; -0.27]	2.87
Low risk of bias only	2	-0.99	[-3.19; 1.21]	73.13	[0; 93.95]	[-; -]	2.79
Selection model	40	-0.92	[-1.07; -0.77]	81.59	[66.74; 91.29]	[-1.6; -0.25]	3.02
Three-Level Model (CHE)	43	-0.94	[-1.07; -0.82]	80.9	-	[-1.63; -0.26]	2.93
Trim-and-fill method	41	-0.93	[-1.06; -0.79]	73.45	[63.91; 80.46]	[-1.63; -0.23]	2.99
Guided interventions							
Combined	16	-1.05	[-1.26; -0.84]	61.14	[32.99; 77.46]	[-1.71; -0.39]	2.61
Influence analysis	16	-1.05	[-1.26; -0.84]	61.14	[32.99; 77.46]	[-1.71; -0.39]	2.61
Limit meta-analysis	16	-0.92	[-1.30; -0.53]	49.83	-	[-1.67; -0.16]	3.03
One ES/study (highest)	13	-0.97	[-1.23; -0.72]	61.61	[29.83; 79]	[-1.7; -0.25]	2.83
One ES/study (lowest)	13	-1.13	[-1.36; -0.89]	58.31	[22.95; 77.45]	[-1.79; -0.46]	2.42
Low risk of bias only	18	-1.03	[-1.24; -0.83]	63.95	[40.32; 78.22]	[-1.75; -0.32]	2.66
Selection model	16	-1.04	[-1.25; -0.84]	59.37	[21.2; 86.22]	[-1.63; -0.45]	2.63
Three-Level Model (CHE)	18	-1.05	[-1.29; -0.82]	66.2	-	[-1.79; -0.32]	2.6
Trim-and-fill method	17	-1.03	[-1.24; -0.82]	61.46	[34.7; 77.25]	[-1.7; -0.36]	2.67
Unguided interventions							
Combined	24	-0.88	[-1.06; -0.71]	76.88	[65.91; 84.31]	[-1.6; -0.16]	3.17
Influence analysis	24	-0.88	[-1.06; -0.71]	76.88	[65.91; 84.31]	[-1.6; -0.16]	3.17
Limit meta-analysis	24	-0.87	[-1.1; -0.64]	18.34	-	[-1.61; -0.13]	3.22
One ES/study (highest)	24	-0.88	[-1.05; -0.7]	76.87	[65.9; 84.31]	[-1.59; -0.16]	3.19
One ES/study (lowest)	24	-0.89	[-1.06; -0.71]	77.12	[66.31; 84.46]	[-1.62; -0.16]	3.14
Low risk of bias only	2	-0.99	[-3.19; 1.21]	73.13	[0; 93.95]	[-; -]	2.79
Selection model	24	-0.86	[-1.06; -0.66]	85.99	[69.39; 95.15]	[-1.56; -0.16]	3.25
Three-Level Model (CHE)	25	-0.88	[-1.05; -0.72]	85.2	-	[-1.6; -0.17]	3.16

Trim-and-fill method	25	-0.92	[-1.11; -0.73]	78.73	[69.16; 85.33]	[-1.73; -0.11]	3.03
Social Anxiety Disorder							
Overall							
Combined	31	-0.79	[-0.92; -0.65]	56.43	[34.89; 70.84]	[-1.34; -0.23]	3.6
Influence analysis	30	-0.75	[-0.87; -0.64]	40.92	[8.5; 61.85]	[-1.13; -0.38]	3.79
Limit meta-analysis	31	-0.68	[-1.04; -0.33]	64.85	-	[-1.34; -0.03]	4.21
One ES/study (highest)	26	-0.59	[-0.78; -0.4]	63.6	[44.62; 76.08]	[-1.35; 0.17]	4.96
One ES/study (lowest)	26	-1.08	[-1.29; -0.87]	67.71	[51.49; 78.51]	[-1.95; -0.21]	2.53
Low RoB only	3	-1.22	[-3.25; 0.8]	88.54	[68.32; 95.85]	[-12.63; 10.19]	2.23
Selection model	31	-0.77	[-0.95; -0.6]	59.17	[18.47; 83.25]	[-1.32; -0.23]	3.67
Three-Level Model (CHE)	92	-0.84	[-0.99; -0.68]	70.9	-	[-1.63; -0.05]	3.35
Trim-and-fill method	31	-0.79	[-0.92; -0.65]	56.43	[34.89; 70.84]	[-1.34; -0.23]	3.6
Guided interventions							
Combined	22	-0.83	[-1; -0.67]	53.19	[24; 71.17]	[-1.36; -0.31]	3.37
Influence analysis	21	-0.78	[-0.9; -0.67]	23.69	[0; 55.2]	[-0.9; -0.66]	3.62
Limit meta-analysis	22	-0.56	[-0.98; -0.13]	62.17	-	[-1.23; 0.12]	5.34
One ES/study (highest)	20	-0.69	[-0.9; -0.48]	60.81	[36.17; 75.94]	[-1.42; 0.05]	4.19
One ES/study (lowest)	20	-1.05	[-1.28; -0.82]	62.48	[39.2; 76.84]	[-1.84; -0.26]	2.61
Low RoB only	3	-1.22	[-3.25; 0.8]	88.54	[68.32; 95.85]	[-12.63; 10.19]	2.23
Selection model	22	-0.82	[-1; -0.65]	52.82	[0; 85.63]	[-1.32; -0.33]	3.42
Three-Level Model (CHE)	65	-0.88	[-1.06; -0.7]	64.8	-	[-1.58; -0.19]	3.15
Trim-and-fill method	25	-0.75	[-0.94; -0.55]	67.42	[50.61; 78.51]	[-1.54; 0.04]	3.81
Unguided interventions							
Combined	9	-0.66	[-0.96; -0.37]	62.28	[22.21; 81.71]	[-1.44; 0.11]	4.37
Influence analysis	8	-0.57	[-0.69; -0.45]	0	[0; 67.58]	[-0.76; -0.39]	5.16
Limit meta-analysis	9	-0.87	[-1.5; -0.24]	65.41	-	[-1.89; 0.15]	3.21
One ES/study (highest)	9	-0.49	[-0.86; -0.12]	64.26	[26.9; 82.53]	[-1.41; 0.42]	6.14
One ES/study (lowest)	9	-0.99	[-1.44; -0.54]	76.5	[55.07; 87.71]	[-2.27; 0.29]	2.79
Low RoB only	27	-0.73	[-0.95; -0.5]	71.16	[57.51; 80.43]	[-1.7; 0.25]	3.92
Selection model	9	-0.65	[-1.03; -0.27]	61.78	[0; 90.93]	[-1.32; 0.02]	4.49
Three-Level Model (CHE)	27	-0.72	[-1.06; -0.37]	79.2	-	[-1.78; 0.35]	4
Trim-and-fill method	12	-0.78	[-1.04; -0.52]	63.89	[32.91; 80.56]	[-1.53; -0.03]	3.62
Posttraumatic Stress Disorder							
Overall							
Combined	17	0.62	[0.36; 0.89]	76.96	[63.4; 85.5]	[-0.35; 1.59]	4.69
Influence analysis	17	0.62	[0.36; 0.89]	76.96	[63.4; 85.5]	[-0.35; 1.59]	4.69
Limit meta-analysis	17	0.59	[0.16; 1.02]	58.66	-	[-0.45; 1.63]	4.98
One ES/study (highest)	15	0.57	[0.27; 0.88]	76.89	[62.13; 85.9]	[-0.47; 1.62]	5.14
One ES/study (lowest)	15	0.51	[0.25; 0.77]	68.56	[46.34; 81.58]	[-0.31; 1.34]	5.84
Low RoB only	2	1.14	[-2.27; 4.56]	74.39	[0; 94.22]	[-; -]	2.39
Selection model	17	0.58	[0.29; 0.87]	80.39	[57.88; 94.29]	[-0.38; 1.54]	5.05
Three-Level Model (CHE)	21	0.57	[0.28; 0.85]	78.1	-	[-0.39; 1.53]	5.2
Trim-and-fill method	20	0.78	[0.49; 1.06]	81.5	[72.37; 87.61]	[-0.4; 1.95]	3.65
Guided interventions							
Combined	14	0.72	[0.42; 1.02]	75.2	[58.22; 85.28]	[-0.27; 1.71]	3.97
Influence analysis	14	0.72	[0.42; 1.02]	75.2	[58.22; 85.28]	[-0.27; 1.71]	3.97
Limit meta-analysis	14	0.7	[0.24; 1.15]	51.6	-	[-0.36; 1.76]	4.13
One ES/study (highest)	12	0.68	[0.32; 1.04]	76.96	[59.89; 86.77]	[-0.46; 1.81]	4.24
One ES/study (lowest)	12	0.6	[0.29; 0.91]	68.26	[42.1; 82.6]	[-0.29; 1.5]	4.87
Low RoB only	2	1.14	[-2.27; 4.56]	74.39	[0; 94.22]	[-; -]	2.39
Selection model	14	0.7	[0.42; 0.99]	78.28	[51.36; 93.87]	[-0.2; 1.6]	4.09
Three-Level Model (CHE)	18	0.67	[0.34; 1.00]	78.7	-	[-0.33; 1.66]	4.33
Trim-and-fill method	18	0.98	[0.64; 1.31]	85.15	[77.89; 90.03]	[-0.38; 2.33]	2.83
Unguided interventions							
Combined	3	0.19	[-0.34; 0.73]	0	[0; 89.6]	[-1.54; 1.93]	17.03
Influence analysis	2	0.08	[-1.00; 1.17]	0	[-; -]	[-; -]	40.59
Limit meta-analysis	3	0.46	[-1.12; 2.04]	0	-	[-3; 3.93]	6.58
One ES/study (highest)	3	0.19	[-0.34; 0.73]	0	[0; 89.6]	[-1.54; 1.93]	17.03
One ES/study (lowest)	3	0.19	[-0.34; 0.73]	0	[0; 89.6]	[-1.54; 1.93]	17.03
Low RoB only	3	0.19	[-0.34; 0.73]	0	[0; 89.6]	[-1.54; 1.93]	17.03
Selection model	3	0.19	[-0.08; 0.46]	0.02	[0; 95.58]	[-0.08; 0.46]	17.48
Three-Level Model (CHE)	3	0.19	[-0.35; 0.74]	0	-	[-0.28; 0.67]	17.03
Trim-and-fill method	4	0.26	[-0.13; 0.64]	0	[0; 84.69]	[-0.28; 0.79]	12.67
Panic Disorder							

Overall							
Combined	18	1.06	[0.79; 1.33]	68.42	[48.57; 80.61]	[0.12; 2]	2.6
Influence analysis	18	1.06	[0.79; 1.33]	68.42	[48.57; 80.61]	[0.12; 2]	2.6
Limit meta-analysis	18	0.46	[-0.04; 0.96]	96.31	-	[-0.59; 1.5]	6.62
One ES/study (highest)	15	1.10	[0.78; 1.43]	73.22	[55.25; 83.97]	[0.03; 2.18]	2.48
One ES/study (lowest)	15	0.96	[0.67; 1.24]	66.28	[41.91; 80.43]	[0.05; 1.87]	2.88
Low RoB only	2	0.65	[-2.74; 4.04]	48.89	[-; -]	[-; -]	4.49
Selection model	18	1.05	[0.81; 1.3]	66.2	[37.02; 85.62]	[0.21; 1.89]	2.61
Three-Level Model (CHE)	18	1.05	[0.78; 1.32]	70.1	-	[0.07; 2.03]	2.61
Trim-and-fill method	25	0.75	[0.43; 1.08]	77.48	[67.16; 84.56]	[-0.64; 2.15]	3.77
Guided interventions							
Combined	16	1.13	[0.83; 1.42]	68.31	[46.77; 81.13]	[0.16; 2.09]	2.43
Influence analysis	16	1.13	[0.83; 1.42]	68.31	[46.77; 81.13]	[0.16; 2.09]	2.43
Limit meta-analysis	16	0.54	[-0.02; 1.1]	94.4	-	[-0.56; 1.64]	5.54
One ES/study (highest)	14	1.16	[0.83; 1.49]	71.59	[51.25; 83.45]	[0.11; 2.21]	2.36
One ES/study (lowest)	14	1.04	[0.75; 1.34]	66.26	[40.73; 80.8]	[0.13; 1.95]	2.63
Low RoB only1	1	0.97	[0.34; 1.61]	-	[-; -]	[-; -]	2.84
Selection model	16	1.12	[0.86; 1.39]	64.22	[33.24; 85.25]	[0.28; 1.97]	2.44
Three-Level Model (CHE)	16	1.12	[0.83; 1.41]	68.8	-	[0.12; 2.13]	2.43
Trim-and-fill method	22	0.82	[0.47; 1.17]	77.7	[66.67; 85.08]	[-0.61; 2.25]	3.43
Generalized Anxiety Disorder							
Overall							
Combined	13	-0.73	[-1.06; -0.39]	81.02	[68.55; 88.55]	[-1.87; 0.42]	3.94
Influence analysis	13	-0.73	[-1.06; -0.39]	81.02	[68.55; 88.55]	[-1.87; 0.42]	3.94
Limit meta-analysis	13	-0.75	[-1.70; 0.20]	89.98	-	[-2.26; 0.76]	3.8
One ES/study (highest)	12	-0.61	[-1.03; -0.20]	81.78	[69.32; 89.18]	[-1.99; 0.76]	4.77
One ES/study (lowest)	12	-0.98	[-1.35; -0.61]	75.63	[57.22; 86.11]	[-2.16; 0.2]	2.82
Only low risk of bias studies	5	-0.68	[-1.31; -0.06]	74.81	[37.81; 89.8]	[-2.23; 0.86]	4.21
Selection model	13	-0.96	[-1.3; -0.62]	73.05	[46.44; 89.39]	[-1.78; -0.13]	2.89
Three-Level Model (CHE)	41	-0.8	[-1.16; -0.45]	79.6	-	[-1.94; 0.34]	3.52
Trim-and-fill method	13	-0.73	[-1.06; -0.39]	81.02	[68.55; 88.55]	[-1.87; 0.42]	3.94
Guided interventions							
Combined	10	-0.71	[-1.12; -0.29]	82.84	[69.81; 90.24]	[-1.98; 0.57]	4.07
Influence analysis	10	-0.71	[-1.12; -0.29]	82.84	[69.81; 90.24]	[-1.98; 0.57]	4.07
Limit meta-analysis	10	-0.79	[-1.88; 0.29]	87.4	-	[-2.51; 0.92]	3.56
One ES/study (highest)	9	-0.57	[-1.12; -0.03]	84.34	[71.95; 91.26]	[-2.2; 1.06]	5.15
One ES/study (lowest)	9	-1.03	[-1.5; -0.55]	76.84	[55.81; 87.86]	[-2.37; 0.32]	2.68
Only low RoB studies	4	-0.77	[-1.62; 0.08]	75.09	[30.95; 91.01]	[-3.04; 1.5]	3.68
Selection model	10	-0.94	[-1.36; -0.52]	75.39	[46.88; 91.63]	[-1.85; -0.04]	2.94
Three-Level Model (CHE)	36	-0.80	[-1.25; -0.35]	81.2	-	[-2.03; 0.43]	3.52
Trim-and-fill method	11	-0.78	[-1.18; -0.37]	83.23	[71.41; 90.16]	[-2.08; 0.53]	3.64
Unguided interventions							
Combined	3	-0.79	[-2.24; 0.66]	81.37	[42.05; 94.01]	[-8.7; 7.12]	3.58
Influence analysis	2	-0.50	[-2.14; 1.13]	0	[-; -]	[-; -]	5.96
Limit meta-analysis	3	-0.36	[-2.95; 2.22]	98.86	-	[-6.47; 5.74]	8.59
One ES/study (highest)	3	-0.72	[-2.16; 0.72]	78.41	[30.57; 93.29]	[-8.47; 7.03]	3.97
One ES/study (lowest)	3	-0.85	[-2.32; 0.62]	78.39	[30.5; 93.28]	[-8.74; 7.04]	3.31
Only low RoB studies	1	-0.33	[-0.84; 0.19]	-	[-; -]	[-; -]	9.72
Selection model	3	-0.99	[-1.52; -0.47]	57.86	[0; 95.98]	[-1.75; -0.24]	2.78
Three-Level Model (CHE)	5	-0.79	[-2.22; 0.63]	81.8	-	[-2.52; 0.94]	3.56
Trim-and-fill method	3	-0.79	[-2.24; 0.66]	81.37	[42.05; 94.01]	[-8.7; 7.12]	3.58
**Obsessive-Compulsive Disorder**							
Overall							
Combined	9	-0.65	[-0.81; -0.48]	0	[0; 64.8]	[-0.83; -0.47]	4.49
Influence analysis	9	-0.65	[-0.81; -0.48]	0	[0; 64.8]	[-0.83; -0.47]	4.49
Limit meta-analysis	9	-0.63	[-1.15; -0.12]	0	-	[-1.24; -0.03]	4.59
One ES/study (highest)	9	-0.58	[-0.71; -0.45]	0	[0; 64.8]	[-0.77; -0.4]	5.05
One ES/study (lowest)	9	-0.72	[-0.98; -0.46]	40.49	[0; 72.59]	[-1.23; -0.2]	3.99
Selection model	9	-0.65	[-0.82; -0.48]	0	[0; 58.89]	[-0.82; -0.48]	4.45
Three-Level Model (CHE)	14	-0.68	[-0.86; -0.49]	33.3	-	[-1.11; -0.24]	4.27
Trim-and-fill method	9	-0.65	[-0.81; -0.48]	0	[0; 64.8]	[-0.83; -0.47]	4.49
Guided interventions							
Combined	5	-0.68	[-0.92; -0.44]	0	[0; 79.2]	[-0.99; -0.37]	4.22
Influence analysis	4	-0.78	[-1.08; -0.49]	0	[0; 84.69]	[-1.33; -0.23]	3.61
Limit meta-analysis	5	-0.37	[-0.98; 0.25]	0	-	[-1.24; 0.5]	8.47

One ES/study (highest)	5	-0.59	[-0.72; -0.45]	0	[0; 79.2]	[-0.91; -0.26]	5
One ES/study (lowest)	5	-0.85	[-1.34; -0.36]	57.59	[0; 84.25]	[-1.92; 0.23]	3.31
Selection model	-	-	[-; -]	-	-	[-; -]	-
Three-Level Model (CHE)	8	-0.77	[-1.06; -0.48]	52.5	-	[-1.45; -0.09]	3.68
Trim-and-fill method	7	-0.63	[-0.83; -0.43]	0	[0; 70.81]	[-0.87; -0.4]	4.6
Unguided interventions							
Combined	4	-0.59	[-1.00; -0.17]	10.71	[0; 86.33]	[-1.23; 0.05]	5.02
Influence analysis	2	-0.57	[-4.29; 3.07]	37.87	[-; -]	[-; -]	5.23
Limit meta-analysis	4	-1.04	[-1.83; -0.24]	3.72	-	[-2.35; 0.27]	2.65
One ES/study (highest)	4	-0.57	[-0.98; -0.17]	2.43	[0; 85.06]	[-1.2; 0.05]	5.16
One ES/study (lowest)	4	-0.6	[-1.01; -0.19]	1.84	[0; 84.97]	[-1.24; 0.04]	4.9
Selection model	4	-0.74	[-1.03; -0.45]	0	[0; 64.99]	[-1.03; -0.45]	3.84
Three-Level Model (CHE)	6	-0.59	[-1.05; -0.13]	6.9	-	[-0.97; -0.2]	5.02
Trim-and-fill method	5	-0.63	[-0.98; -0.28]	15.34	[0; 82.39]	[-1.04; -0.22]	4.62
Specific Phobias							
Overall							
Combined	7	1.13	[0.64; 1.62]	50.01	[0; 78.81]	[0.12; 2.14]	2.42
Influence analysis	6	1.00	[0.59; 1.41]	23.35	[0; 67.29]	[0.31; 1.69]	2.76
Limit meta-analysis	7	-0.26	[-1.41; 0.9]	71.93	-	[-1.93; 1.41]	12.54
One ES/study (highest)	5	1.28	[0.56; 2.01]	54.15	[0; 83.1]	[-0.25; 2.82]	2.12
One ES/study (lowest)	5	1.14	[0.3; 1.98]	64.9	[7.96; 86.62]	[-0.77; 3.05]	2.4
Only low RoB studies	3	0.96	[0.06; 1.87]	14.39	[0; 91.1]	[-2.44; 4.37]	2.87
Selection model	7	1.11	[0.76; 1.47]	37	[0; 85.71]	[0.47; 1.76]	2.46
Three-Level Model (CHE)	7	1.18	[0.52; 1.85]	56.6	-	[0.03; 2.33]	2.31
Trim-and-fill method	9	0.93	[0.37; 1.49]	64.17	[26.69; 82.49]	[-0.47; 2.33]	2.99
Guided interventions							
Combined	5	1.08	[0.3; 1.86]	60.46	[0; 85.19]	[-0.57; 2.73]	2.54
Influence analysis	4	0.87	[0.29; 1.44]	19.23	[0; 87.63]	[-0.15; 1.89]	3.22
Limit meta-analysis	5	-0.17	[-1.46; 1.11]	83.27	-	[-2.38; 2.04]	19.09
One ES/study (highest)	4	1.23	[0.19; 2.28]	61.2	[0; 87.01]	[-1.24; 3.7]	2.21
One ES/study (lowest)	4	1.09	[-0.11; 2.28]	68.81	[9.78; 89.22]	[-1.9; 4.07]	2.52
Only low RoB studies	2	0.9	[-3.33; 5.12]	48.5	[-; -]	[-; -]	3.1
Selection model	5	1.05	[0.59; 1.5]	46.93	[0; 93.77]	[0.26; 1.84]	2.62
Three-Level Model (CHE)	5	1.11	[0.19; 2.04]	64.4	-	[-0.4; 2.63]	2.46
Trim-and-fill method	5	1.08	[0.3; 1.86]	60.46	[0; 85.19]	[-0.57; 2.73]	2.54

S6. *Effects of purely unguided interventions*

	k	*g*	95%-CI	I^2	95%-PI	NNT
Depression (8 studies)	25	0.47	[0.14; 0.79]	85.9	[-0.27; 1.20]	6.51
Insomnia (6 studies)	6	0.74	[0.15; 1.34]	87.6	[-0.83; 2.31]	3.83
PTSD (3 studies)	3	0.19	[-0.35; 0.74]	0.0	[-0.28; 0.67]	17.03

Note. For GAD, panic disorder, OCD, specific phobias and SAD, not enough studies ($k \geq 3$) were available for pooling. We coded interventions as "purely" unguided if no mechanism for (human or automatized) encouragement was reported (e.g., motivational messages, e-mail reminders, prescheduled telephone calls, visits to monitor adherence)

S7. *Dropout rates per arm and disorder.*

	All Interventions						Guided Interventions						Unguided Interventions					
	n_{eff}	Prop.	95%-CI	95%-PI	I^2	τ^2	n_{eff}	Prop.	95%-CI	95%-PI	I^2	τ^2	n_{eff}	Prop.	95%-CI	95%-PI	I^2	τ^2
Depression (k=49)																		
IG	49	17%	[13%; 21.9%]	[2.9%; 58.2%]	89.7	0.931	35	17.4%	[13%; 23%]	[3.6%; 54.6%]	86.6	0.756	14	16.1%	[8.9%; 27.3%]	[1.7%; 67.3%]	93.2	1.351
CG	49	14.9%	[10.5%; 20.7%]	[1.4%; 67.8%]	92.6	1.57	35	14.2%	[9.4%; 21%]	[1.4%; 66.2%]	91.9	1.526	14	16.7%	[8.6%; 29.8%]	[1.4%; 73.4%]	93.4	1.643
Insomnia (k=37)																		
IG	37	16.5%	[11.2%; 23.5%]	[1.7%; 69.5%]	97.2	1.507	13	11.9%	[5.5%; 23.6%]	[1%; 64.5%]	87.3	1.578	24	19%	[12.4%; 28%]	[2.2%; 70.8%]	97.7	1.354
CG	37	10.6%	[6.8%; 16.2%]	[0.9%; 61.1%]	96.3	1.675	13	9.3%	[3.1%; 24.8%]	[0.3%; 80.3%]	91.7	3.172	24	11.5%	[7.4%; 17.3%]	[1.6%; 51.1%]	95.7	1.078
Social Anxiety Disorder (k=26)																		
IG	26	11.6%	[7.4%; 17.8%]	[1.4%; 54.5%]	85.1	1.208	20	10.5%	[6.3%; 16.9%]	[1.4%; 48.6%]	82.1	1.06	6	16.1%	[6.3%; 35.6%]	[1.4%; 71.7%]	88.2	1.443
CG	26	8.8%	[5.5%; 13.9%]	[1%; 47.2%]	79.1	1.219	20	7.5%	[4.1%; 13.2%]	[0.7%; 47%]	80	1.39	6	13.4%	[6.6%; 25.3%]	[2.6%; 47.5%]	69.7	0.649
Posttraumatic Stress Disorder (k=15)																		
IG	15	19.3%	[11.2%; 31.2%]	[2.2%; 71.9%]	90.1	1.357	12	20.3%	[10.3%; 36.1%]	[1.7%; 79%]	92.1	1.724	3	17.2%	[11.6%; 24.7%]	[11.6%; 24.7%]	0	0
CG	15	12.2%	[8%; 18.3%]	[2.9%; 39.6%]	71.9	0.568	12	11.9%	[6.8%; 20.1%]	[2%; 47.6%]	78.8	0.842	3	12.5%	[7.8%; 19.4%]	[7.8%; 19.4%]	0	0
Panic Disorder (k=15)																		
IG	15	0%	[0%; 82.1%]	[0%; 100%]	98.7	77.653	14	0%	[0%; 87.3%]	[0%; 100%]	98.6	71.674	-	-	-	-	-	-
CG	15	0%	[0%; 82.7%]	[0%; 98.5%]	92.6	15.789	14	0%	[0%; 48.2%]	[0%; 93.5%]	91.4	13.202	-	-	-	-	-	-
Generalized Anxiety Disorder (k=12)																		
IG	13	17.5%	[8.7%; 32.1%]	[1.6%; 73.8%]	88.1	1.575	10	13.3%	[9.4%; 18.4%]	[6.4%; 25.5%]	37.6	0.13	3	53%	[5.5%; 95.6%]	[0.7%; 99.4%]	95.1	4.33
CG	13	9.7%	[3.5%; 24%]	[0.4%; 76.5%]	88.6	2.728	10	9.2%	[5.9%; 14.2%]	[3.7%; 21.2%]	35.1	0.185	3	6.2%	[0%; 99.8%]	[0%; 100%]	96	27.135
Obsessive-Compulsive Disorder (k=9)																		
IG	9	13.9%	[5.9%; 29.3%]	[1.2%; 68.8%]	89	1.546	5	12%	[3.6%; 33.6%]	[0.8%; 70.4%]	84.2	1.676	4	16.8%	[5.2%; 42.7%]	[1.5%; 72.7%]	86.6	1.289
CG	9	6.7%	[2.1%; 19.4%]	[0.3%; 61.3%]	88.3	2.109	5	1.6%	[0%; 41.9%]	[0%; 94.4%]	95	8.826	4	11.7%	[7.6%; 17.7%]	[7.6%; 17.7%]	0	0
Specific Phobia (k=5)																		
IG	5	0.6%	[0%; 69.2%]	[0%; 95.6%]	87	8.257	-	-	-	-	-	-	2	7.8%	[0.4%; 67%]	[0.1%; 86.3%]	65.8	2.205

Note. IG = interventions groups; CG = control groups.

S8. *Differential dropout rates per disorder.*

		k	*RR*	95%-CI	95%-PI	I^2	τ^2
Depression	All	49	1.14	[0.94; 1.37]	[0.48; 2.72]	64.4	0.189
	Unguided	14	1.10	[0.76; 1.59]	[0.38; 3.20]	72.1	0.263
	Guided	35	1.15	[0.93; 1.42]	[0.52; 2.53]	57.1	0.151
Insomnia	All	37	1.45	[1.21; 1.75]	[0.72; 2.93]	73.3	0.119
	Unguided	24	1.60	[1.30; 1.96]	[0.79; 3.21]	77.6	0.116
	Guided	13	0.97	[0.78; 1.21]	[0.78; 1.21]	0	0
Social Anxiety Disorder	All	26	1.24	[0.93; 1.66]	[0.61; 2.55]	26.3	0.112
	Unguided	6	1.20	[0.58; 2.48]	[0.28; 5.23]	55.9	0.426
	Guided	20	1.20	[0.87; 1.65]	[0.64; 2.26]	18.3	0.077
Posttraumatic Stress Disorder	All	15	1.78	[1.42; 2.23]	[1.42; 2.23]	0	0
	Unguided	3	1.40	[0.77; 2.56]	[0.77; 2.56]	0	0
	Guided	12	1.85	[1.45; 2.37]	[1.45; 2.37]	0	0
Panic Disorder	All	15	2.06	[1.13; 3.75]	[1.13; 3.75]	0	0
	Guided	14	2.10	[1.15; 3.86]	[1.15; 3.86]	0	0
Generalized Anxiety Disorder	All	13	1.13	[0.89; 1.43]	[0.81; 1.59]	6.1	0.016
	Unguided	3	1.89	[0.27; 13.14]	[0.09; 37.74]	55.6	1.356
	Guided	10	1.38	[0.91; 2.11]	[0.91; 2.11]	0	0
Obsessive-Compulsive Disorder	All	9	1.68	[1.22; 2.33]	[1.13; 2.51]	4.9	0.014
	Unguided	4	2.16	[1.31; 3.56]	[1.31; 3.56]	0	0
	Guided	5	1.44	[1.00; 2.07]	[1.00; 2.07]	0	0
Specific Phobia	All	5	2.66	[0.46; 15.27]	[0.46; 15.27]	0	0
	Guided	3	0.98	[0.06; 15.03]	[0.06; 15.03]	0	0

Note. Values of $RR>1$ indicate higher dropout in the intervention groups.

S9. *Results of moderator analyses across disorders (variable-by-variable).*

	$\hat{\beta}$	S.E.	t	p
World Region				
Europe	*Ref.*			
East Asia	0.135	0.143	0.945	0.345
Australia	0.225	0.104	2.168	0.031
Middle East	0.387	0.239	1.618	0.107
North America	0.017	0.096	0.177	0.860
Intervention Format				
Cognitive Behavior Therapy	*Ref.*			
Behavioral Activation	-0.019	0.224	-0.087	0.931
Psychodynamic Therapy	-0.040	0.135	-0.299	0.765
Exposure Therapies	-0.192	0.200	-0.956	0.340
Other Therapies	-0.576	0.205	-2.816	0.005
Problem-Solving Therapy	-0.065	0.332	-0.196	0.845
Third-Wave Therapies	-0.141	0.156	-0.903	0.367
Comparator				
Care As Usual	*Ref.*			
Other Controls	0.018	0.142	0.129	0.898
Psychoeducation	0.131	0.209	0.625	0.533
Placebo	0.146	0.253	0.579	0.563
Waitlist Controls	0.102	0.113	0.897	0.371
Risk of Bias				
"High" or "Some Concerns"	*Ref.*			
"Low"	0.103	0.107	0.960	0.338
Publication Year[a]	0.001	0.046	0.023	0.982
Females (% in sample)[a]	0.057	0.033	1.723	0.086
Age (sample mean)[a]	-0.038	0.037	-1.045	0.297
Recruitment				
Clinical	*Ref.*			
Community	0.280	0.133	2.099	0.037
Other	0.029	0.188	0.154	0.878
Guidance				
Guided interventions	*Ref.*			
Unguided interventions	-0.034	0.062	-0.559	0.577
Intervention Modality				
Internet/Computer-based[b]	*Ref.*			
Mobile/Smartphone-based	-0.190	0.106	-1.797	0.073
Other Modalities	-0.568	0.293	-1.937	0.053
Virtual Reality-based	-0.373	0.175	-2.128	0.034

Note. Results based separate meta-regression models for each moderator (all models stratified by disorder). *Ref.* = reference category. [a]Centered and scaled; [b]Includes Internet- and mobile-based interventions (IMIs).

S10. *Results of GRADE assessment.*

			Certainty Assessment					№ of patients		Effect	Certainty
Outcome	№ studies	Study design	Risk of Bias	Inconsis-tency	Indirectness	Imprecision	Other Considerations	Intervention	Inactive Control	Absolute (95% CI)	
Depression	49	randomised trials	serious[a]	serious[b]	not serious[c]	not serious	publication bias strongly suspected[d]	3461	2865	SMD **0.62 SD lower** (0.49 lower to 0.74 lower)	⊕◯◯◯ Very low[a,b,c,d]
Insomnia	37	randomised trials	serious[e]	serious[b]	not serious[c]	not serious	none	5032	4226	SMD **0.94 SD lower** (0.82 lower to 1.07 lower)	⊕⊕◯◯ Low[b,c,e]
SAD	26	randomised trials	serious[e]	not serious[i]	not serious[c]	not serious	none	1212	886	SMD **0.84 SD lower** (0.68 lower to 0.99 lower)	⊕⊕⊕◯ Moderate[e,i]
PTSD	15	randomised trials	serious[e]	serious[b]	not serious[c]	serious[f]	none	913	772	SMD **0.57 SD lower** (0.28 lower to 0.85 lower)	⊕◯◯◯ Very low[b,c,e,f]
Panic	15	randomised trials	serious[e]	serious[b]	not serious[c]	not serious	publication bias strongly suspected[d]	491	426	SMD **1.05 SD lower** (0.78 lower to 1.32 lower)	⊕◯◯◯ Very low[b,c,d,e]
GAD	12	randomised trials	serious[e]	serious[g]	not serious[c]	not serious	none	508	425	SMD **0.8 SD lower** (0.45 lower to 1.16 lower)	⊕⊕◯◯ Low[c,e,g]
OCD	9	randomised trials	very serious[h]	not serious[i]	not serious[c]	not serious	none	368	371	SMD **0.68 SD lower** (0.49 lower to 0.86 lower)	⊕⊕◯◯ Low[c,i,h]
Spec. Phobia	5	randomised trials	serious[e]	not serious[i]	not serious[c]	not serious	none	94	94	SMD **1.18 SD lower** (0.52 lower to 1.85 lower)	⊕⊕⊕◯ Moderate[c,e,i]

Explanations

[a]The results from trials with low and high RoB are concordant. However, the number of studies at high RoB is very high. Due to the study design, blinding of participants is not possible and in most studies self-reported outcomes are assessed. There are some studies raising significant concerns about sequence generations, allocation concealment and appropriate ITT analyses; [b]There is substantial heterogeneity indicated by high I^2 values and wide prediction intervals, which cannot be explained in sensitivity analyses; [c]The intervention is delivered different in different settings, i.e. there are some countries that have prescription digital therapeutics that might differ from other digital interventions in terms of availability and implementation. Additionally, across all disorders, effects differ by world region, intervention format, type of recruitment, and intervention modality. However, we did not deem these differences substantial enough to downgrade the certainty of evidence; [d]Sensitivity analyses suggest the presence of publication bias. Additionally, grey literature was not searched; [e]The results from trials with low and high RoB are discordant due to high imprecision in sensitivity analyses. The proportion of information from studies at high risk of bias is sufficient to affect the interpretation of results. Outcome assessors are not blinded in many trials (self-report); [f]The confidence interval does not exclude the possibility of no important effect, as it crosses the threshold for minimal clinically important benefit; [g]There is substantial heterogeneity, which can only partly be explained in sensitivity analyses; [h]There is no comparison with low risk of bias, which substantially lowers the confidence in the effect estimates; [i]There is low to moderate heterogeneity, which can largely be explained in sensitivity analyses. CI: confidence interval; SMD: standardized mean difference. **Author(s):** MH, PK. **Question:** Digital psychological interventions compared to inactive control conditions for treating mental disorders (depression, insomnia, social anxiety disorder, PTSD, panic, GAD, OCD, specific phobias). **Setting:** Participants with diagnosed mental disorders, confirmed by clinical interviews.

S11. *Number of digital intervention trials per country.*

Country	Number of trials	Per Capita
Sweden	26	2.583
Australia	28	1.138
Netherlands	16	0.934
Switzerland	7	0.827
Finland	3	0.544
Norway	2	0.379
Denmark	1	0.173
Germany	14	0.169
Canada	6	0.163
Spain	7	0.150
United Kingdom	8	0.121
Jordan	1	0.103
Romania	2	0.102
United States	29	0.089
Iraq	2	0.052
Korea (Republic of)	2	0.039
Malaysia	1	0.032
Saudi Arabia	1	0.030
Iran (Islamic Republic)	2	0.025
Japan	3	0.024
Egypt	1	0.010
China	6	0.004
Indonesia	1	0.004

Note. "Per capita" gives the number of digital intervention trials per one million inhabitants. These numbers were derived using world population estimates for 2017 (World Bank; "sp.pop.totl" database).
